# Identification of genes associated with the biosynthesis of unsaturated fatty acid and oil accumulation in herbaceous peony ‘Hangshao’ (*Paeonia lactiflora* ‘Hangshao’) seeds based on transcriptome analysis

**DOI:** 10.1186/s12864-020-07339-7

**Published:** 2021-02-01

**Authors:** Jia-Song Meng, Yu-Han Tang, Jing Sun, Da-Qiu Zhao, Ke-Liang Zhang, Jun Tao

**Affiliations:** 1 College of Horticulture and Plant Protection, Yangzhou University, Yangzhou, 225009 Jiangsu China; 2 Joint International Research Laboratory of Agriculture and Agri-Product Safety, the Ministry of Education of China, Yangzhou University, Yangzhou, 225009 Jiangsu China; 3 College of Animal Science and Technology, Yangzhou University, Yangzhou, 225009 Jiangsu China

**Keywords:** *Paeonia lactiflora* ‘Hangshao’, Fatty acid biosynthesis, Transcriptome, Differentially expressed gene, qRT-PCR

## Abstract

**Background:**

*Paeonia lactiflora* ‘Hangshao’ is widely cultivated in China as a traditional Chinese medicine ‘Radix Paeoniae Alba’. Due to the abundant unsaturated fatty acids in its seed, it can also be regarded as a new oilseed plant. However, the process of the biosynthesis of unsaturated fatty acids in it has remained unknown. Therefore, transcriptome analysis is helpful to better understand the underlying molecular mechanisms.

**Results:**

Five main fatty acids were detected, including stearic acid, palmitic acid, oleic acid, linoleic acid and α-linolenic acid, and their absolute contents first increased and then decreased during seed development. A total of 150,156 unigenes were obtained by transcriptome sequencing. There were 15,005 unigenes annotated in the seven functional databases, including NR, NT, GO, KOG, KEGG, Swiss-Prot and InterPro. Based on the KEGG database, 1766 unigenes were annotated in the lipid metabolism. There were 4635, 12,304, and 18,291 DEGs in Group I (60 vs 30 DAF), Group II (90 vs 60 DAF) and Group III (90 vs 30 DAF), respectively. A total of 1480 DEGs were detected in the intersection of the three groups. In 14 KEGG pathways of lipid metabolism, 503 DEGs were found, belonging to 111 enzymes. We screened out 123 DEGs involved in fatty acid biosynthesis (39 DEGs), fatty acid elongation (33 DEGs), biosynthesis of unsaturated fatty acid (24 DEGs), TAG assembly (17 DEGs) and lipid storage (10 DEGs). Furthermore, qRT-PCR was used to analyze the expression patterns of 16 genes, including *BBCP*, *BC*, *MCAT*, *KASIII*, *KASII*, *FATA*, *FATB*, *KCR*, *SAD*, *FAD2*, *FAD3*, *FAD7*, *GPAT*, *DGAT*, *OLE* and *CLO*, most of which showed the highest expression at 45 DAF, except for *DGAT*, *OLE* and *CLO*, which showed the highest expression at 75 DAF.

**Conclusions:**

We predicted that *MCAT*, *KASIII*, *FATA*, *SAD*, *FAD2*, *FAD3*, *DGAT* and *OLE* were the key genes in the unsaturated fatty acid biosynthesis and oil accumulation in herbaceous peony seed. This study provides the first comprehensive genomic resources characterizing herbaceous peony seed gene expression at the transcriptional level. These data lay the foundation for elucidating the molecular mechanisms of fatty acid biosynthesis and oil accumulation for herbaceous peony.

**Supplementary Information:**

The online version contains supplementary material available at 10.1186/s12864-020-07339-7.

## Background

As a well-known Chinese traditional flower, herbaceous peony (*Paeonia lactiflora* Pall.) was cultivated more than 4000 years. Owing to the beauty of the color, shape and fragrance, it is regarded as ‘the Prime-Minister of flowers’ and is known as the ‘two superb of flowers’ with tree peony (*Paeonia suffruticosa* Andr.), which is regarded as ‘the King of flowers’. Herbaceous peony is cultivated as a medicinal and ornamental plant in China. To date, more than 500 cultivars are distributed in different regions of China [1]. The dried roots of two herbaceous peony species are the main raw materials for the traditional Chinese medicine ‘Radix Paeoniae Alba’ and ‘Radix Paeoniae Rubra’ [2, 3], which have antithrombic [4], antitumor [5], and anti-posttraumatic stress disorder functions [6]; alleviate inflammation [7]; remove pathogenic heat from the blood; treat blood stasis; relieve pain [8], and so on. As an important medicinal plant, herbaceous peony ‘Hangshao’, the root of which is the traditional Chinese medicine ‘Radix Paeoniae Alba’, is mainly cultivated in Zhejiang Province and widely cultivated in China [9]. In addition, it has a single lobe and a higher seed-setting rate. However, the tremendous number of seeds from herbaceous peony ‘Hangshao’ are mainly wasted every year, except the handful of seeds that are used for propagation [10]. Previous studies have shown that the seed oil content of ‘Hangshao’ is approximately 25%, with more than 90% unsaturated fatty acids (UFAs) [11]. Therefore, herbaceous peony ‘Hangshao’ has the potential to be cultivated as a multifunctional plant, not only as an ornamental, medicinal plant but also as an edible oil resource.

Herbaceous peony ‘Hangshao’ seed oil predominantly contains five fatty acids: oleic acid (C18:1^Δ9^, OA), linoleic acid (C18:2^Δ9,12^, LA), α-linolenic acid (C18:3^Δ9,12,15^, ALA), palmitic acid (C16:0, PA) and stearic acid (C18:0, SA) [10, 11]. OA, LA and ALA are classified as UFAs, which include monounsaturated fatty acids (MUFAs) and polyunsaturated fatty acids (PUFAs). OA, which can improve diastolic heart function, is a MUFA [12]. LA and ALA are PUFAs, which can reduce thrombosis, decrease cardiovascular events and prevent cancer [13]. According to the location of the last double carbon bond relative to the terminal methyl end of the molecule, PUFAs can be divided into the omega-3 (n-3) series and omega-6 (n-6) series. ALA, an important omega-3 PUFA, is an essential fatty acid that cannot be synthesized in the human body and must be obtained via dietary intake; it is the precursor of docosahexaenoic acid (DHA) and eicosapentaenoic acid (EPA), which are essential for the growth and development of the brain and retina [14]. Additionally, LA, an important omega-6 PUFA, is also an essential fatty acid that cannot be produced by the human body; it is the precursor of the omega-6 series of fatty acids such as γ-linolenic acid (C18:3^Δ6,9,12^, GLA) and arachidonic acid (C18:4^Δ5,8,11,14^, AA) [15]. Nevertheless, high intake of omega-6 PUFAs with low intake of omega-3 PUFAs might promote the pathogenesis of many diseases, including cardiovascular disease, cancer, and inflammatory and autoimmune diseases [16]. Therefore, the Food and Agriculture Organization (FAO) of the United Nations and the World Health Organization (WHO) suggest that the omega-6/3 ratio should be lower than 5:1 [17].

Previous research has shown that the ratio of omega-6/omega-3 is approximately 1.5:1 in herbaceous peony ‘Hangshao’ mature seed oil. However, the ratio of omega-6/omega-3 increases during seed development because the relative content of ALA decreases gradually and the relative content of LA slightly increases [10, 11]. Nevertheless, very little research has examined fatty acid accumulation at the molecular level for herbaceous peony seed oil. Fatty acid biosynthesis in plants occurs via a very complicated biological pathway regulated by various enzymes. RNA sequencing (RNA-Seq) has provided a convenient and rapid method for finding key genes in fatty acid biosynthesis and unsaturated fatty acid biosynthesis at the molecular level. De novo transcriptome sequencing had been applied to identify and profile gene expression differences of some herb oilseed plants such as soybean [18], peanut [19], rape [20], and sunflower [21] and some tree oilseed plants such as tree peony [17], olive [22], tea-oil camellia [23], and *Hiptage benghalensis* [24]. However, research on herbaceous peony has mainly concentrated on thermotolerant-related differentially expressed genes [25], codon usage patterns [26] and anthocyanin biosynthetic genes [27] based on transcriptome sequencing. The fatty acid metabolism of herbaceous peony has remained unknown. Moreover, genetic control of the unsaturated fatty acid accumulation of herbaceous peony is unexplored.

In this study, we measured the oil content and absolute content of five main fatty acids of herbaceous peony ‘Hangshao’ seeds at five developmental stages (30, 45, 60, 75 and 90 days after flowering, DAF) by gas chromatography-mass spectrometry (GC-MS). We identified differentially expressed genes (DEGs) related to the biosynthesis of unsaturated fatty acids and oil accumulation. Furthermore, we validated the expression of 16 DEGs using quantitative real-time polymerase chain reaction (qRT-PCR). Our results reveal the transcriptomic aspects of unsaturated fatty acid biosynthesis and oil accumulation in herbaceous peony seed, and provide a useful reference for fatty acid biosynthesis studies of other plant species.

## Results

### Change in oil content and absolute contents of five fatty acids

**Fig. 1:**
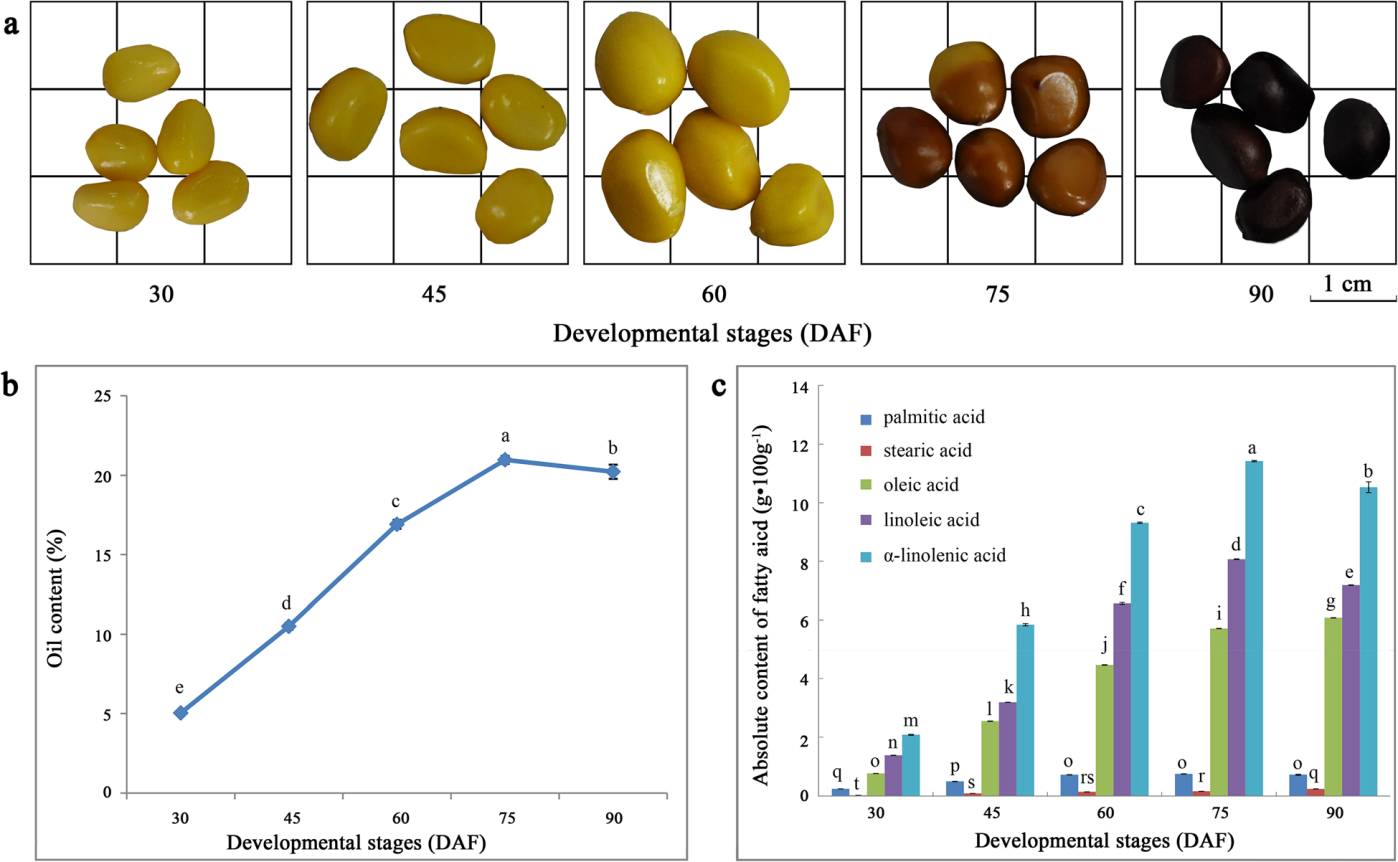
Oil content and absolute content of five main fatty acids in seeds of *Paeonia lactiflora* ‘Hangshao’. **a** Seeds in the collecting period; **b** Oil content in the developing seed; **c** Absolute content of five main fatty acids in the developing seed. DAF, days after flowering

The oil content of herbaceous peony ‘Hangshao’ seeds at five different stages was studied. It was approximately 5.06%±0.06% at 30 DAF, and it increased constantly, reaching a maximum of 20.92%±0.25% at 75 DAF and then slightly decreasing at 90 DAF (Fig. 1b). Five main fatty acids, PA, SA, OA, LA and ALA, were detected by gas chromatography-mass spectrometry (GC-MS) analysis of the fatty acid components of the seeds from the herbaceous peony ‘Hangshao’. Through quantitative analysis of the contents of five fatty acids (Fig. 1c), it was found that the content of ALA, LA and PA showed a trend toward first rising and then falling during seed development, and the maximum value appeared in the S4 period. In contrast, the content of OA and SA increased consistently during seed development, and the maximum value appeared in the S5 period. However, the absolute contents of the five fatty acids in the five periods showed that the ALA content was highest and the SA content was lowest, and the contents followed the order ALA > LA > OA > PA > SA. The total contents of the five fatty acids from S1 to S5 seeds were 4.49 g·100 g^− 1^, 12.17 g·100 g^− 1^, 21.19 g·100 g^− 1^, 26.11 g·100 g^− 1^ and 24.75 g·100 g^− 1^. Moreover, the content of ALA exceeded 10 g·100 g^− 1^ in both the S4 and S5 stages.

### Sequencing read filtering and de novo assembly

Nine cDNA libraries containing three biological repeats were created from three stages of developing herbaceous peony ‘Hangshao’ seeds (the early, middle and late stages of seed development, that is 30, 60, and 90 DAF, respectively) collected from the same strain and sequenced using the Illumina high-throughput sequencing platform, to explore the molecular mechanism of fatty acid biosynthesis and oil accumulation. A total of 100.07 G paired-end raw reads were produced by transcriptome sequencing. After removing low-quality, adapter-contaminated and high-content unknown base (N) reads, the percentage of clean reads for nine samples was more than 82% (Additional file 1: Table S1). After read filtering and assembly, a set of 150,156 unigenes was obtained, with a total length, average length, and N50 and GC content of 154,674,160 bp, 1030 bp, 1825 bp and 39.97%, respectively (Additional file 2: Table S2). The unigene length ranged from 300 to 3000 nt, and the unigene number gradually decreased without obvious separation as the size of the sequences increased (Additional file 3: Figure S1).

### Gene functional annotation

After assembly, all the unigenes were subjected to BLASTx analysis against seven public databases, including NR, NT, GO, KOG, KEGG, Swiss-Prot and InterPro (Table 1). A total of 73,399 (48.88%) unigenes were annotated in at least one database, whereas 15,005 (9.99%) unigenes were annotated in all databases. The highest annotation rate was obtained in the NR database, which assigned 67,096 (44.68%) unigenes. A total of 45,488 (30.29%), 42,533 (28.33%) and 48,907 (32.57%) unigenes were annotated using the NT, Swiss-Prot and InterPro database, respectively.

**Table 1:** Functional annotation of unigenes for seeds of *Paeonia lactiflora* ‘Hangshao’ in a public functional database

Values	Number	Percentage
Total unigenes	150,156	100%
Nr	67,096	44.68%
Nt	45,488	30.29%
Swissprot	42,533	28.33%
KEGG	49,630	33.05%
KOG	51,612	34.37%
Interpro	48,907	32.57%
GO	29,413	19.59%
Intersection	15,005	9.99%
Overall	73,399	48.88%

Based on the NR database function annotation result, the ratio of different species unigene annotation was calculated, and the distribution map was drawn (Additional file 4:Figure S2A). The unigenes of herbaceous peony ‘Hangshao’ seeds were matched to species such as *Vitis vinifera* (32.79%), *Nelumbo nucifera* (5.84%), *Theobroma cacao* (5.45%) and *Jatropha curcas* (3.66%). In addition, 51,612 (34.37%) unigenes were annotated with 25 KOG terms (Additional file 4: Figure S2B). Interestingly, 1868 unigenes were annotated in the ‘Lipid transport and metabolism’ category. Moreover, 29,413 (19.59%) unigenes were annotated with 55 GO terms, including 24 terms related to biological processes, 17 terms related to cellular component and 14 terms related to molecular functions (Additional file 4:Figure S2C). To further understand the biological functions and interactions of genes, they were also classified into metabolic pathways using KEGG. A total of 49,630 (33.05%) unigenes were assigned to 21 subcategories divided into six categories, including cellular processes, environmental information processing, genetic information processing, human diseases, metabolism and organismal systems (Additional file 4:Figure S2D). Among the KEGG pathways, the top three subcategories were ‘global and overview maps’ (12,390 unigenes), metabolism’ (4905 unigenes) and ‘translation’ (4321 unigenes). In contrast, 1766 unigenes were annotated to ‘lipid metabolism’, which will help us understand the fatty acid and oil accumulation in herbaceous peony seeds.

### Analysis of differentially expressed genes for developmental herbaceous peony ‘Hangshao’ seeds

**Fig. 2:**
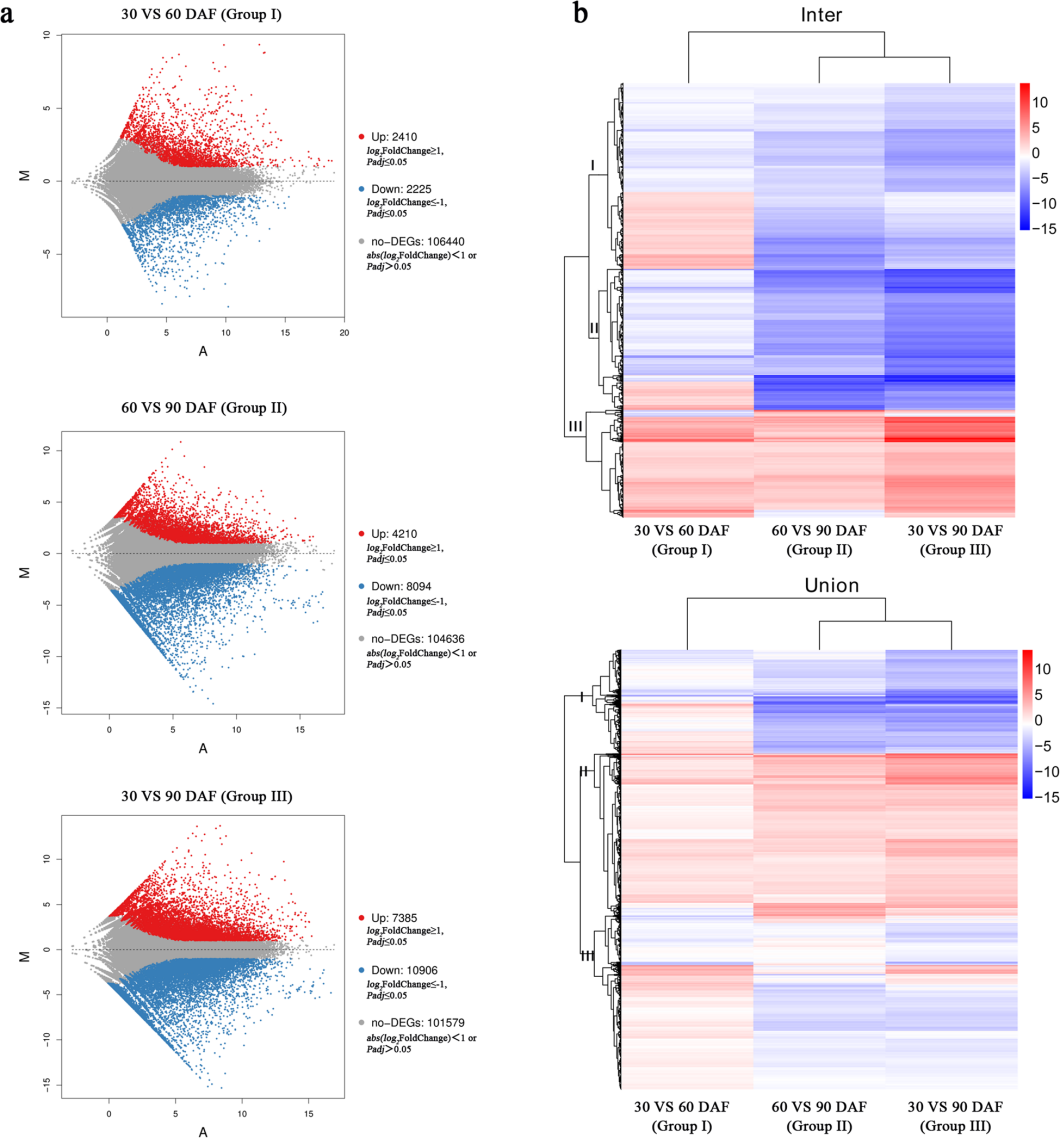
Analysis of DEGs in seeds of *Paeonia lactiflora* ‘Hangshao’**. a** Gene analysis of DEGs in different stages; **b** Cluster analysis of DEGs in different stages

To understand the patterns of seed development in detail and identify key genes in fatty acid biosynthesis, differentially expressed genes (DEGs) were analyzed by comparing the FPKM value of unigenes between the different developmental seeds of the herbaceous peony ‘Hangshao’. Based on the gene expression level, DEGs were identified between different groups (Fig. 2a). Seeds at 30 DAF served as the control, and 4635 and 18,291 DEGs were identified at 60 and 90 DAF, respectively. Additionally, seeds at 60 DAF served as the control, and 12,304 DEGs were also identified at 90 DAF. There were 2410, 4210 and 7385 upregulated unigenes and 2225, 8094 and 10,906 downregulated unigenes at 30 vs 60 DAF (Group I), 60 vs 90 DAF (Group II) and 30 vs 90 DAF (Group III), respectively. Two hierarchical cluster analyses were conducted based on the FPKM value of these DEGs for the three groups (Fig. 2b). The DEG intersection (Inter) and DEG union of the three different groups were clustered into three clusters. DEGs in the same cluster had identical or similar expression patterns during the seed developmental stage.

The DEGs at different developmental stages were integrated and mapped into the GO database (Additional file 5: Table S3 and Additional file 6: Figure S3). Overall, the number of DEGs annotated to the same GO term between the three different groups increased with the developmental stage. The DEGs in Group III were annotated to 23 biological processes, 17 cellular components and 14 molecular functions. In contrast, the DEGs in Group II were annotated to 21 biological processes (except for ‘biological adhesion’ and ‘locomotion’), 17 cellular components and 13 molecular functions (except for ‘metallochaperone activity’). Additionally, the DEGs in Group I were annotated to 21 biological processes (except for ‘biological adhesion’ and ‘rhythmic process’), 14 cellular components (except for ‘nucleoid’, ‘virion’ and ‘virion part’) and 12 molecular functions (except for ‘metallochaperone activity’ and ‘protein tag’).

**Fig. 3:**
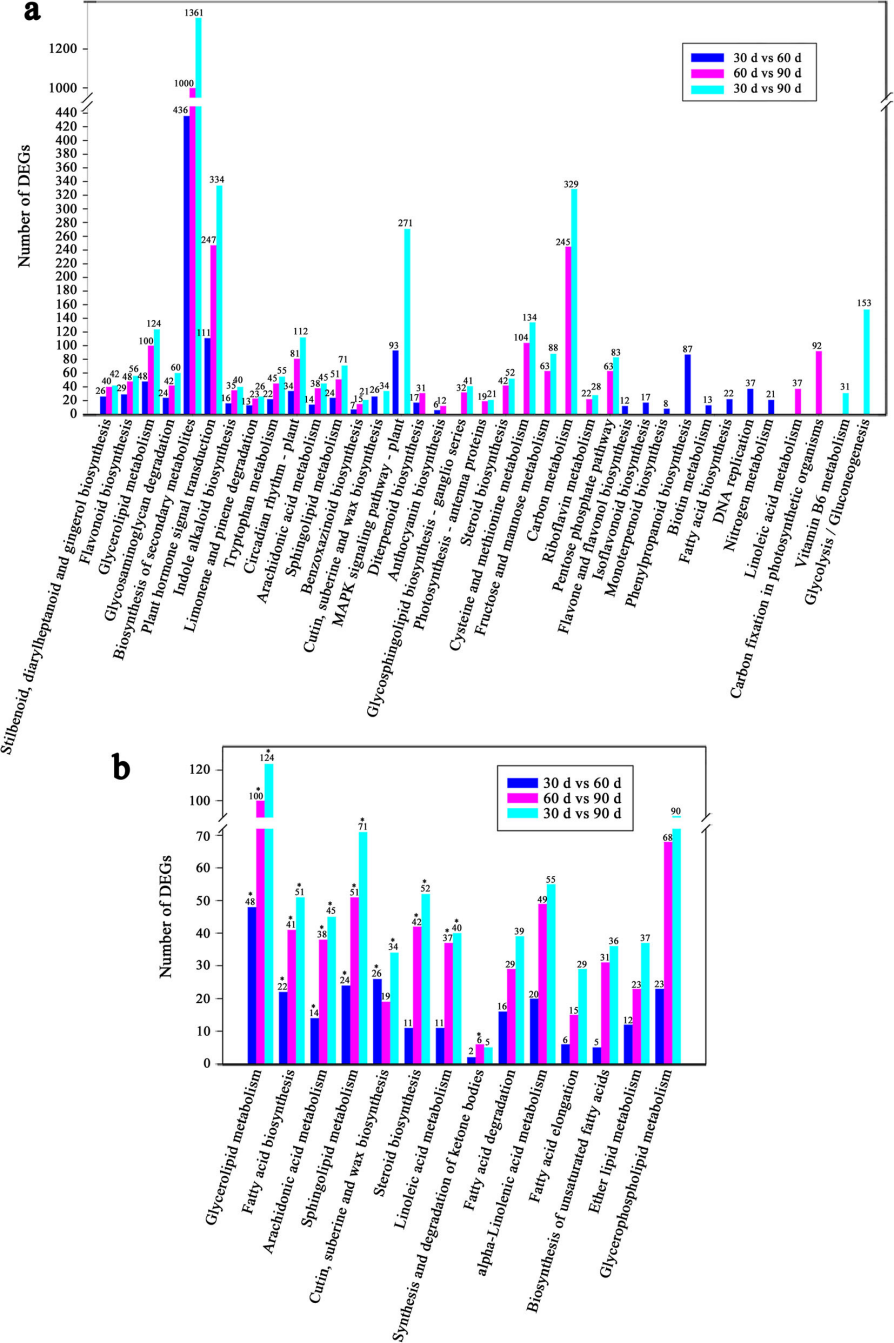
Analysis of DEGs in significantly enriched KEGG pathways. **a** Number of DEGs in the top 25 significantly enriched KEGG pathways; **b** Number of DEGs in the l4 lipid metabolism KEGG pathways; * represents a significantly enriched KEGG pathway

DEGs in the three different groups were annotated to five KEGG categories (including cellular processes, environmental information processing, genetic information processing, metabolism and organismal systems) and 19 KEGG subcategories (Additional file 7: Table S4). The numbers of DEGs annotated to the KEGG pathway were 1698, 4321 and 6035 for Group I, Group II and Group III, respectively (Additional file 8: Table S5). Further analysis of the KEGG annotation showed that 28, 43 and 41 pathways were significantly enriched for Group I, Group II and Group III, respectively (Additional file 9: Table S6). Among the top 25 enriched pathways in each group, 13 pathways were significantly enriched in all three groups (Fig. 3a). Among them, glycerolipid metabolism, arachidonic acid metabolism and sphingolipid biosynthesis belong to lipid metabolism. Additionally, 12 pathways were significantly enriched in two groups, including cutin, suberine and wax biosynthesis and steroid biosynthesis, which belong to lipid metabolism. Moreover, 12 pathways were significantly enriched in only one group, including fatty acid biosynthesis and linoleic acid metabolism. Additionally, 14 pathways were annotated to lipid metabolism (Fig. 3b and Additional file 10:Table S7). Glycerolipid metabolism, fatty acid biosynthesis, arachidonic acid metabolism and sphingolipid metabolism were significantly enriched in all three groups. The number of DEGs in the 4 KEGG pathways mentioned above increased with seed development. Despite no significant enrichment of DEGs in the KEGG pathway of unsaturated fatty acid biosynthesis, the number of DEGs also increased with seed development. These results provide clues regarding the identification of key genes involved in the biosynthesis of unsaturated fatty acids.

**Fig. 4:**
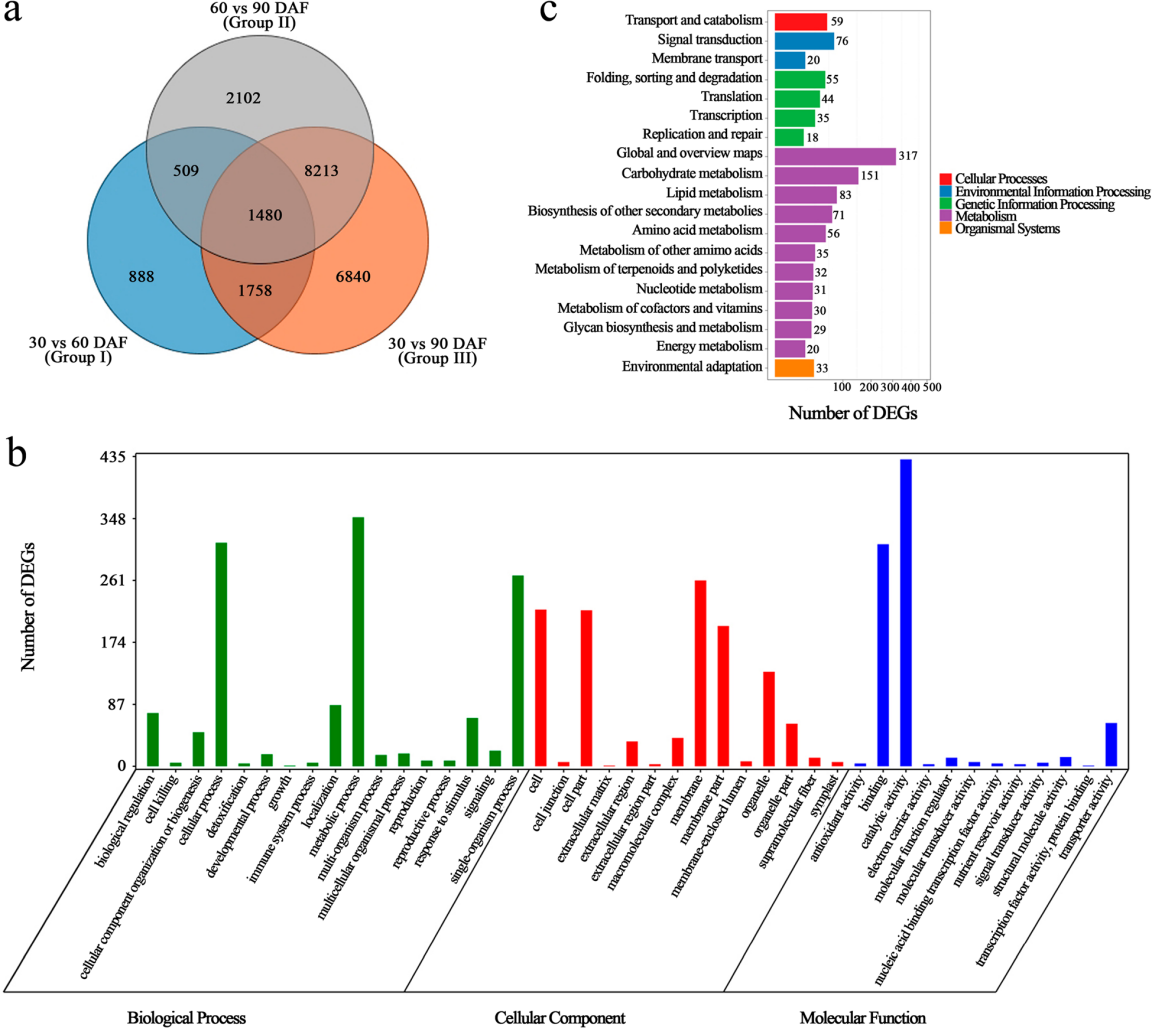
Analysis of DEGs obtained from the intersection of three groups. **a** Venn diagram between DEGs from three groups; **b** Functional distribution of intersection DEGs annotated by GO; **c** Functional distribution of intersection DEGs annotated by KEGG

In addition, 1480 DEGs were obtained from the intersection analysis of the three groups (Fig. 4a), and these 1480 DEGs were annotated to 17 biological processes, 14 cellular components and 12 molecular functions in the GO database (Fig. 4b). In biological process, the largest category was ‘metabolic process’, followed by ‘cellular process’ and ‘single-organism process’. In cellular component, the largest category was ‘membrane’, followed by ‘cell’ and ‘cell part’. In molecular function, the two largest categories were ‘catalytic activity’ and ‘binding’. Moreover, the 1480 DEGs were annotated to 19 KEGG pathways (Fig. 4c). The largest category was ‘global and overview maps’, followed by ‘carbohydrate metabolism’ and ‘lipid metabolism’.

### Analysis of DEGs for lipid metabolism

**Fig. 5:**
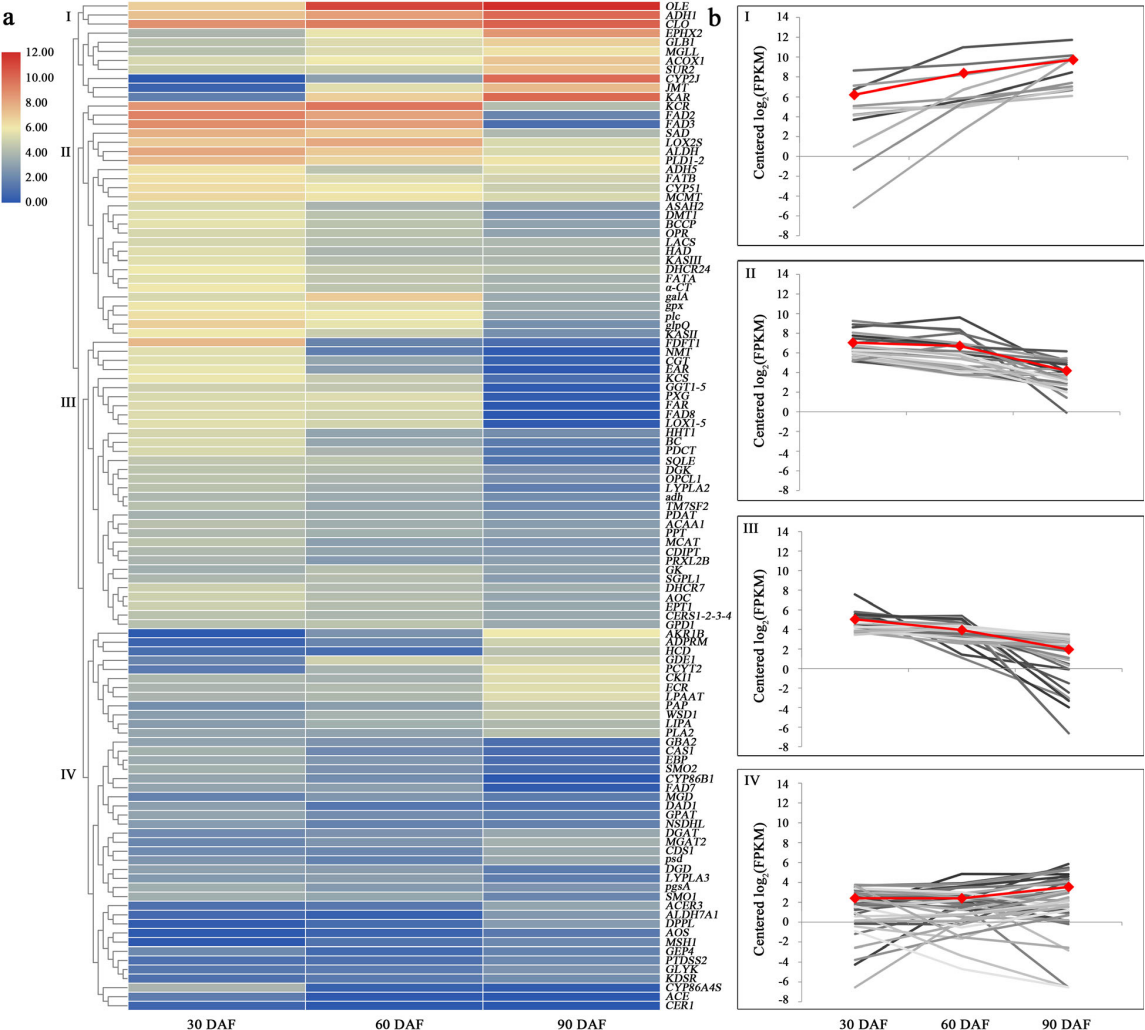
Cluster analysis of DEGs in the lipid metabolism pathway of *Paeonia lactiflora* ‘Hangshao’ seeds. **a** Hierarchical clustering dendrogram of DEGs in lipid metabolism. The red highlighting indicates genes that were highly expressed, whereas the blue highlighting indicates genes with low expression. **b** The four cluster groups with different gene expression patterns

The analysis of DEGs in 14 KEGG pathways annotated to lipid metabolism found 503 DEGs (Additional file 11: Table S8) belonging to 111 enzymes. Then, based on the standard FPKM and LOG2 values of different replicates, 111 DEGs were selected for heat map and cluster analysis (Additional file 12: Table S9 and Fig. 5). The results showed that 111 DEGs for lipid metabolism were clustered into four clusters (Fig. 5a). There were 11, 26, 32 and 42 DEGs in Cluster I, II, III and IV, respectively. The DEGs in Cluster I and II were highly expressed, whereas those in Cluster III and IV were expressed at a relatively low level. Additionally, the average log2 FPKM values for the 11 DEGs in Cluster I and the 42 DEGs in Cluster IV showed an increasing pattern; however, those of the 26 DEGs in Cluster II and 32 DEGs in Cluster III showed a declining pattern (Fig. 5b).

### Analysis of DEGs for fatty acid biosynthesis, elongation and desaturation

**Fig. 6:**
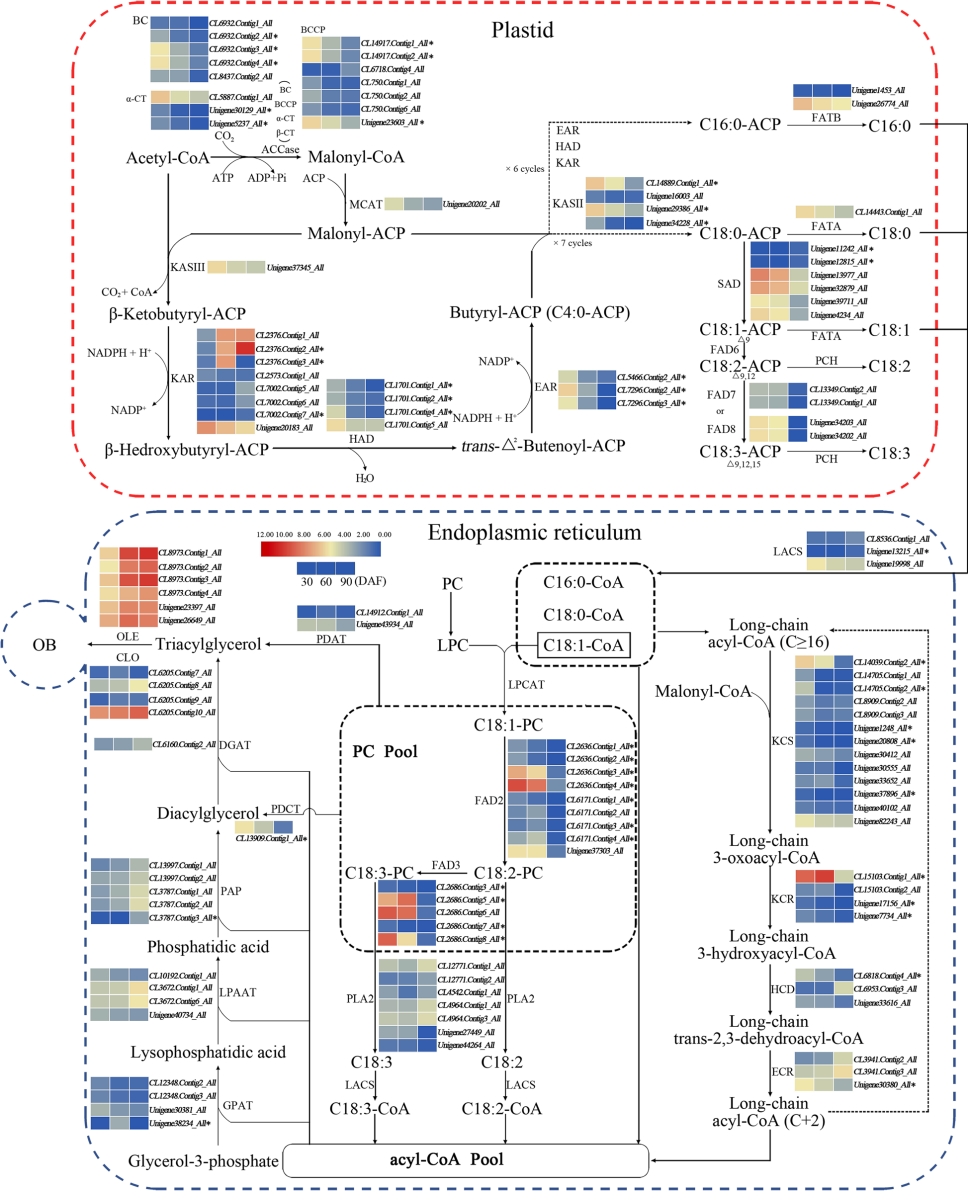
Proposed gene networks involved in unsaturated fatty acid biosynthesis and oil accumulation in *Paeonia lactiflora* ‘Hangshao’ seeds. The expression levels (represented by the Log_2_ FPKM) of the possible candidates are highlighted by color scales (blue to red scale) in *Paeonia lactiflora* ‘Hangshao’ seeds at different development stages (30, 60 and 90 DAF). BC: biotin carboxylase; BCCP: biotin carboxyl carrier protein; α-CT: carboxyl transferase subunit alpha; β-CT: carboxyl transferase subunit beta; MACT: malonyl-CoA-acyl carrier protein transacylase; KASIII: 3-oxoacyl-ACP synthase III; KAR: 3-oxoacyl-ACP reductase; HAD: 3-hydroxyacyl-ACP dehydratase; EAR: enoyl-ACP reductase I; KASII: 3-oxoacyl-ACP synthase II; FATB: fatty acyl-ACP thioesterase B; FATA: fatty acyl-ACP thioesterase A; SAD: stearoyl-ACP desaturase; FAD: fatty acid desaturase; PCH: palmitoyl-CoA hydrolase; LACS: long-chain acyl-CoA synthetase; PC: phosphatidylcholine LPC: lysophosphatidylcholine; LPCAT: lysophosphatidylcholine acyltransferase; PLA2: phospholipase A2; KCS: 3-ketoacyl-CoA synthase; KCR: very-long-chain 3-oxoacyl-CoA reductase; HCD: very-long-chain (3R)-3-hydroxyacyl-CoA dehydratase; ECR: very-long-chain enoyl-CoA reductase; GPAT: glycerol-3-phosphate acyltransferase; LPAAT: 1-acyl-sn-glycerol-3-phosphate acyltransferase; PAP: phosphatide phosphatase; DGAT: diacylglycerol O-acyltransferase; PDAT: phospholipid:diacylglycerol acyltransferase; PDCT: phosphatidylcholine:diacylglycerol cholinephosphotransferase; OB: oil body; OLE: oleosin; CLO: caleosin. * represented DEGs in all three groups. This model was developed based on the transcriptome data obtained in this study and information from Li et al. [17], Tian et al. [24] and Yang et al. [28]

To further uncover the key genes associated with the biosynthesis of unsaturated fatty acids and oil accumulation in the developmental herbaceous peony ‘Hangshao’ seed, DEGs in five pathways were analyzed, including fatty acid biosynthesis, fatty acid elongation, biosynthesis of unsaturated fatty acids, triacylglycerol (TAG) biosynthesis and lipid storage (Fig. 6). First, the DEGs with a log2 value of the FPKM ration in any two periods greater than 1 or less than − 1 were selected. Then, sequences of the DEGs selected above belonging to the same enzyme were compared using Bioxm software. Last, those DEGs with the same sequences were removed. Finally, we identified 123 DEGs involved in fatty acid biosynthesis (39 DEGs), fatty acid elongation (33 DEGs), biosynthesis of unsaturated fatty acids (24 DEGs), TAG biosynthesis (17 DEGs) and lipid storage (10 DEGs) (Additional file 13: Table S10 and Fig. 6). The expression levels of these DEGs at 30, 60 and 90 DAF are summarized in Additional file 13.

For fatty acid biosynthesis, the critical steps and key enzymes are shown in Fig. 6. First, acetyl-CoA conversion to malonyl-CoA is catalyzed by the enzyme acetyl-CoA carboxylase (ACCase), which consists of four subunits: biotin carboxylase (BC), carboxyl transferase subunit alpha (α-CT), carboxyl transferase subunit beta (β-CT) and biotin carboxyl carrier protein (BCCP). We identified 15 DEGs encoding ACCase subunits, including 5 *BC*, 3 *α-CT* and 7 *BCCP*. Then, malonyl-CoA was converted to malonyl-ACP, the primary substrate for a subsequent cycle of condensation reactions, by malonyl-CoA ACP transacylase (MCAT). Only 1 DEG was identified as *MCAT*. Subsequently, malonyl-ACP combined with acetyl-CoA were condensed to β-ketobutyryl-ACP by 3-oxoacyl-ACP synthase III (KASIII), and only 1 DEG for *KASIII* was identified. Then, β-ketobutyryl-ACP was converted to β-hedroxybutyryl-ACP via reduction by 3-oxoacyl-ACP reductase (KAR), and 8 DEGs were identified as *KAR*. Next, the β-hedroxybutyryl-ACP was dehydrated by 3-hydroxyacyl-ACP dehydratase (HAD) to generate *trans*-Δ^2^-butenoyl-ACP, and 4 DEGs were identified as *HAD*. The *trans*-Δ^2^-butenoyl-ACP was reduced to butyryl-ACP (C4:0-ACP) by enoyl-ACP reductase I (EAR), and 3 unigenes were identified as *EAR*. Subsequently, after 6 cycles for a series of condensation, reduction, dehydration and reduction reactions by 3-oxoacyl-ACP synthase II (KASII), KAR, HAD and EAR, C4:0-ACP was transformed to palmitoyl-ACP (C16:0-ACP). Moreover, after 7 cycles of those reactions, C4:0-ACP was transformed to stearoyl-ACP (C18:0-ACP). We identified 4 DEGs as *KASII*. Under the effect of fatty acyl-ACP thioesterase B (FATB) and fatty acyl-ACP thioesterase A (FATA), C16:0-ACP and C18:0-ACP were transformed to palmitic acid (C16:0, PA) and stearic acid (C18:0, SA), respectively. Two DEGs were identified as *FATB*, but only 1 DEG was identified as *FATA*.

Fatty acid elongation occurs via long-chain acyl-CoA (C≥16) in endoplasmic reticulum. Fatty acids (C≥16), which are catalyzed by thiolase from fatty acyl-ACP in plastids, are transformed to long-chain acyl-CoA (C≥16) by long-chain acyl-CoA synthetase (LACS) and transported to the endoplasmic reticulum. Three DEGs identified as *LACS*. After a series of complicated biochemical reactions, long-chain acyl-CoA (C≥16) was transformed to long-chain acyl-CoA (C+ 2) by 3-ketoacyl-CoA synthase (KCS), very-long-chain 3-oxoacyl-CoA reductase (KCR), very-long-chain (3R)-3-hydroxyacyl-CoA dehydratase (HCD) and very-long-chain enoyl-CoA reductase (ECR), and 13, 4, 3 and 3 DEGs were identified as *KCS*, *KCR*, *HCD* and *ECR*, respectively.

The biosynthesis of unsaturated fatty acids occurs via two pathways, the plastid pathway and endoplasmic reticulum pathway, beginning with C18:0-ACP. First, with dehydrogenation by stearoyl-ACP desaturase (SAD), C18:0-ACP is transformed to oleoyl-ACP (C18:1-ACP) in the plastid. Six DEGs were identified as *SAD*. Then, some C18:1-ACP was dehydrogenized to linoleoyl-ACP (C18:2-ACP) by delta-12 desaturase in the plastid (FAD6). No DEGs were identified as *FAD6*. Next, C18:2-ACP was dehydrogenized by Delta-15 desaturase in the plastid (FAD7/8) to form alpha-linolenoyl-ACP (C18:3-ACP). Two DEGs each were annotated to *FAD7* and *FAD8*. However, some C18:1-ACP was transformed to C18:1 by FATA, which was transformed to C18:1-CoA by LACS, and then C18:1-CoA was transformed in the endoplasmic reticulum. Subsequently, C18:1-CoA and lysophosphatidylcholine (LPC) were transformed to C18:1-PC by lysophosphatidylcholine acyltransferase (LPCAT). No DEGs were identified as *LPCAT*. Next, C18:1-PC was dehydrogenized to C18:2-PC by delta-12 desaturase in the endoplasmic reticulum (FAD2), and C18:2-PC was dehydrogenized to C18:3-PC by delta-15 desaturase in the endoplasmic reticulum (FAD3). Nine and 5 DEGs were identified as *FAD2* and *FAD3*, respectively.

### Analysis of DEGs for TAG assembly and oil accumulation

Most fatty acids in plants are stored in the form of TAG. The fatty acids synthesized in the plasmid are generally transferred from the plastid to the endoplasmic reticulum in the form of fatty acyl-CoA and become a substrate for TAG assembly. First, under catalysis by glycerol-3-phosphoate acyltransferase (GPAT), the fatty acid carbon chain on the fatty acyl-CoA molecule is transferred to the *sn-1* position of glycerol-3-phosphate (G3P) to form lysophosphatidic acid (LPA). Then, by catalysis of lyso-phosphatidic acid acyltransferase (LPAAT), phosphatidic acid (PHA) is formed from the *sn-2* position of LPA binding the fatty acid carbon chain of fatty acyl-CoA. Next, PHA is dephosphorylated by phosphatidic acid phosphatase (PAP) to form diacylglycerol (DAG). Finally, DAG is catalyzed by diacylglycerol acyltransferase (DGAT) to bind the fatty acid carbon chain of fatty acyl-CoA to form TAG at the *sn-3* position. We identified 4, 4, 5 and 1 DEGs as *GPAT*, *LPAAT*, *PAP* and *DGAT*, respectively.

TAG is stored as an oil body (OB), which is the main lipid-storage organelle in plant cells. Additionally, OB contains the hydrophobic core of TAG, which is surrounded by a monolayer of phospholipids and unique proteins called oleosin (OLE), caleosin (CLO) and steroleosin (STE), which play important roles in maintaining the stability of OB, regulating the size of OB, and controlling the amount of OB. We identified 6 and 4 DEGs as *OLE* and *CLO*, respectively. We also found that the expression levels of most of those DEGs showed a rising trend.

The heat maps of the abovementioned DEGs are shown in Fig. 6. We found that most of the DEGs had relatively low expression levels, which appear blue in the heat map, and a small number of them were highly expressed, which appear read in the heat map, including 4 *KAR*, 1 *KCR*, 2 *SAD*, 4 *FAD3*, 6 *OLE* and 1 *CLO*. In addition, we analyzed the number and attribution of DEGs in each group (Additional file 13: Table S10). In Group I, 76 DEGs, including 17 upregulated and 59 downregulated DEGs, were divided into 32 DEGs in the fatty acid biosynthesis pathway, 15 DEGs in the fatty acid elongation pathway, 13 DEGs in the biosynthesis of unsaturated fatty acid pathway, 8 DEGs in the TAG assembly pathway, and 8 DEGs in the lipid storage pathway. In Group II, 90 DEGs, including 28 upregulated and 62 downregulated DEGs, were divided into 26 DEGs in the fatty acid biosynthesis pathway, 26 DEGs in the fatty acid elongation pathway, 24 DEGs in the biosynthesis of unsaturated fatty acid pathway, 12 DEGs in the TAG assembly pathway, and 2 DEGs in the lipid storage pathway. In Group III, 118 DEGs, including 32 upregulated and 86 downregulated DEGs, were divided into 39 DEGs in the fatty acid biosynthesis pathway, 30 DEGs in the fatty acid elongation pathway, 24 DEGs in the biosynthesis of unsaturated fatty acid pathway, 16 DEGs in the TAG assembly pathway, and 9 DEGs in the lipid storage pathway. Moreover, we found 47 DEGs in all three groups (Fig. 6, marked as *), among which 33 DEGs were downregulated, including *BC* (3), *α-CT* (2), *BCCP* (3), *HAD* (3), *EAR* (3), *KASII* (3), *KCS* (3), *KCR* (2), *HCD* (1), *ECR* (1), *FAD2*(5), *FAD3*(3) and *PDCT* (1); only 1 DEG was upregulated belonging to *KAR*, and the other 13 DEGs were upregulated in one group or downregulated in another group.

### Characteristics of the *BCCP* gene family in *Paeonia lactiflora* ‘Hangshao’

**Fig. 7:**
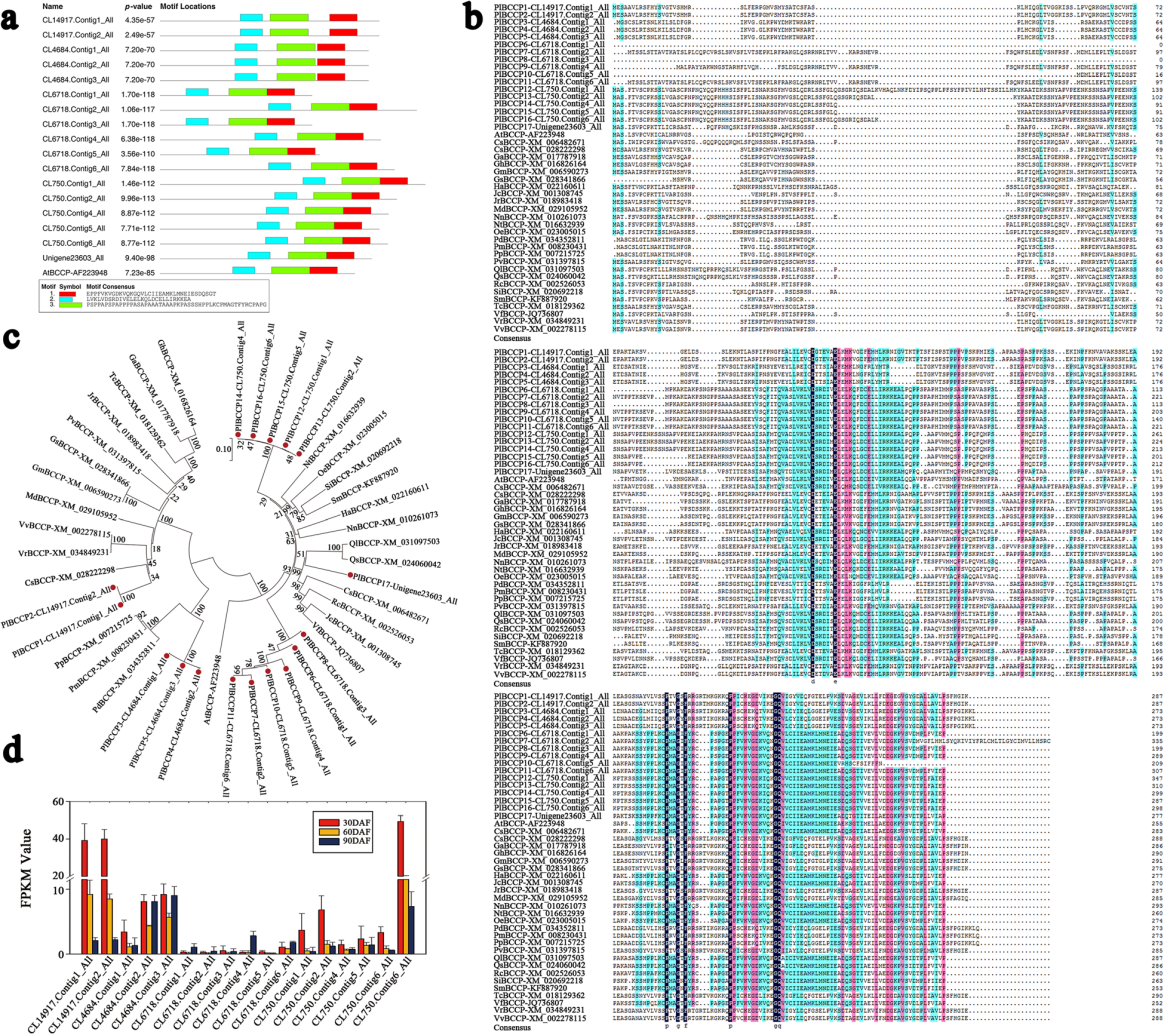
Comparative and phylogenetic analysis and FPKM value of 17 genes putatively encoding BCCP in *Paeonia lactiflora* ‘Hangshao’. **a** Protein domain analysis of 17 BCCP: MEME prediction resulet; **b** Amino acid sequence alignment of BCCP family in plant species; **c** Phylogenetic analysis of the BCCP proteins in *Paeonia lactiflora* ‘Hangshao’ and other plant speices. Other plant species including *Gossypium hirsutum* (Gh), *Gossypium arboreum* (Ga), *Theobroma cacao* (Tc), *Juglans regia* (Jr), *Pistacia vera* (Pv), *Glycine soja* (Gs), *Glycine max* (Gm), *Malus domestica* (Md), *Vitis vinifera* (Vv), *Vitis riparia* (Vr), *Camellia sinensis* (Cs-XM 02822298), *Prunus persica* (Pp), *Prunus mume* (Pm), *Prunus dulcis* (Pd), *Arabidopsis thaliana* (At), *Vernicia fordii* (Vf), *Jatropha curcas* (Jc), *Ricinus communis* (Rc), *Citrus sinensis* (Cs-XM 006482671), *Quercus suber* (Qs), *Quercus lobata* (Ql), *Nelumbo nucifera* (Nn), *Helianthus annuus* (Ha), *Salvia miltiorrhiza* (Sm), *Sesamum indicum* (Si), *Olea europaea* var. *sylvestris* (Oe) and *Nicotiana tabacum* (Nt). **d** FPKM value of 17 BCCP in *Paeonia lactiflora* ‘Hangshao’ from transcriptome database

We identified 17 *BCCP* genes in *Paeonia lactiflora* ‘Hangshao’ from transcriptome database, and the gene sequences were available in Additional file 14: Table S11. To further analyze the diversity of BCCP protein in *P. lactiflora* ‘Hangshao’, we used MEME to analyze the conservative motif. Generally speaking, proteins clustered in a family have similar motif composition. The results showed that these 17 BCCP proteins all contained three conserved motifs (Fig. 7a). Compared with BCCP protein in *Arabidopsis thaliana*, there are also three conservative motifs, but there are still differences in the motif locations among them. Subsequently, we used DNAMAN to compare the amino acid sequence of BCCP in *P. lactiflora* ‘Hangshao’ with the amino acid sequence of other plants in NCBI and found that they have certain similarities (Fig. 7b). Then, we constructed the phylogenetic tree with the neighbor-joining method using MEGA 7.0 following multiple alignments of protein sequences with ClustalW (Fig. 7c). The alignment results indicated that the BCCP protein sequence has a consistency in different plants. And all 17 BCCPs were divided into 5 clades. Moreover, we analyzed the FPKM value of 17 BCCP (Fig. 7d), and found that most of them are less than 10 except BCCP1 (CL14917.Contig1_All), BCCP2 (CL14917.Contig2_All) and BCCP17 (Unigene23603_All) in 30 DAF.

### Experimental validation and analysis of sixteen DEGs involved in lipid metabolism

**Fig. 8:**
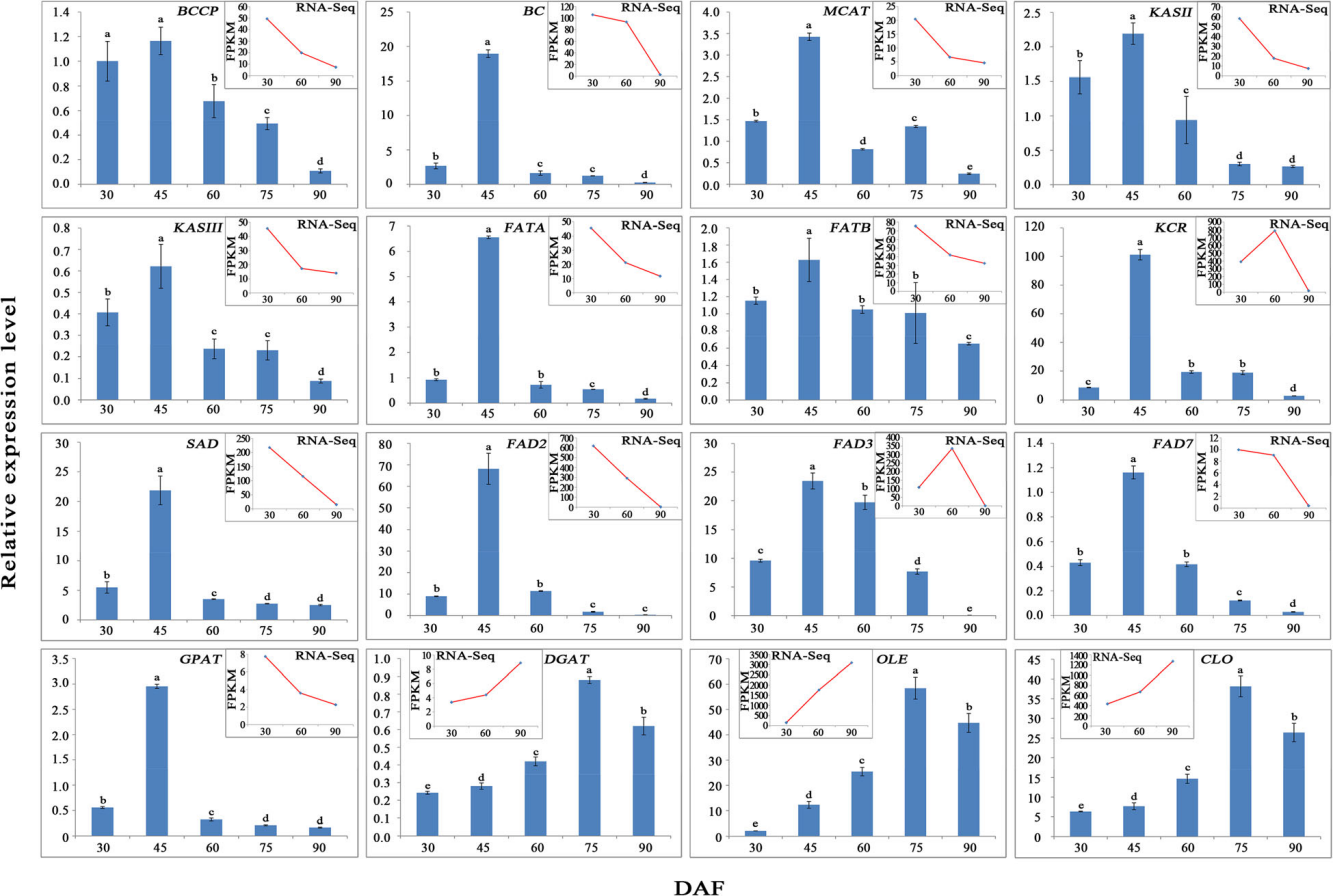
qRT-PCR validation of the expression levels of DEGs related to unsaturated fatty acid biosynthesis and oil accumulation for developmental seeds of *Paeonia lactiflora* ‘Hangshao’ The column chart shows the qRT-PCR validations in 5 periods; the broken line graph shows the transcriptome sequencing in 3 periods.

To validate the DEGs, the relative expression levels of 16 randomly selected DEGs involved in lipid metabolism of KEGG pathway were analyzed by qRT-PCR. Among them, there were 7 DEGs related to fatty acid biosynthesis, namely, *BCCP*, *BC*, *MCAT*, *KASII*, *KASIII*, *FATA* and *FATB*, and 1 DEG was involved in fatty acid elongation (*KCR*). Four DEGs were involved in the biosynthesis of unsaturated fatty acids, including *SAD*, *FAD2*, *FAD3* and *FAD7*, and 2 DEGs, *GPAT* and *DGAT*, were involved in TAG assembly and 2 DEGs, *OLE* and *CLO*, in lipid storage (Fig. 8). Overall, the expression patterns of 16 DEGs verified by qRT-PCR were highly consistent with the transcriptome sequencing results, indicating that the DEG analysis findings were reliable.

## Discussion

Most plants contain accumulated oil (mainly TAG) in their seeds to provide energy for seed germination. Plant oils have great significance in agricultural production, and most are mainly used as edible oil in food processing and preparation [29]. Plant oils contain essential fatty acids, including omega-3 series ALA and omega-6 series LA, which can only be synthesized in plants [30]. The proportion of fatty acids in plant oils, especially the proportion of unsaturated fatty acids, is one of the evaluation criteria for plant oil quality [31]. However, the content of LA and ALA constituting different proportions of essential fatty acids have different effects on human physiological functions [32, 33]. At present, many studies have shown that the intake ratio of LA/ALA has the most significant physiological function at 1:1 [34]. In this study, the oil content of mature seed of herbaceous peony ‘Hangshao’ was approximately 20%, and five main fatty acids including PA, SA, OA, LA and ALA were detected by GC-MS, which is consistent with the findings for other peonies [11, 17]. The absolute contents of the five fatty acid components during seed development were all highest for ALA and lowest for SA. The specific manifestations followed the order ALA>LA>OA>PA>SA. The ratio of LA/ALA was lower during the development of seeds of herbaceous peony ‘Hangshao’, maintained between 0.55 and 0.71, which is close to the optimal intake ratio of 1:1. Therefore, herbaceous peony seed oil has potential to become a new resource of nutrition for specific populations.

Oilseed plants have high contents of seed oil compared with other plants. Despite the similarities of lipid biosynthesis and its components in oilseed plants, the contents of oil and fatty acid vary considerably. However, the regulation of oil content and fatty acid composition is a complex process, which varies in oil biosynthesis phases and accumulation. Nevertheless, the difference in regulatory mechanisms in different species is attributed to the variation in oil content among species [35]. Mainly, the molecular regulators of oil content and fatty acid composition consist of genes involved in fatty acid and TAG assembly [36]. Therefore, the transcriptome was used to understand the genes, gene networks, regulatory factors and their interactions that direct seed oil biosynthesis [37]. In this study, a comparative transcriptome was used for the first time to reveal the high oil content and unsaturated fatty acids of the herbaceous peony ‘Hangshao’ seed. Here we de novo assembled the transcript in developing seeds of *P. lactiflora* ‘Hangshao’, resulting in 150,156 unigenes, exceeding the transcriptome data from other oilseed plants such as *Malania oleifera* [28], *Camellia oleifer* [23] and *Paeonia ostii* [38]. We think that the number of unigenes may have a great divergence in different plants. Even in the same plant, the number of unigenes may be different in various tissues and/or at different stages of development. For example, the unigenes in the buds, stems and budding seeds of *P. lactiflora* were 207,827 [39], 107,929 [40] and 123,577 [41], respectively. Of course, it is undeniable that there may be incomplete and redundant stitching in Trinity based on sequencing by using Illumina HiSeq 4000 system. When the length of one gene is relatively long, it is difficult to assemble the full length of the gene, and it is possible that some unigenes actually belong to the part of one gene. In order to obtain as complete unigenes as possible, we took a series of measures. Firstly, we repeated it three times at three different stages to construct the cDNA libraries and sequenced using the Illumina Hiseq 4000 platform. Secondly, sequence adapters, reads with more than 5% N base and low-quality reads (percentage of base with a quality less than 15 greater than 20% in a read) were removed to obtain clean reads. Lastly, all clean reads were assembled using Trinity software [42], and Tgicl software [43] was used to perform clustering and eliminate redundant data in the assembled transcipts to obtain unique genes. The clustering quality metrics are shown as Additional file Table S2. Moreover, we selected 16 differentially expressed genes (DEGs) to analyze the relative expression by qRT-PCR to validate the transcriptome sequencing results. Overall, we found that the expression patterns of 16 DEGs verified by qRT-PCR were highly consistent with the transcriptome sequencing results, indicating that the DEG analysis findings were reliable (Fig. 8).

One of main objectives of this study was to identify potential unigenes involved in unsaturated fatty acid biosynthesis and oil accumulation in developing seeds of herbaceous peony. However, oil accumulation mainly involves fatty acid biosynthesis (including fatty acid carbon chain elongation and desaturation), TAG assembly and lipid storage [44]. Overall, to produce a C16 or C18 fatty acid from acetyl-CoA and malonyl-CoA, more than 30 enzymatic reactions are required, involving enzymes such as ACCase (including BC, BCCP, α-CT and β-CT), MCAT, KAS (including KASI, KASII and KASIII), KAR, HAD, EAR, and FAT (including FATA and FATB), among others [45]. In this study, we screened out many relevant DEGs encoding the above enzymes (Fig. 6). Most of them were downregulated DEGs such as *BCCP*. Even though some of these DEGs were upregulated, their FPKM values were lower than 10, except for two DEGs that were annotated to *KAR* (named CL2376.Contig1_All and CL2376.Contig2_All). Interestingly, only 1 unigene each was annotated to MCAT, KASIII and FATA, all of which were downregulated DEGs. MCAT is a key enzyme in the fatty acid synthesis pathway, and its expression level is closely related to lipid accumulation. At present, overexpression of the *MCAT* gene in rape and other plants has a positive effect on promoting the accumulation of C16 and C18 fatty acids [46]. KASIII is a key enzyme that initiates de novo fatty acid synthesis. At present, *KASIII* has been cloned from plants such as sunflower [47] and jatropha [48]. Some studies have shown that KASIII can use other receptor substrates to generate different final products of fatty acid synthesis, which indicates that KASIII is one of the main factors determining the carbon chain structure of fatty acids [49, 50]. In addition, KASIII, as the initiator of de novo synthesis of fatty acid, plays a second role in determining the length of the carbon chain of fatty acids [51]. Some studies have suggested that the synthesis rate of fatty acid is affected by KASIII enzymatic activity, indicating that it may be one of the rate-limiting enzymes in the fatty acid synthesis pathway [52]. In the present study, the FPKM value of the *KASIII* gene in this transcriptome decreased with the development of seeds, but its expression by qRT-PCR was highest at 45 DAF (Fig. 8), which was consistent with the accumulation rate of fatty acid in herbaceous peony seeds. FATA is a type of acyl-ACP thioesterase (FAT). De novo synthesis of fatty acid was terminated by FAT, which hydrolyzes the thiol-lipid bond located between acyl and ACP to form fatty acids [53]. The specificity of FAT largely determines the chain length and unsaturated degree of plant fatty acids in glycerolipid and TAG [54]. According to the sequence alignment, FAT can be classified as FATA and FATB, which have different substrate specificities. However, FATA has the highest catalytic activity of C18:1^Δ9^-ACP [55]. In this study, the FPKM value of *FATA* in this transcriptome also decreased with the development of seeds; however, the tendency of *FATA* expression by qRT-PCR was consistent with the total accumulation rate of OA, LA and ALA in herbaceous peony seeds.

UFAs are the main components of plant cell membrane lipids and storage lipids, which play important roles in physiological functions. In most plants, UFAs account for more than 75% of the total fatty acids [45]. In this study, there were more than 95% UFAs, especially 70% PUFAs in herbaceous peony seed oil (Fig. 1c). The first double bond is introduced by the soluble enzyme stearoyl-ACP desaturase (SAD), which catalyzes the desaturation of C18:0-ACP to form C18:1-ACP. There are subsequently two pathways of desaturation by fatty acid desaturases (FADs). In one pathway, C18:1-ACP is successively transformed to C18:2-ACP and C18:3-ACP in plastid by FAD6 and FAD7 or FAD8, respectively. In another pathway, C18:1-ACP undergoes a series of enzymatic reactions to produce C18:1-PC, which is desaturated by FAD2 and FAD3 in the endoplasmic reticulum to form C18:2-PC and C-18:3 PC. SAD controls the total amount of UFAs in plant oils and the ratio of saturated fatty acid to UFA [56]. The content of SA in the *SAD* mutant was found to increase from 0.8 to 14.3% compared with wild-type *Arabidopsis*, accompanied by a decrease in OA content [57]. In contrast, upon overexpression of *SAD* in *Arabidopsis*, the SA content decreased and OA content increased [58]. The expression of *SAD* was higher in seeds than in other tissues, and the expression of *SAD* was bell-shaped with seed development [59]. FADs are all intact membrane proteins, including FAD2, FAD3, FAD6, FAD7 and FAD8, and they catalyze the desaturation of C18 fatty acids. FAD2/FAD6 catalyze the dehydrogenation of OA at the Δ12 position to form LA [60], and LA to form ALA by Δ15 fatty acid dehydrogenase including FAD3, FAD7 and FAD8 [61]. In this process, FADs play a key role in the formation of ALA [62]. FAD2 is a key enzyme that controls the transformation of OA to LA. By inducing the mutation of *FAD2* or inhibiting its expression by antisense methodologies, the content of OA in soybean [63], sunflower [64] and peanut [65] seed oil can be improved, thus improving the quality of edible oil. The *FAD3* gene is semidominant, and its overexpression leads to a decrease in LA and an increase in ALA [66, 67]. In this study, we identified 6, 9, 5, 2 and 2 DEGs annotated to *SAD*, *FAD2*, *FAD3*, *FAD7* and *FAD8*, respectively. The FPKM values of those DEGs at 90 DAF were mostly less than 1, which indicated that the absolute contents of unsaturated fatty acid in herbaceous peony seed oil showed a downward trend at the later stage. Although most of the FPKM values of *SAD*, *FAD2* and *FAD3* based on the transcriptome sequencing showed a decreasing trend with seed development, their expression levels were highest at 45 DAF by qRT-PCR analysis and exhibited a bell shape with seed development (Fig. 8), consistent with the rate of unsaturated fatty acid accumulation (Fig. 1c). Moreover, the FPKM values of *FAD7* and *FAD8* were more lower than that of *FAD3*. Therefore, we inferred that the key genes in herbaceous peony seed oil with high contents of unsaturated fatty acids, such as ALA and LA, are *SAD*, *FAD2* and *FAD3*.

Most fatty acids in plants are successively transferred to the *sn-1*, *sn-2* and *sn-3* position of glycerol-3-phosphate to form TAG, which is assembled in the endoplasmic reticulum through the Kennedy pathway, and finally formed into an oil body by budding for storage [68]. TAG assembly mainly involves enzymes including GPAT, LPAAT, PAP and DGAT [69]. DGAT catalyzes the final step of TAG assembly in the Kennedy pathway, and it is the only rate-limiting enzyme in this pathway [70]. The expression of single *DGAT* can restore the accumulation of TAG and the formation of OBs [71]. Four different types of *DGATs* were found in plants, namely, *DGAT1*, *DGAT2*, *DGAT3* and *WS/DGAT* [72, 73]. Organ expression of *DGAT* is specific. *DGAT1* is highly expressed in developing seeds, petals, and flower buds of most dicotyledonous plants, but it is poorly expressed in leaves and stems [74, 75]. In this study, there was only 1 DEG annotated to *DGAT*, the FPKM value of which showed a continuous upward trend and expression by qRT-PCR was highest at 75 DAF, which was consistent with the highest oil content at 75 DAF. TAGs are the most abundant neutral lipids, and most TAGs are partitioned in small spherical particles called oil bodies (OBs) [68]. OBs contain a hydrophobic core of TAG surrounded by a monolayer of phospholipids and unique proteins called oleosin (OLE), caleosin and steroleosin [76]. Among these proteins, OLE is the main protein and covers almost the whole surface of OBs, accounting for approximately 75–80% [77]. OLE plays an important role in maintaining the size and stability of the oil body [78]. In addition, Siloto et al. found that the expression level of *OLE* in *Arabidopsis* seeds with high oil content was higher than that in seeds with low oil content [79]. The expression of *OLE* is higher in seeds than in roots, stems, leaves and flowers. Additionally, with the development of seeds, the expression of *OLE* in seeds first increases and then decreases [80]. In the present study, we identified 6 DEGs annotated to *OLE*, the FPKM values of which in the transcriptome were very high and increasing; however, the expression of *OLE* by qRT-PCR was highest at 75 DAF, which was consistent with the oil accumulation. Similarly, the trend of *OLE* expression in developmental seeds was consistent with other studies. Therefore, we speculated that high oil accumulation in herbaceous peony seed was related to the high expression levels of *DGAT* and *OLE*.

BCCP, or biotin carboxyl carrier protein, is one of the subunits of ACCase, and BC, α-CT and β-CT constitute four subunits of ACCase which catalyze acetyl-CoA to malonyl-CoA that was the committed step for de novo fatty acid biosynthesis [81]. As an important component of ACCase, BCCP protein is the link and bridge connecting the other three subunits of ACCase, and plays a very important role in the biosynthesis of fatty acid from seeds [82]. Studies have shown that the up-regulation of BCCP protein expression is positively correlated with the specific activity of ACCase and the content of seed oil during the development of *Arabidopsis thaliana* and rapeseed seeds [82]. We identified 17 unigenes annotated to BCCP from transcriptome database of *P. lactiflora* ‘Hangshao’, and we found that the sequence conservation of BCCP was highly conserved by alignment with the amino acid sequences of family genes of various plants. According to the phylogenetic analysis, 17 BCCPs in *P. lactiflora* ‘Hangshao’ were divided into five clades. Chen et al. found that BCCPs was in four subfamilies, separated by more subtle but statistically significant differences in primary structure [83]. Moreover, Thelen et al. believed that *BCCP* gene can encode two kinds of BCCP protein, one can be biotinylated, and the other cannot be biotinylated [84]. These two kinds of BCCP proteins can regulate the activity of ACCase by competitive binding to the other subunits of ACCase, so as to realize the regulation of fatty acid synthesis process [84]. From these studies, it has been shown that BCCP has a significant influence on the content of fatty acid of plants.

## Conclusions

GC-MS analysis revealed that ‘Hangshao’ seeds have five main fatty acids, including palmitic acid (PA), stearic acid (SA), oleic acid (OA), linoleic acid (LA) and α-linolenic acid (ALA). The total content of the five fatty acids first increased and then decreased, reaching a maximum at 75 DAF. Additionally, the ratio of the content of OA to ALA was close to 1:1. A total of 150,156 unigenes were obtained by transcriptome sequencing, with an average length of 1030 bp. There were 15,005 unigenes annotated in the seven functional databases, including NR, NT, GO, KOG, KEGG, Swiss-Prot and InterPro. Based on the KEGG database, there were 1766 unigenes annotated to lipid metabolism. We also screened out 503 DEGs in lipid metabolism, which were annotated to 111 enzymes. To further uncover the key genes associated with the biosynthesis of unsaturated fatty acid and oil accumulation, we identified 123 DEGs involved in fatty acid biosynthesis (39 DEGs), fatty acid elongation (33 DEGs), biosynthesis of unsaturated fatty acid (24 DEGs), TAG assembly (17 DEGs) and lipid storage (10 DEGs). Furthermore, qRT-PCR was used to analyze the expression patterns of 16 related genes, including *BBCP*, *BC*, *MCAT*, *KASII, KASIII, FATA FATB*, *KCR*, *SAD, FAD2*, *FAD3*, *FAD7*, *GPAT, DGAT, OLE* and *CLO,* and the results showed that they were highly consistent with the transcriptome results. Moreover, we deduced that the key genes with high contents of unsaturated fatty acids and oil accumulation in herbaceous peony seed oil are *MCAT*, *KASIII*, *FATA*, *SAD*, *FAD2*, *FAD3*, *DGAT* and *OLE*.

In conclusion, we present the first high-quality transcriptome sequence for herbaceous peony seeds. The sequences of genes related to unsaturated fatty acid biosynthesis and oil accumulation were obtained based on large-scale transcriptome data, which will enable further metabolomic and gene functional studies. This species with rich PUFA content, accompanied by an omega-6/omega-3 ratio close to 1:1, was studied to form a more diversified set of PUFAs to eventually increase storage PUFA production and meet the needs of more humans worldwide for intake of an omega-6/omega-3 ratio close to 1:1.

## Methods

### Plant materials

Seeds of herbaceous peony ‘Hangshao’ were collected from the same plant which was introduced from Pan’an County Zhejiang Province (traditional Chinese medicine ‘Radix Paeoniae Alba’ production base), identified by Yang-Qian Cao, a senior agronomist from Heze Peony Research Institure in Shangdong Province of China, and cultivated in the peony germplasm resource garden of Yangzhou University, China (32°39′N, 119°42′E) for 8 years. Due to the limited fruit and low oil extraction rate at the early stage, the follicle was hand-collected in the 30 days after flowering (DAF) for the first time and at intervals of 15 days until full maturity (including 30, 45, 60, 75 and 90 DAF) for fatty acid absolute content analysis by GC-MS (Fig. 1a). Seeds used for transcriptome sequencing were collected from the same strain at developmental stages of 30, 60 and 90 DAF. The collected samples were immediately frozen in liquid nitrogen and stored in a − 80 °C cryogenic refrigerator until further use.

### Oil content analysis

Ten grams of powdered seeds were weighed and transferred to a 500-mL round-bottom flask. Then, 130 ml n-hexane at a 1:13 ratio (g·mL^− 1^) was added to the flask. The flask was connected to a condenser tube and placed in the ultrasonic cleaning machine (KQ-200VDB, Kunshan ultrasonic instrument co., LTD, China) to perform oil extraction at 70 °C and 200 W at a fixed frequency of 80 KHz. After extraction for 1.5 h, the mixture was centrifuged at room temperature for 10 min at 4000 r·min^− 1^. The supernatant was separated and dried under the rotary evaporator (RE52–99, Yarong biochemical instrument factory, Shanghai, China) at 55 °C for oil separation and n-hexane recovery. The oil was then stored in a refrigerator (4 °C) until fatty acid analysis. The oil content was calculated and expressed as % (oil weight/seed weight).

### Quantitative analysis of the main fatty acid composition

The absolute content of fatty acid was determined according to the national standard (GB 5009.168–2016) of the People’s Republic of China. The specific process is as follows.

Standard solution preparation: 1) 5 mg·mL^− 1^ triundecanoin (C11) internal standard solution: 0.5 g triundecanoin (C11) was added to a beaker and dissolved by the addition of methanol. Then, the solution was transferred into a volumetric flask and increased to a 100-mL constant volume with methanol. 2) Mixed standard solution of 37-component fatty acid methyl: 100 μL of the 37-component fatty acid methyl mixture was added to a 10-mL volumetric flask and increased to a constant volume with n-hexane. The two standard solutions were stored in a − 4 °C refrigerator.

Sample hydrolysis: A total of 0.5 g of seeds were ground into granules and placed in a flask. Then, 100 μL triundecanoin (C11) internal standard solution, 100 mg pyrogallic acid, several zeolites and 2 mL 95% ethanol were added and mixed. Subsequently, 10 mL hydrochloric acid solution was added and mixed well. Next, the flask was placed in a 70 °C water bath for hydrolysis for 40 min, after which it was cooled to room temperature.

Fat extraction: The hydrolyzed sample was supplemented with 10 mL 95% ethanol and mixed. The hydrolysate in the flask was then transferred to the separation funnel. Then, the flask and stopper were washed with 50 mL of the ethyl ether petroleum ether mixture. Next, the rinse solution was incorporated into the separation funnel, capped, shaken for 5 min, and set aside for 10 min. Subsequently, the extraction solution was collected into a 250-mL flask. The hydrolysate was extracted 3 times as described in the previous steps. Finally, the separation funnel was flushed with the petroleum ether with ether mixture (volume ratio 1:1), and the flushing fluid was collected into a constant weight flash that would be boiled in a water bath and dried at 100 °C for 2 h.

Fatty acid esterification: The 2 mL of 2% NaOH methanol solution was added to the fat extract and heated at 80 °C for 30 min. Then, 3 mL 14% trifluoro (methanol) boron solution was added and heated at 80 °C for 30 min. Next, the mixture was cooled to room temperature and placed in a centrifuge tube, and 1 mL n-hexane was added. It was extracted by shock for 2 min and then left for 1 h and stratified. One hundred microliters of the supernatant was increased to 1 mL with n-hexane and filtered with a 0.45-μm organic-phase filter into a 2-mL chromatographic sample bottle.

Chromatographic and mass spectrometric conditions: The conditions have been previously described [10]. The trinonadecanoin internal standard solution, mixed standard solution of 37-component fatty acid methyl and sample solution were successively subjected to GC-MS to determine the chromatographic peak area of each fatty acid response.

Calculation formula for the fatty acid absolute content: W_i_ (g·100 g^− 1^) =F_i_×(A_i_/A_C11_)×(ρ_C11_×V_C11_× 1.0067/M)× 100×N_i_, where W_i_ is the absolute content of i fatty acid, F_i_ is the response factor of fatty acid methyl ester, A_i_ is the peak area of fatty acid methyl ester in the sample, A_C11_ is the peak area of the triundecanoin (C11) internal standard solution added to the sample, ρ_C11_ is the concentration of triundecanoin (C11), which was 5 mg·mL^− 1^, V_C11_ is the volume of triundecanoin (C11), which was 0.1 mL, 1.0067 is the conversion coefficient of triundecanoin to methyl undecanoate, M is the sample mass (mg), 100 is the coefficient that converts the content to the content per 100 g of sample and N_i_ is the conversion coefficient of fatty acid methyl ester to fatty acid. The calculation formula of F_i_ is (ρ_Si_×A_11_)/(ρ_11_×A_Si_), ρ_Si_ is the concentration of fatty acid methyl ester in the 37-component fatty acid methyl mixture, ρ_11_ is the concentration of methyl undecanoate in the 37-component fatty acid methyl mixture, A_11_ is the peak area of methyl undecanoate and A_Si_ is the peak area of fatty acid methyl ester.

### Total RNA extraction, cDNA library preparation and transcriptome sequencing analysis

Nine seed samples from three stages (30, 60 and 90 DAF, with three biological replicates) were snap-frozen in liquid nitrogen and ground to a fine powder. Total RNA samples were extracted using the Total RNA Isolation System (Takara, Japan) according to the manufacturer’s instructions. The quality of the resulting RNA was verified by agarose gel electrophoresis using a Nanodrop ND-4301000 spectrophotometer and Agilent 2100 Bioanalyzer (Agilent, Santa Clara, CA, USA). After RNA detection, Oligo (dT) was used to isolate mRNA. Mixed with the fragmentation buffer, the mRNA samples were fragmented. Then cDNA was synthesized using the mRNA fragments as templates. Short fragments are purified and resolved with EB buffer for end reparation and single nucleotide A (adenine) addition. After that, the short fragments are connected with adapters and the suitable fragments are selected for the PCR amplification to construct the sample cDNA libraries. The quantification and qualification of the sample cDNA libraries were checked with Agilent 2100 Bioanaylzer and ABI StepOnePlus Real-Time PCR System during the QC steps. Finally, the nine qualified cDNA libraries (HS30d_1, HS30d_2, HS30d_3, HS60d_1, HS60d_2, HS60d_3, HS90d_1, HS90d_2 and HS30d_3) were sequenced using Illumina HiSeq 4000 system at Beijing Genomics Institute Co., Ltd. (Shenzhen, China). Raw data was analyzed through Sickle (https://github.com/najoshi/sickle) and SeqPrep (https://github.com/jstjohn/Seq-Prep), low-quality reads (percentage of base with a quality less than 15 greater than 20% in a read), adapter-containing reads and poly-N reads (with more than 5% N base) were removed to get high-quality clean reads. Based on the clean reads, the sequence duplication level and Q20, Q30, GC-content were calculated. Trinity software was used to assemble all of the clean reads from the left.fq and right.fq data, the min_kmer_cov was set as 2 and other parameters was set as their default. And Tgicl software [43] was used to perform clustering and eliminate redundant data in the assembled transcipts to obtain unique genes based on the parameters as followed: − 140 -c -v25 -O′-repeat_stringency 0.95 -minmatch 35 -minscore 35′. All clean reads were uploaded to the National Center for Biotechnology Information (NCBI) Sequence Read Archive (SRA) database under accession number SRP148668.

### Functional annotation and classification

To obtain comprehensive functional information, all Illumina-assembled unigenes were aligned against the NCBI nonredundant protein (Nr) (http://www.ncbi.nlm.nih.gov), NCBI nonredundant nucleotide sequence (Nt), cluster of euKaryotic Orthologous Groups (KOG) (http://www.ncbi.nlm.nih.gov/KOG), Kyoto Encyclopedia of Genes and Genomes (KEGG) (http://www.genome.jp/kegg) and Swiss-Prot protein (http://ftp.ebi.ac.uk/pub/databases/swissprot) databases using BLASTx alignments with an *E*-value cutoff of 10^− 5^ [85]. With Nr annotation, Gene Ontology (GO) annotation of unigenes was obtained using Blast2GO software (http://www.geneontology.org) [86]. Inter-Pro annotation was obtained using InterProScan5 [87].

### Analysis of differentially expressed genes (DEGs)

Before performing differential expression analysis of unigenes, we mapped all the clean reads of each sample to the unigenes with Bowtie2 [88], and we calculated the gene expression level with RSEM software based on the assembly results [89]. The FPKM (expected number of fragments per kilobase of transcript sequence per million mapped fragments) value was used to quantify gene expression levels [86]. All expression data sets and assembled unigenes are available at the NCBI Gene Expression Omnibus (GEO) database under accession number GSE151746. Next, we conducted a differential expression analysis of the samples with three biological replications using DESeq2 as described by Michaekl et al. [90]. Genes with a fold change ≥ 2.0 and adjusted *P*-value ≤ 0.05 were regarded as differentially expressed. DEG functions were explored through GO and KEGG pathway analysis, and a false discovery rate (FDR) no larger than 0.01 was defined as significant enrichment. This process was performed to identify significantly enriched metabolic pathways.

### Analysis of the BCCP gene family characteristics in *Paeonia lactiflora* ‘Hangshao’

To analyze *PlBCCP* family characteristics, we utilize BLASTp from NCBI non-redundant protein database to obtain its homologous genes. The conserved motifs of BCCP sequences were identified by MEME program (http://alternate.memesuite.org/tools/mem), parameters were set as maximum of 10 misifits and an optimum motif width of 6–200 amino acid residues [91]. ClustalX was used to align all of full-length BCCP protein sequences. To construct the phylogenetic tree, MEGA7.0 software was introduced with neighbor-joining (NJ) method, bootstrap values was set as 1000 replicates.

### Gene expression analysis using quantitative real-time PCR (qRT-PCR)

qRT-PCR was introduced to analyze gene transcript levels with BIO-RAD CFX Connect™ Optics Module (Bio-Rad, USA). Extracted seeds RNA (1 μg) were reverse-transcribed into cDNA in a 10-μl reaction based on the superscript first-strand synthesis system (PrimeScript® RT Reagent Kit With gDNA Eraser, TaKaRa, Japan). *PlActin* (JN105299) was used as an internal reference gene [92]. All specific primers for qRT-PCR were synthesized by Shanghai Sangon Biological Engineering Technology and Services Co., Ltd., sequences were shown in Additional file 15: Table S12. SYBR® Premix Ex Taq™ (Perfect Real Time) was used for qRT-PCR (TaKaRa, Japan). The PCR cycles were as follows: 55 °C for 2 min, followed by an initial denaturation step at 95 °C for 30 s, 40 cycles at 95 °C for 5 s, 55 °C for 15 s, and 72 °C for 30 s. 2^-ΔΔCt^ comparative threshold cycle (Ct) method was used for calculation of relative expression levels [93].

### Statistical analysis

Experiments described in this study were repeated three times through completely randomized design. Variance analysis was using SAS/STAT statistical analysis software (SAS Institute, Cary, NC, USA). Data shown in figures were means ± SDs (Standard deviation), and the different letters represent for significant differences (*P* < 0.05).

## Supplementary Information

**Additional file 1: Table S1**. Clean reads quality metrics

**Additional file 2: Table S2**. Quality metrics of transcripts of seeds of *Paeonia lactiflora* ‘Hangshao’

**Additional file 3: Figure S1**. The length distribution of unigenes for seeds of *Paeonia lactiflora* ‘Hangshao’

**Additional file 4: Figure S2**. Functional distribution of unigenes annotated for seeds of *Paeonia lactiflora* ‘Hangshao’

**Additional file 5: Table S3**. Number of DEGs for GO classification

**Additional file 6: Figure S3**. Functional distribution of GO-annotated DEGs for seeds of *Paeonia lactiflora* ‘Hangshao’

**Additional file 7: Table S4**. Number of DEGs for KEGG annotation

**Additional file 8: Table S5**. DEGs for KEGG annotation in Groups I, II and III

**Additional file 9: Table S6**. Number of DEGs for KEGG pathway annotation in Groups I, II and III

**Additional file 10: Table S7**. Number of DEGs in 14 KEGG lipid metabolism pathways

**Additional file 11: Table S8**. All DEGs annotated to lipid metabolism

**Additional file 12: Table S9**. One hundred eleven DEGs used for the cluster analysis

**Additional file 13: Table S10**. DEGs related to fatty acid biosynthesis and oil accumulation in herbaceous peony ‘Hangshao’ seeds

**Additional file 14: Table S11**. Gene sequences of 17 BCCP in herbaceous peony ‘Hangshao’ from tanscriptome database

**Additional file 15: Table S12**. Gene-specific primer sequence for qRT-PCR detection

## Data Availability

All data generated or analyzed during this study are included in the main paper and supplementary information files. In addition, all clean reads were uploaded to the National Center for Biotechnology Information (NCBI) Sequence Read Archive (SRA) database under accession number SRP148668. And all expression data sets and assembled unigenes are available at the NCBI Gene Expression Omnibus (GEO) database under accession number GSE151746.

## Abbreviations

DEG: Differentially expressed gene
UFA: Unsaturated fatty acid
OA: Oleic acid
LA: Linoleic acid
ALA: α-linolenic acid
PA: Palmitic acid
SA: Stearic acid
MUFA: Monounsaturated fatty acid
PUFA: Polyunsaturated fatty acid
DAF: Days after flowering
qRT-PCR: Quantitative real-time PCR
GC-MS: Gas chromatography-mass spectrometry
GO: Gene ontology
KOG: Clusters of orthologous groups
KEGG: Kyoto encyclopedia of genes and genomes
BC: Biotin carboxylase
BCCP: Biotin carboxyl carrier protein
α-CT: Carboxyl transferase subunit alpha
β-CT: Carboxyl transferase subunit beta
MACT: Malonyl-CoA-acyl carrier protein transacylase
KASIII: 3-oxoacyl-ACP synthase III
KAR: 3-oxoacyl-ACP reductase
HAD: 3-hydroxyacyl-ACP dehydratase
EAR: Enoyl-ACP reductase I
KASII: 3-oxoacyl-ACP synthase II
FATB: Fatty acyl-ACP thioesterase B
FATA: Fatty acyl-ACP thioesterase A
LACS: Long-chain acyl-CoA synthetase
KCS: 3-ketoacyl-CoA synthase
KCR: Very-long-chain 3-oxoacyl-CoA reductase
HCD: Very-long-chain (3R)-3-hydroxyacyl-CoA dehydratase
ECR: Very-long-chain enoyl-CoA reductase
SAD: Stearoyl-ACP desaturase
FAD: Fatty acid desaturase
LPC: Lysophosphatidylcholine
LPCAT: Lysophosphatidylcholine acyltransferase
GPAT: Glycerol-3-phosphate acyltransferase
LPAAT: 1-acyl-sn-glycerol-3-phosphate acyltransferase
PAP: Phosphatide phosphatase
DGAT: Diacylglycerol O-acyltransferase
PDAT: Phospholipid:diacylglycerol acyltransferase
PDCT: Phosphatidylcholine: diacylglycerol cholinephosphotransferase
TAG: Triacylglycerol
OLE: Oleosin
CLO: Caleosin
FPKM: Fragments per kilobase of exon model per million mapped fragment
OB: Oil body
