# Supplementary material for: Identification of genes associated with the biosynthesis of unsaturated fatty acid and oil accumulation in herbaceous peony ‘Hangshao’ (Paeonia lactiflora ‘Hangshao’) seeds based on transcriptome analysis
Source: BMC Genomics. 2021 Feb 1;22:94. doi: 10.1186/s12864-020-07339-7 (PMC7849092; doi:10.1186/s12864-020-07339-7)
Supplement: Supplementary file 1 — Additional file 1: Table S1. Clean reads quality metrics [file 12864_2020_7339_MOESM1_ESM.docx]

Table S1 Clean reads quality metrics

| Sample | Total Raw Reads (Mb) | Total Clean Reads (Mb) | Total Clean Bases (Gb) | Clean Reads Q20 (%) | Clean Reads Q30 (%) | Clean Reads Ratio(%) |
| --- | --- | --- | --- | --- | --- | --- |
| HS30d_1 | 88.42 | 74.44 | 11.17 | *98.44* | 95.28 | 84.20 |
| HS30d_2 | 86.78 | 73.71 | 11.06 | *98.45* | 95.30 | 84.94 |
| HS30d_3 | 88.42 | 74.53 | 11.18 | *98.44* | 95.27 | 84.29 |
| HS60d_1 | 88.42 | 73.96 | 11.09 | *98.44* | 95.26 | 83.65 |
| HS60d_2 | 88.42 | 74.49 | 11.17 | *98.41* | 95.22 | 84.25 |
| HS60d_3 | 90.05 | 74.18 | 11.13 | *98.42* | 95.22 | 82.37 |
| HS90d_1 | 87.45 | 74.39 | 11.16 | *98.20* | 94.78 | 85.08 |
| HS90d_2 | 85.82 | 73.86 | 11.08 | *98.13* | 94.60 | 86.07 |
| HS90d_3 | 85.83 | 73.53 | 11.03 | *98.16* | 94.66 | 85.68 |
