# Supplementary material for: Identification of genes associated with the biosynthesis of unsaturated fatty acid and oil accumulation in herbaceous peony ‘Hangshao’ (Paeonia lactiflora ‘Hangshao’) seeds based on transcriptome analysis
Source: BMC Genomics. 2021 Feb 1;22:94. doi: 10.1186/s12864-020-07339-7 (PMC7849092; doi:10.1186/s12864-020-07339-7)
Supplement: Supplementary file 2 — Additional file 2: Table S2. Quality metrics of transcripts of seeds of Paeonia lactiflora ‘Hangshao’ [file 12864_2020_7339_MOESM2_ESM.docx]

Table S2 Quality metrics of transcripts of seeds of *Paeonia lactiflora* 'Hangshao'

| Sample | Total Number | Total Length | Mean Length | N50 | N70 | N90 | GC(%) |
| --- | --- | --- | --- | --- | --- | --- | --- |
| HS30d_1 | 60,209 | 53,854,966 | 894 | 1,527 | 913 | 341 | 41.21 |
| HS30d_2 | 73,240 | 65,889,070 | 899 | 1,543 | 910 | 343 | 40.58 |
| HS30d_3 | 62,924 | 57,723,302 | 917 | 1,536 | 935 | 357 | 40.98 |
| HS60d_1 | 54,052 | 49,432,167 | 914 | 1,519 | 934 | 360 | 41.41 |
| HS60d_2 | 54,528 | 48,804,676 | 895 | 1,518 | 910 | 344 | 41.39 |
| HS60d_3 | 59,805 | 52,925,037 | 884 | 1,508 | 897 | 337 | 41.15 |
| HS90d_1 | 75,580 | 65,638,806 | 868 | 1,502 | 857 | 327 | 40.48 |
| HS90d_2 | 73,271 | 61,153,792 | 834 | 1,448 | 815 | 313 | 40.75 |
| HS90d_3 | 70,868 | 62,037,802 | 875 | 1,505 | 872 | 333 | 40.47 |
| All-Unigene | 150,156 | 154,674,160 | 1,030 | 1,825 | 1,120 | 394 | 39.97 |
