# Supplementary figures and images for: Identification of genes associated with the biosynthesis of unsaturated fatty acid and oil accumulation in herbaceous peony ‘Hangshao’ (Paeonia lactiflora ‘Hangshao’) seeds based on transcriptome analysis
Source: BMC Genomics. 2021 Feb 1;22:94. doi: 10.1186/s12864-020-07339-7 (PMC7849092; doi:10.1186/s12864-020-07339-7)

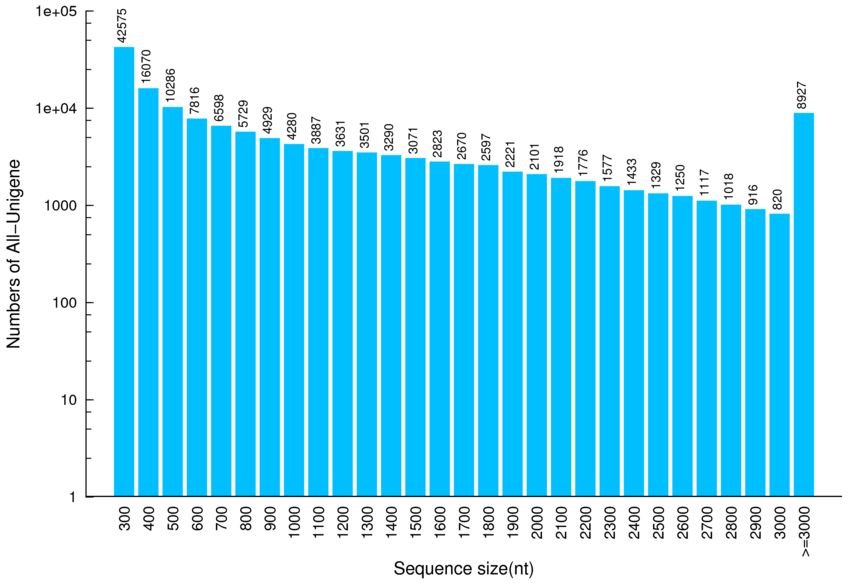


Figure S1 The length distribution of unigenes for seeds of *Paeonia lactiflora* 'Hangshao'

Supplement: Supplementary file 3 — Additional file 3: Figure S1. The length distribution of unigenes for seeds of Paeonia lactiflora ‘Hangshao’ [file 12864_2020_7339_MOESM3_ESM.docx]
