# Supplementary material for: Identification of genes associated with the biosynthesis of unsaturated fatty acid and oil accumulation in herbaceous peony ‘Hangshao’ (Paeonia lactiflora ‘Hangshao’) seeds based on transcriptome analysis
Source: BMC Genomics. 2021 Feb 1;22:94. doi: 10.1186/s12864-020-07339-7 (PMC7849092; doi:10.1186/s12864-020-07339-7)
Supplement: Supplementary file 4 — Additional file 4: Figure S2. Functional distribution of unigenes annotated for seeds of Paeonia lactiflora ‘Hangshao’ [file 12864_2020_7339_MOESM4_ESM.docx]

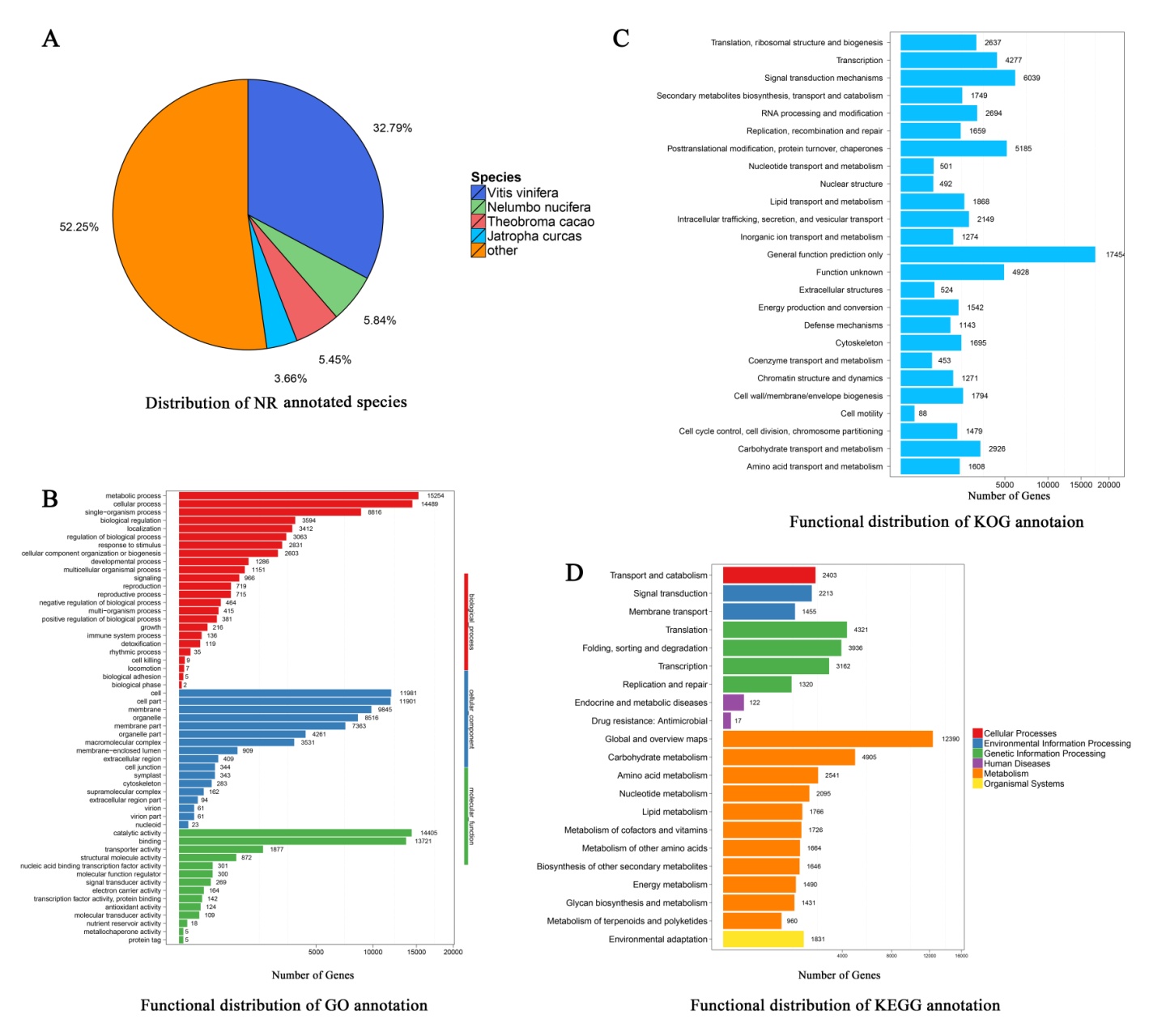


Figure S2 Functional distribution of unigenes annotated for seeds of *Paeonia lactiflora* 'Hangshao'

A: Distribution of NR annotated species; B: Functional distribution of GO annotation;

C: Functional distribution of KOG annotation; D: Functional distribution of KEGG annotation.
