# Supplementary material for: Identification of genes associated with the biosynthesis of unsaturated fatty acid and oil accumulation in herbaceous peony ‘Hangshao’ (Paeonia lactiflora ‘Hangshao’) seeds based on transcriptome analysis
Source: BMC Genomics. 2021 Feb 1;22:94. doi: 10.1186/s12864-020-07339-7 (PMC7849092; doi:10.1186/s12864-020-07339-7)
Supplement: Supplementary file 5 — Additional file 5: Table S3. Number of DEGs for GO classification [file 12864_2020_7339_MOESM5_ESM.docx]

| Table S3 Number of DEGs for GO classification | | | | |
| --- | --- | --- | --- | --- |
| Ontoloty | GO term | Number of Genes（Group I） | Number of Genes（Group II） | Number of Genes（Group III） |
| biological_process | biological adhesion | - | - | 2 |
| biological_process | biological regulation | 253 | 660 | 923 |
| biological_process | cell killing | 6 | 9 | 8 |
| biological_process | cellular component organization or biogenesis | 178 | 378 | 577 |
| biological_process | cellular process | 927 | 2433 | 3508 |
| biological_process | detoxification | 9 | 30 | 39 |
| biological_process | developmental process | 85 | 233 | 312 |
| biological_process | growth | 12 | 36 | 45 |
| biological_process | immune system process | 11 | 23 | 26 |
| biological_process | localization | 229 | 696 | 887 |
| biological_process | locomotion | 1 | - | 1 |
| biological_process | metabolic process | 1086 | 2760 | 3860 |
| biological_process | multi-organism process | 40 | 78 | 102 |
| biological_process | multicellular organismal process | 76 | 212 | 290 |
| biological_process | negative regulation of biological process | 41 | 71 | 109 |
| biological_process | positive regulation of biological process | 19 | 48 | 77 |
| biological_process | regulation of biological process | 203 | 538 | 774 |
| biological_process | reproduction | 40 | 116 | 165 |
| biological_process | reproductive process | 40 | 115 | 164 |
| biological_process | response to stimulus | 217 | 522 | 749 |
| biological_process | rhythmic process | - | 3 | 9 |
| biological_process | signaling | 64 | 211 | 275 |
| biological_process | single-organism process | 768 | 1829 | 2541 |
| cellular_component | cell | 754 | 1974 | 2848 |
| cellular_component | cell junction | 24 | 62 | 83 |
| cellular_component | cell part | 743 | 1956 | 2824 |
| cellular_component | cytoskeleton | 30 | 54 | 80 |
| cellular_component | extracellular region | 49 | 107 | 117 |
| cellular_component | extracellular region part | 4 | 18 | 20 |
| cellular_component | macromolecular complex | 212 | 403 | 716 |
| cellular_component | membrane | 709 | 2048 | 2712 |
| cellular_component | membrane part | 520 | 1610 | 2081 |
| cellular_component | membrane-enclosed lumen | 59 | 93 | 160 |
| cellular_component | nucleoid | - | 2 | 5 |
| cellular_component | organelle | 521 | 1321 | 1195 |
| cellular_component | organelle part | 269 | 618 | 976 |
| cellular_component | supramolecular complex | 21 | 37 | 55 |
| cellular_component | symplast | 24 | 62 | 83 |
| cellular_component | virion | - | 10 | 10 |
| cellular_component | virion part | - | 10 | 10 |
| molecular_function | antioxidant activity | 8 | 29 | 39 |
| molecular_function | binding | 972 | 2296 | 3267 |
| molecular_function | catalytic activity | 1176 | 2810 | 3861 |
| molecular_function | electron carrier activity | 14 | 21 | 40 |
| molecular_function | metallochaperone activity | - | - | 1 |
| molecular_function | molecular function regulator | 33 | 59 | 88 |
| molecular_function | molecular transducer activity | 14 | 26 | 33 |
| molecular_function | nucleic acid binding transcription factor activity | 19 | 58 | 90 |
| molecular_function | nutrient reservoir activity | 3 | 9 | 9 |
| molecular_function | protein tag | - | 2 | 2 |
| molecular_function | signal transducer activity | 22 | 52 | 72 |
| molecular_function | structural molecule activity | 34 | 81 | 145 |
| molecular_function | transcription factor activity, protein binding | 6 | 26 | 35 |
| molecular_function | transporter activity | 157 | 434 | 543 |
