# Supplementary material for: Identification of genes associated with the biosynthesis of unsaturated fatty acid and oil accumulation in herbaceous peony ‘Hangshao’ (Paeonia lactiflora ‘Hangshao’) seeds based on transcriptome analysis
Source: BMC Genomics. 2021 Feb 1;22:94. doi: 10.1186/s12864-020-07339-7 (PMC7849092; doi:10.1186/s12864-020-07339-7)
Supplement: Supplementary file 6 — Additional file 6: Figure S3. Functional distribution of GO-annotated DEGs for seeds of Paeonia lactiflora ‘Hangshao’ [file 12864_2020_7339_MOESM6_ESM.docx]

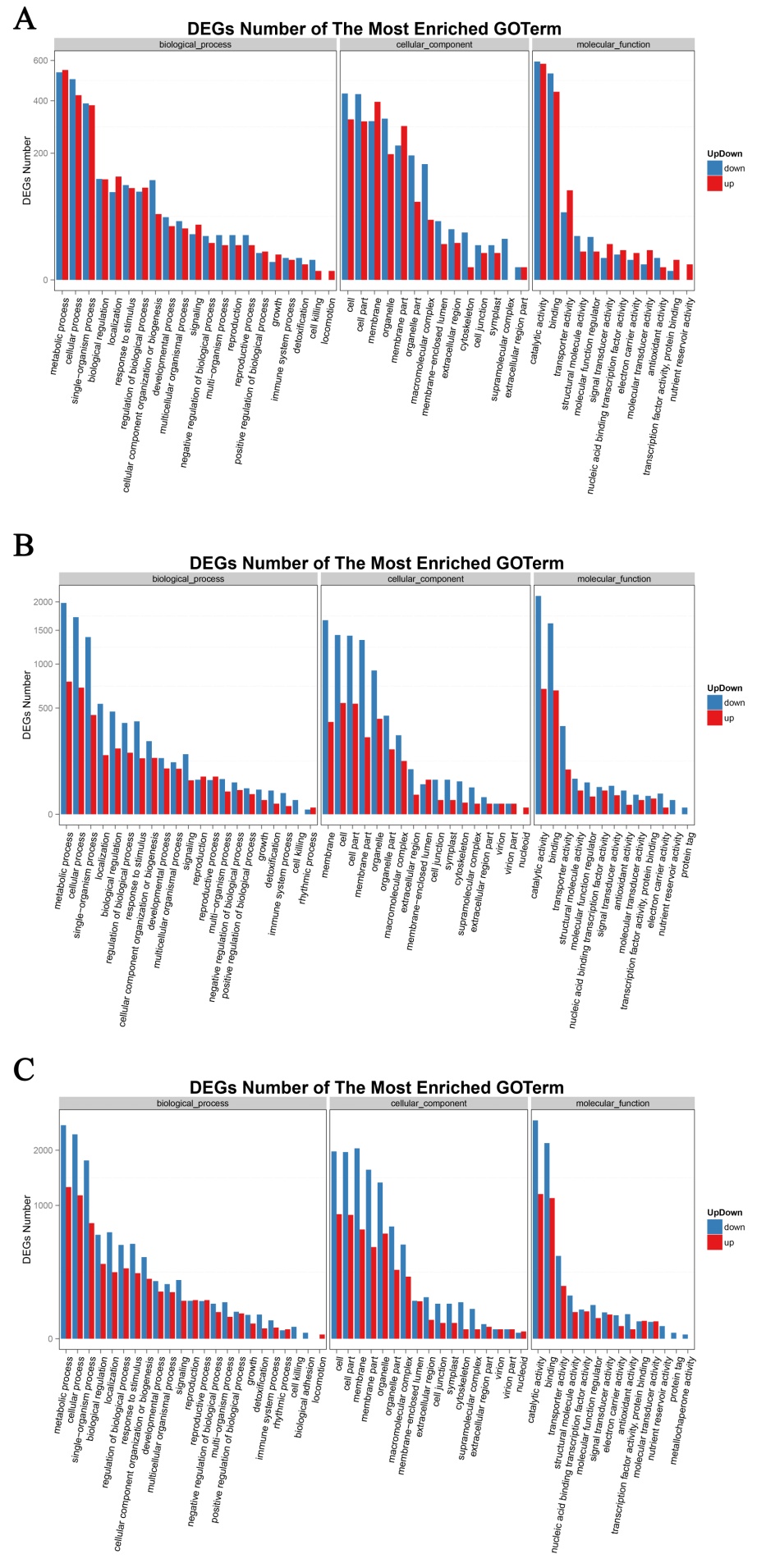


Figure S3 Functional distribution of GO- annotated DEGs for seeds of *Paeonia lactiflora* 'Hangshao'

A: 30 d vs 60 d (Group I); B: 60 d vs 90 d (Group II); C: 30 d vs 90 d (Group III)
