# Supplementary material for: Identification of genes associated with the biosynthesis of unsaturated fatty acid and oil accumulation in herbaceous peony ‘Hangshao’ (Paeonia lactiflora ‘Hangshao’) seeds based on transcriptome analysis
Source: BMC Genomics. 2021 Feb 1;22:94. doi: 10.1186/s12864-020-07339-7 (PMC7849092; doi:10.1186/s12864-020-07339-7)
Supplement: Supplementary file 7 — Additional file 7: Table S4. Number of DEGs for KEGG annotation [file 12864_2020_7339_MOESM7_ESM.docx]

| Table S4 Number of DEGs for KEGG annotation | | | | |
| --- | --- | --- | --- | --- |
| Pathway_level1 | Pathway_level2 | Number of Genes（Group I） | Number of Genes（Group II） | Number of Genes（Group III） |
| Cellular Processes | Transport and catabolism | 135 | 397 | 528 |
| Environmental Information Processing | Membrane transport | 44 | 115 | 166 |
| Environmental Information Processing | Signal transduction | 178 | 431 | 590 |
| Genetic Information Processing | Folding, sorting and degradation | 195 | 540 | 747 |
| Genetic Information Processing | Replication and repair | 80 | 142 | 231 |
| Genetic Information Processing | Transcription | 110 | 351 | 527 |
| Genetic Information Processing | Translation | 158 | 529 | 749 |
| Metabolism | Amino acid metabolism | 170 | 400 | 556 |
| Metabolism | Biosynthesis of other secondary metabolites | 157 | 332 | 421 |
| Metabolism | Carbohydrate metabolism | 352 | 879 | 1215 |
| Metabolism | Energy metabolism | 61 | 273 | 352 |
| Metabolism | Global and overview maps | 832 | 2075 | 2872 |
| Metabolism | Glycan biosynthesis and metabolism | 77 | 196 | 273 |
| Metabolism | Lipid metabolism | 191 | 424 | 544 |
| Metabolism | Metabolism of cofactors and vitamins | 75 | 247 | 356 |
| Metabolism | Metabolism of other amino acids | 106 | 242 | 327 |
| Metabolism | Metabolism of terpenoids and polyketides | 90 | 175 | 248 |
| Metabolism | Nucleotide metabolism | 100 | 243 | 391 |
| Organismal Systems | Environmental adaptation | 112 | 310 | 423 |
