# Supplementary material for: Identification of genes associated with the biosynthesis of unsaturated fatty acid and oil accumulation in herbaceous peony ‘Hangshao’ (Paeonia lactiflora ‘Hangshao’) seeds based on transcriptome analysis
Source: BMC Genomics. 2021 Feb 1;22:94. doi: 10.1186/s12864-020-07339-7 (PMC7849092; doi:10.1186/s12864-020-07339-7)
Supplement: Supplementary file 9 — Additional file 9: Table S6. Number of DEGs for KEGG pathway annotation in Groups I, II and III [file 12864_2020_7339_MOESM9_ESM.docx]

| **Table S6-3 Number of DEGs for KEGG pathway annotation in Group III** | | | | | | | | |  |  |  |
| --- | --- | --- | --- | --- | --- | --- | --- | --- | --- | --- | --- |
| **#** | **Pathway** | **DEGs genes with pathway annotation  (11268)** | **All genes with pathway annotation  (49630)** | **Pvalue** | **Qvalue** | **Pathway ID** | **Level 1** | **Level 2** |  |  |  |
| 1 | [Glycosaminoglycan degradation](file:///D:\Programs\ProductionJournal\Temp\renamed_3c089.xlsx#RANGE!gene1) | 60 (0.53%) | 142 (0.29%) | 1.66959E-07 | 1.39463E-05 | ko00531 | Metabolism | Glycan biosynthesis and metabolism | | |  |
| 2 | [Circadian rhythm - plant](file:///D:\Programs\ProductionJournal\Temp\renamed_3c089.xlsx#RANGE!gene2) | 112 (0.99%) | 317 (0.64%) | 2.10398E-07 | 1.39463E-05 | ko04712 | Organismal Systems | Environmental adaptation | |  |  |
| 3 | [Plant hormone signal transduction](file:///D:\Programs\ProductionJournal\Temp\renamed_3c089.xlsx#RANGE!gene3) | 334 (2.96%) | 1152 (2.32%) | 3.1223E-07 | 1.39463E-05 | ko04075 | Environmental Information Processing | Signal transduction | |  |  |
| 4 | [Glycerolipid metabolism](file:///D:\Programs\ProductionJournal\Temp\renamed_3c089.xlsx#RANGE!gene4) | 124 (1.1%) | 366 (0.74%) | 6.53516E-07 | 1.82364E-05 | ko00561 | Metabolism | Lipid metabolism |  |  |  |
| 5 | [Arachidonic acid metabolism](file:///D:\Programs\ProductionJournal\Temp\renamed_3c089.xlsx#RANGE!gene5) | 45 (0.4%) | 100 (0.2%) | 6.80462E-07 | 1.82364E-05 | ko00590 | Metabolism | Lipid metabolism |  |  |  |
| 6 | [Indole alkaloid biosynthesis](file:///D:\Programs\ProductionJournal\Temp\renamed_3c089.xlsx#RANGE!gene6) | 40 (0.35%) | 87 (0.18%) | 1.41458E-06 | 3.15922E-05 | ko00901 | Metabolism | Biosynthesis of other secondary metabolites | | | |
| 7 | [Benzoxazinoid biosynthesis](file:///D:\Programs\ProductionJournal\Temp\renamed_3c089.xlsx#RANGE!gene7) | 21 (0.19%) | 36 (0.07%) | 4.33144E-06 | 8.29162E-05 | ko00402 | Metabolism | Biosynthesis of other secondary metabolites | | | |
| 8 | [Photosynthesis - antenna proteins](file:///D:\Programs\ProductionJournal\Temp\renamed_3c089.xlsx#RANGE!gene8) | 21 (0.19%) | 39 (0.08%) | 2.35455E-05 | 0.000394387 | ko00196 | Metabolism | Energy metabolism | |  |  |
| 9 | [Fructose and mannose metabolism](file:///D:\Programs\ProductionJournal\Temp\renamed_3c089.xlsx#RANGE!gene9) | 88 (0.78%) | 264 (0.53%) | 4.93131E-05 | 0.000734217 | ko00051 | Metabolism | Carbohydrate metabolism | |  |  |
| 10 | [Glycosphingolipid biosynthesis - ganglio series](file:///D:\Programs\ProductionJournal\Temp\renamed_3c089.xlsx#RANGE!gene10) | 41 (0.36%) | 104 (0.21%) | 9.56806E-05 | 0.00128212 | ko00604 | Metabolism | Glycan biosynthesis and metabolism | | |  |
| 11 | [Steroid biosynthesis](file:///D:\Programs\ProductionJournal\Temp\renamed_3c089.xlsx#RANGE!gene11) | 52 (0.46%) | 142 (0.29%) | 0.000120962 | 0.001473536 | ko00100 | Metabolism | Lipid metabolism |  |  |  |
| 12 | [Sphingolipid metabolism](file:///D:\Programs\ProductionJournal\Temp\renamed_3c089.xlsx#RANGE!gene12) | 71 (0.63%) | 212 (0.43%) | 0.000211346 | 0.00236003 | ko00600 | Metabolism | Lipid metabolism |  |  |  |
| 13 | [Vitamin B6 metabolism](file:///D:\Programs\ProductionJournal\Temp\renamed_3c089.xlsx#RANGE!gene13) | 31 (0.28%) | 75 (0.15%) | 0.000242033 | 0.002494797 | ko00750 | Metabolism | Metabolism of cofactors and vitamins | | |  |
| 14 | [Flavonoid biosynthesis](file:///D:\Programs\ProductionJournal\Temp\renamed_3c089.xlsx#RANGE!gene14) | 56 (0.5%) | 161 (0.32%) | 0.000321835 | 0.003080422 | ko00941 | Metabolism | Biosynthesis of other secondary metabolites | | | |
| 15 | [Cysteine and methionine metabolism](file:///D:\Programs\ProductionJournal\Temp\renamed_3c089.xlsx#RANGE!gene15) | 134 (1.19%) | 455 (0.92%) | 0.000478096 | 0.004270995 | ko00270 | Metabolism | Amino acid metabolism | |  |  |
| 16 | [Cutin, suberine and wax biosynthesis](file:///D:\Programs\ProductionJournal\Temp\renamed_3c089.xlsx#RANGE!gene16) | 34 (0.3%) | 88 (0.18%) | 0.000560315 | 0.004502275 | ko00073 | Metabolism | Lipid metabolism |  |  |  |
| 17 | [Limonene and pinene degradation](file:///D:\Programs\ProductionJournal\Temp\renamed_3c089.xlsx#RANGE!gene17) | 26 (0.23%) | 62 (0.12%) | 0.000571184 | 0.004502275 | ko00903 | Metabolism | Metabolism of terpenoids and polyketides | | | |
| 18 | [Biosynthesis of secondary metabolites](file:///D:\Programs\ProductionJournal\Temp\renamed_3c089.xlsx#RANGE!gene18) | 1361 (12.08%) | 5577 (11.24%) | 0.000741373 | 0.005519108 | ko01110 | Metabolism | Global and overview maps | |  |  |
| 19 | [Riboflavin metabolism](file:///D:\Programs\ProductionJournal\Temp\renamed_3c089.xlsx#RANGE!gene19) | 28 (0.25%) | 71 (0.14%) | 0.001144844 | 0.008074163 | ko00740 | Metabolism | Metabolism of cofactors and vitamins | | |  |
| 20 | [Pentose phosphate pathway](file:///D:\Programs\ProductionJournal\Temp\renamed_3c089.xlsx#RANGE!gene20) | 83 (0.74%) | 269 (0.54%) | 0.001224745 | 0.008205792 | ko00030 | Metabolism | Carbohydrate metabolism | |  |  |
| 21 | [Stilbenoid, diarylheptanoid and gingerol biosynthesis](file:///D:\Programs\ProductionJournal\Temp\renamed_3c089.xlsx#RANGE!gene21) | 42 (0.37%) | 123 (0.25%) | 0.002493052 | 0.01590805 | ko00945 | Metabolism | Biosynthesis of other secondary metabolites | | | |
| 22 | [Carbon metabolism](file:///D:\Programs\ProductionJournal\Temp\renamed_3c089.xlsx#RANGE!gene22) | 329 (2.92%) | 1271 (2.56%) | 0.003746713 | 0.02265873 | ko01200 | Metabolism | Global and overview maps | |  |  |
| 23 | [MAPK signaling pathway - plant](file:///D:\Programs\ProductionJournal\Temp\renamed_3c089.xlsx#RANGE!gene23) | 271 (2.41%) | 1033 (2.08%) | 0.003889185 | 0.02265873 | ko04016 | Environmental Information Processing | Signal transduction | |  |  |
| 24 | [Tryptophan metabolism](file:///D:\Programs\ProductionJournal\Temp\renamed_3c089.xlsx#RANGE!gene24) | 55 (0.49%) | 174 (0.35%) | 0.0042534 | 0.02374815 | ko00380 | Metabolism | Amino acid metabolism | |  |  |
| 25 | [Glycolysis / Gluconeogenesis](file:///D:\Programs\ProductionJournal\Temp\renamed_3c089.xlsx#RANGE!gene25) | 153 (1.36%) | 560 (1.13%) | 0.005757766 | 0.03086163 | ko00010 | Metabolism | Carbohydrate metabolism | |  |  |
| 26 | [Thiamine metabolism](file:///D:\Programs\ProductionJournal\Temp\renamed_3c089.xlsx#RANGE!gene26) | 34 (0.3%) | 100 (0.2%) | 0.006529379 | 0.03365141 | ko00730 | Metabolism | Metabolism of cofactors and vitamins | | |  |
| 27 | [Starch and sucrose metabolism](file:///D:\Programs\ProductionJournal\Temp\renamed_3c089.xlsx#RANGE!gene27) | 302 (2.68%) | 1176 (2.37%) | 0.008141024 | 0.0404036 | ko00500 | Metabolism | Carbohydrate metabolism | |  |  |
| 28 | [Phenylalanine, tyrosine and tryptophan biosynthesis](file:///D:\Programs\ProductionJournal\Temp\renamed_3c089.xlsx#RANGE!gene28) | 72 (0.64%) | 245 (0.49%) | 0.008925483 | 0.04271481 | ko00400 | Metabolism | Amino acid metabolism | |  |  |
| 29 | [Folate biosynthesis](file:///D:\Programs\ProductionJournal\Temp\renamed_3c089.xlsx#RANGE!gene29) | 30 (0.27%) | 88 (0.18%) | 0.009764816 | 0.04512018 | ko00790 | Metabolism | Metabolism of cofactors and vitamins | | |  |
| 30 | [Sesquiterpenoid and triterpenoid biosynthesis](file:///D:\Programs\ProductionJournal\Temp\renamed_3c089.xlsx#RANGE!gene30) | 36 (0.32%) | 110 (0.22%) | 0.01021152 | 0.04561146 | ko00909 | Metabolism | Metabolism of terpenoids and polyketides | | | |
| 31 | [Flavone and flavonol biosynthesis](file:///D:\Programs\ProductionJournal\Temp\renamed_3c089.xlsx#RANGE!gene31) | 15 (0.13%) | 37 (0.07%) | 0.01148584 | 0.04899739 | ko00944 | Metabolism | Biosynthesis of other secondary metabolites | | | |
| 32 | [Mannose type O-glycan biosynthesis](file:///D:\Programs\ProductionJournal\Temp\renamed_3c089.xlsx#RANGE!gene32) | 3 (0.03%) | 3 (0.01%) | 0.01170087 | 0.04899739 | ko00515 | Metabolism | Glycan biosynthesis and metabolism | | |  |
| 33 | [Nitrogen metabolism](file:///D:\Programs\ProductionJournal\Temp\renamed_3c089.xlsx#RANGE!gene33) | 56 (0.5%) | 188 (0.38%) | 0.01457331 | 0.0581119 | ko00910 | Metabolism | Energy metabolism | |  |  |
| 34 | [Glycosphingolipid biosynthesis - globo and isoglobo series](file:///D:\Programs\ProductionJournal\Temp\renamed_3c089.xlsx#RANGE!gene34) | 11 (0.1%) | 25 (0.05%) | 0.01474481 | 0.0581119 | ko00603 | Metabolism | Glycan biosynthesis and metabolism | | |  |
| 35 | [Diterpenoid biosynthesis](file:///D:\Programs\ProductionJournal\Temp\renamed_3c089.xlsx#RANGE!gene35) | 37 (0.33%) | 118 (0.24%) | 0.01889674 | 0.07234752 | ko00904 | Metabolism | Metabolism of terpenoids and polyketides | | | |
| 36 | [Arginine and proline metabolism](file:///D:\Programs\ProductionJournal\Temp\renamed_3c089.xlsx#RANGE!gene36) | 58 (0.51%) | 199 (0.4%) | 0.02047109 | 0.07494997 | ko00330 | Metabolism | Amino acid metabolism | |  |  |
| 37 | [Porphyrin and chlorophyll metabolism](file:///D:\Programs\ProductionJournal\Temp\renamed_3c089.xlsx#RANGE!gene37) | 59 (0.52%) | 203 (0.41%) | 0.02069514 | 0.07494997 | ko00860 | Metabolism | Metabolism of cofactors and vitamins | | |  |
| 38 | [Fatty acid biosynthesis](file:///D:\Programs\ProductionJournal\Temp\renamed_3c089.xlsx#RANGE!gene38) | 51 (0.45%) | 176 (0.35%) | 0.03122039 | 0.110093 | ko00061 | Metabolism | Lipid metabolism |  |  |  |
| 39 | [Carbon fixation in photosynthetic organisms](file:///D:\Programs\ProductionJournal\Temp\renamed_3c089.xlsx#RANGE!gene39) | 115 (1.02%) | 435 (0.88%) | 0.03690553 | 0.1268036 | ko00710 | Metabolism | Energy metabolism | |  |  |
| 40 | [Linoleic acid metabolism](file:///D:\Programs\ProductionJournal\Temp\renamed_3c089.xlsx#RANGE!gene40) | 40 (0.35%) | 136 (0.27%) | 0.04149304 | 0.1390017 | ko00591 | Metabolism | Lipid metabolism |  |  |  |
| 41 | [Biotin metabolism](file:///D:\Programs\ProductionJournal\Temp\renamed_3c089.xlsx#RANGE!gene41) | 25 (0.22%) | 79 (0.16%) | 0.04253323 | 0.139011 | ko00780 | Metabolism | Metabolism of cofactors and vitamins | | |  |
