# Supplementary material for: Identification of genes associated with the biosynthesis of unsaturated fatty acid and oil accumulation in herbaceous peony ‘Hangshao’ (Paeonia lactiflora ‘Hangshao’) seeds based on transcriptome analysis
Source: BMC Genomics. 2021 Feb 1;22:94. doi: 10.1186/s12864-020-07339-7 (PMC7849092; doi:10.1186/s12864-020-07339-7)
Supplement: Supplementary file 10 — Additional file 10: Table S7. Number of DEGs in 14 KEGG lipid metabolism pathways [file 12864_2020_7339_MOESM10_ESM.docx]

| Table S7 Number of DEGs in 14 KEGG lipid metabolism pathways | | | | | | | |
| --- | --- | --- | --- | --- | --- | --- | --- |
| Pathway | Ko | Gene numbers | | | | | |
|  |  | 30d vs 60 d (Group I) | | 60d vs 90d (Group II) | | 30d vs 90d (Group III) | |
|  |  | Cluter frequency | P-value | Cluter frequency | P-value | Cluter frequency | P-value |
| Fatty acid biosythesis | ko00061 | 22/176 | 0.00247029 | 41/176 | 0.00774033 | 51/176 | 0.03122039 |
| Fatty acid elongation | ko00062 | 6/140 | 0.89811220 | 15/140 | 0.97204830 | 29/140 | 0.74321080 |
| Fatty acid degradation | ko00071 | 16/173 | 0.98153580 | 29/173 | 0.42953420 | 39/173 | 0.54980450 |
| Synthsis and degradation of ketone bodies | ko00072 | 2/17 | 0.30373830 | 6/17 | 0.04275777 | 5/17 | 0.33887620 |
| Cutin, suberin and wax biosynthesis | ko00073 | 26/88 | 0.00000000 | 19/88 | 0.10420110 | 34/88 | 0.00056031 |
| Steoid biosynthesis | ko00100 | 11/142 | 0.31752210 | 42/142 | 0.00003764 | 52/142 | 0.00012096 |
| Glycerolipid metabolism | ko00561 | 48/366 | 0.00000313 | 100/366 | 0.00000003 | 124/366 | 0.00000065 |
| Ether lipid metabolism | ko00565 | 12/157 | 0.32267160 | 23/157 | 0.71523280 | 37/157 | 0.42864620 |
| Glycerophospholipid metabolism | ko00564 | 23/376 | 0.64758950 | 68/376 | 0.15560550 | 90/376 | 0.30194270 |
| Sphingolipid metabolism | ko00600 | 24/212 | 0.00584644 | 51/212 | 0.00161255 | 71/212 | 0.00021135 |
| Arachidonic acid metabolism | ko00590 | 14/100 | 0.00523707 | 38/100 | 0.00000010 | 45/100 | 0.00000068 |
| Linoleic acid metabolism | ko00591 | 11/136 | 0.26972770 | 37/136 | 0.00064396 | 40/136 | 0.04149304 |
| Alpha-linolenic acid metabolism | ko00592 | 20/244 | 0.16963770 | 49/244 | 0.05400820 | 55/244 | 0.54929740 |
| Biosynthsis of unsaturated fatty acid | ko01040 | 5/147 | 0.96557140 | 31/147 | 0.06346957 | 36/147 | 0.33231490 |
|  |  |  |  |  |  |  |  |
| *The colored block are shown as enrichment | |  |  |  |  |  |  |
