# Supplementary material for: Identification of genes associated with the biosynthesis of unsaturated fatty acid and oil accumulation in herbaceous peony ‘Hangshao’ (Paeonia lactiflora ‘Hangshao’) seeds based on transcriptome analysis
Source: BMC Genomics. 2021 Feb 1;22:94. doi: 10.1186/s12864-020-07339-7 (PMC7849092; doi:10.1186/s12864-020-07339-7)
Supplement: Supplementary file 11 — Additional file 11: Table S8. All DEGs annotated to lipid metabolism [file 12864_2020_7339_MOESM11_ESM.docx]

| Table S8: All DEGs annotated to lipid metabolism | | | | | | | | | | |  |
| --- | --- | --- | --- | --- | --- | --- | --- | --- | --- | --- | --- |
| Gene ID | Definition | HS30d_1_FPKM | HS30d_2_FPKM | HS30d_3_FPKM | HS60d_1_FPKM | HS60d_2_FPKM | HS60d_3_FPKM | HS90d_1_FPKM | HS90d_2_FPKM | HS90d_3_FPKM | |
| CL15985.Contig2_All | K07513 acetyl-CoA acyltransferase 1 [EC:2.3.1.16] | 0 | 0 | 0 | 0.03 | 0.02 | 0.05 | 0.07 | 0 | 1.63 |  |
| CL1727.Contig4_All | K07513 acetyl-CoA acyltransferase 1 [EC:2.3.1.16] | 15.37 | 23.94 | 16.59 | 12.9 | 9.79 | 9.12 | 9.23 | 7.18 | 2.02 |  |
| Unigene1380_All | K07513 acetyl-CoA acyltransferase 1 [EC:2.3.1.16] | 0 | 0.14 | 0 | 0 | 0.13 | 0 | 0.96 | 0.33 | 0.74 |  |
| Unigene41549_All | K15403 fatty acid omega-hydroxy dehydrogenase [EC:1.1.-.-] | 1.4 | 3.03 | 1.27 | 0.05 | 0.14 | 0.09 | 0 | 0 | 0 |  |
| CL2966.Contig1_All | K04711 dihydroceramidase [EC:3.5.1.-] | 0.86 | 0.74 | 1.74 | 1.98 | 1.38 | 1.51 | 8.5 | 7.15 | 8.11 |  |
| Unigene23060_All | K00232 acyl-CoA oxidase [EC:1.3.3.6] | 34.54 | 36.9 | 35.96 | 69.27 | 56.92 | 61.87 | 177.55 | 106.5 | 138.29 |  |
| Unigene38665_All | K00232 acyl-CoA oxidase [EC:1.3.3.6] | 1.23 | 1.55 | 1.11 | 2.23 | 0.82 | 1.78 | 0.14 | 0.4 | 0.43 |  |
| Unigene44378_All | K00232 acyl-CoA oxidase [EC:1.3.3.6] | 0 | 0.14 | 0.14 | 0.04 | 0.13 | 0 | 0.54 | 1.25 | 1.11 |  |
| CL3411.Contig1_All | K00001 alcohol dehydrogenase [EC:1.1.1.1] | 4.36 | 6.19 | 4.51 | 3.22 | 3.07 | 2.99 | 1.47 | 0.72 | 0.83 |  |
| CL4342.Contig1_All | K00001 alcohol dehydrogenase [EC:1.1.1.1] | 0.34 | 0.62 | 0.77 | 0.41 | 2.04 | 2.31 | 0 | 0 | 0 |  |
| Unigene23270_All | K00001 alcohol dehydrogenase [EC:1.1.1.1] | 13.6 | 17.21 | 12.23 | 10.78 | 9.61 | 8.71 | 4.99 | 2.73 | 2.85 |  |
| CL10597.Contig3_All | K18857 alcohol dehydrogenase class-P [EC:1.1.1.1] | 0.19 | 0.66 | 0.19 | 0.8 | 0.11 | 0.5 | 1.25 | 0.98 | 1.94 |  |
| CL1931.Contig1_All | K18857 alcohol dehydrogenase class-P [EC:1.1.1.1] | 62.83 | 84.46 | 66.85 | 82.14 | 49.2 | 67.42 | 5.9 | 4.89 | 6.23 |  |
| CL1931.Contig2_All | K18857 alcohol dehydrogenase class-P [EC:1.1.1.1] | 17.48 | 23.3 | 29.22 | 36.46 | 20.81 | 32.87 | 3.99 | 5.78 | 3.77 |  |
| CL1931.Contig3_All | K18857 alcohol dehydrogenase class-P [EC:1.1.1.1] | 73.42 | 89.88 | 72.79 | 94.39 | 51.19 | 73.44 | 7.51 | 6.52 | 8.73 |  |
| CL1931.Contig4_All | K18857 alcohol dehydrogenase class-P [EC:1.1.1.1] | 19.43 | 24.49 | 32.62 | 39.98 | 22.17 | 35.5 | 4.74 | 7.24 | 4.56 |  |
| CL1931.Contig5_All | K18857 alcohol dehydrogenase class-P [EC:1.1.1.1] | 22.25 | 29.19 | 23.5 | 25.53 | 25.27 | 34.15 | 0.25 | 1.32 | 3.41 |  |
| CL4171.Contig1_All | K18857 alcohol dehydrogenase class-P [EC:1.1.1.1] | 40.17 | 42.36 | 41.29 | 53.19 | 43.63 | 36.53 | 16.46 | 11.13 | 14.1 |  |
| CL585.Contig4_All | K18857 alcohol dehydrogenase class-P [EC:1.1.1.1] | 89.42 | 84.15 | 91.33 | 3.72 | 2.6 | 2.63 | 0 | 0.7 | 0 |  |
| CL585.Contig5_All | K18857 alcohol dehydrogenase class-P [EC:1.1.1.1] | 909.07 | 944.69 | 857.31 | 237.77 | 166 | 199.15 | 113.5 | 152.6 | 136.2 |  |
| CL585.Contig6_All | K18857 alcohol dehydrogenase class-P [EC:1.1.1.1] | 585.88 | 559.15 | 529.98 | 64.14 | 47 | 54.9 | 0 | 0 | 0 |  |
| CL585.Contig7_All | K18857 alcohol dehydrogenase class-P [EC:1.1.1.1] | 249.1 | 251.52 | 234.08 | 33.01 | 28.12 | 30.85 | 4.51 | 0 | 1.72 |  |
| CL585.Contig9_All | K18857 alcohol dehydrogenase class-P [EC:1.1.1.1] | 0.97 | 1.35 | 0.73 | 0.46 | 0.08 | 0.16 | 1.78 | 2.17 | 2.4 |  |
| CL656.Contig1_All | K18857 alcohol dehydrogenase class-P [EC:1.1.1.1] | 0 | 0.07 | 0.21 | 0.5 | 1.44 | 0.66 | 2.88 | 1.33 | 1.3 |  |
| CL656.Contig5_All | K18857 alcohol dehydrogenase class-P [EC:1.1.1.1] | 131.33 | 156.55 | 159.13 | 253.06 | 459.61 | 248.59 | 1421.5 | 695.47 | 993.27 |  |
| CL656.Contig7_All | K18857 alcohol dehydrogenase class-P [EC:1.1.1.1] | 0.64 | 0.88 | 0.96 | 2.3 | 2.83 | 2.37 | 3.8 | 2.24 | 5.65 |  |
| CL656.Contig8_All | K18857 alcohol dehydrogenase class-P [EC:1.1.1.1] | 0.46 | 0.37 | 0.82 | 0.88 | 0.81 | 0.42 | 3.67 | 2.08 | 3.83 |  |
| CL656.Contig9_All | K18857 alcohol dehydrogenase class-P [EC:1.1.1.1] | 0.31 | 0.28 | 0.23 | 0.86 | 1.55 | 0.97 | 3.26 | 1.68 | 2.01 |  |
| CL4829.Contig2_All | K00121 S-(hydroxymethyl)glutathione dehydrogenase / alcohol dehydrogenase [EC:1.1.1.284 1.1.1.1] | 5.04 | 4.52 | 7.9 | 2 | 2.07 | 2.56 | 2.76 | 2.35 | 1.71 |  |
| CL4829.Contig4_All | K00121 S-(hydroxymethyl)glutathione dehydrogenase / alcohol dehydrogenase [EC:1.1.1.284 1.1.1.1] | 6.55 | 4.26 | 4.28 | 1.23 | 0.93 | 1.82 | 2.81 | 2.73 | 26.01 |  |
| CL4829.Contig5_All | K00121 S-(hydroxymethyl)glutathione dehydrogenase / alcohol dehydrogenase [EC:1.1.1.284 1.1.1.1] | 65.15 | 79.55 | 66.47 | 23.57 | 18.37 | 19 | 49.7 | 51.94 | 30.72 |  |
| CL9131.Contig1_All | K00121 S-(hydroxymethyl)glutathione dehydrogenase / alcohol dehydrogenase [EC:1.1.1.284 1.1.1.1] | 0 | 0.05 | 0.04 | 0.04 | 0.33 | 0.08 | 1.42 | 1.07 | 2.77 |  |
| Unigene27072_All | K00121 S-(hydroxymethyl)glutathione dehydrogenase / alcohol dehydrogenase [EC:1.1.1.284 1.1.1.1] | 4.13 | 6.5 | 3.52 | 0.95 | 0.68 | 1.08 | 0 | 0 | 0 |  |
| Unigene41234_All | K00121 S-(hydroxymethyl)glutathione dehydrogenase / alcohol dehydrogenase [EC:1.1.1.284 1.1.1.1] | 0 | 0 | 0 | 0 | 0.15 | 0.15 | 2.8 | 2.78 | 3.44 |  |
| CL15410.Contig1_All | K01517 manganese-dependent ADP-ribose/CDP-alcohol diphosphatase [EC:3.6.1.13 3.6.1.16 3.6.1.53] | 0.4 | 0.49 | 0.4 | 2.7 | 3.52 | 1.74 | 13.47 | 44.36 | 31.59 |  |
| CL15410.Contig2_All | K01517 manganese-dependent ADP-ribose/CDP-alcohol diphosphatase [EC:3.6.1.13 3.6.1.16 3.6.1.53] | 0.36 | 0.11 | 0.1 | 1.23 | 1.23 | 0.45 | 12.1 | 34.82 | 29.65 |  |
| CL15410.Contig3_All | K01517 manganese-dependent ADP-ribose/CDP-alcohol diphosphatase [EC:3.6.1.13 3.6.1.16 3.6.1.53] | 0.27 | 0.62 | 0.44 | 1.68 | 2.86 | 1.74 | 2.91 | 4.78 | 4.33 |  |
| CL6518.Contig2_All | K01517 manganese-dependent ADP-ribose/CDP-alcohol diphosphatase [EC:3.6.1.13 3.6.1.16 3.6.1.53] | 1.26 | 1.58 | 1.84 | 1.51 | 0.95 | 0.88 | 0.3 | 0.33 | 0.98 |  |
| CL5515.Contig2_All | K00011 aldehyde reductase [EC:1.1.1.21] | 1.74 | 1.75 | 1.19 | 6.85 | 2.5 | 2.03 | 0.92 | 32.04 | 47.46 |  |
| CL5515.Contig5_All | K00011 aldehyde reductase [EC:1.1.1.21] | 12.32 | 11.12 | 9.73 | 10.71 | 3.48 | 7.27 | 4.11 | 3.42 | 0.69 |  |
| CL5515.Contig7_All | K00011 aldehyde reductase [EC:1.1.1.21] | 0.1 | 0.05 | 0 | 6.08 | 6.7 | 0.55 | 44.61 | 101.47 | 38.25 |  |
| CL5515.Contig8_All | K00011 aldehyde reductase [EC:1.1.1.21] | 14.34 | 7.59 | 3.31 | 13.44 | 2 | 9 | 3.19 | 1.93 | 1.29 |  |
| CL8485.Contig2_All | K00011 aldehyde reductase [EC:1.1.1.21] | 3.79 | 5.02 | 4.28 | 4.39 | 3.5 | 1.81 | 19.29 | 28.82 | 24.63 |  |
| Unigene16811_All | K00011 aldehyde reductase [EC:1.1.1.21] | 0.28 | 0.29 | 0.34 | 2.01 | 0.7 | 0.87 | 0 | 0 | 0 |  |
| Unigene20244_All | K00011 aldehyde reductase [EC:1.1.1.21] | 11.55 | 10.77 | 13.92 | 5.06 | 4.22 | 3.21 | 3.7 | 3.99 | 3.7 |  |
| CL476.Contig2_All | K00128 aldehyde dehydrogenase (NAD+) [EC:1.2.1.3] | 256.18 | 293.28 | 289.8 | 141.02 | 122.18 | 124.69 | 52.1 | 30.57 | 39.92 |  |
| CL476.Contig3_All | K00128 aldehyde dehydrogenase (NAD+) [EC:1.2.1.3] | 249.22 | 278.74 | 279.44 | 141.57 | 120.15 | 119 | 47.38 | 30.41 | 35.29 |  |
| Unigene17379_All | K00128 aldehyde dehydrogenase (NAD+) [EC:1.2.1.3] | 11.91 | 12.26 | 13.02 | 0.03 | 0.03 | 0.03 | 0 | 0 | 0 |  |
| Unigene33847_All | K00128 aldehyde dehydrogenase (NAD+) [EC:1.2.1.3] | 7.86 | 8.1 | 6.17 | 15.54 | 11.28 | 9.86 | 13.06 | 19.91 | 20.46 |  |
| CL7300.Contig2_All | K14085 aldehyde dehydrogenase family 7 member A1 [EC:1.2.1.31 1.2.1.8 1.2.1.3] | 0.42 | 1 | 0.78 | 0.46 | 0.17 | 0.29 | 3 | 5.35 | 7.33 |  |
| CL7300.Contig3_All | K14085 aldehyde dehydrogenase family 7 member A1 [EC:1.2.1.31 1.2.1.8 1.2.1.3] | 0.41 | 0.8 | 0.58 | 0.29 | 0.08 | 0.25 | 3.46 | 1.43 | 0.8 |  |
| CL7300.Contig4_All | K14085 aldehyde dehydrogenase family 7 member A1 [EC:1.2.1.31 1.2.1.8 1.2.1.3] | 0.25 | 0.25 | 0.3 | 0.39 | 0.35 | 0.26 | 0.42 | 4.16 | 5.36 |  |
| CL12584.Contig1_All | K10525 allene oxide cyclase [EC:5.3.99.6] | 33.22 | 30.78 | 25.47 | 19.38 | 17.49 | 15.33 | 7.73 | 7.71 | 9.27 |  |
| CL6163.Contig1_All | K10525 allene oxide cyclase [EC:5.3.99.6] | 2.16 | 3.37 | 14.26 | 2.38 | 1.48 | 3.36 | 0 | 0 | 0 |  |
| CL6163.Contig2_All | K10525 allene oxide cyclase [EC:5.3.99.6] | 0.21 | 2 | 0.27 | 0.59 | 0 | 0 | 0 | 0 | 0 |  |
| CL6163.Contig3_All | K10525 allene oxide cyclase [EC:5.3.99.6] | 1.1 | 0.39 | 1.11 | 0.47 | 0 | 0.24 | 0 | 0.13 | 0 |  |
| Unigene30026_All | K10525 allene oxide cyclase [EC:5.3.99.6] | 4.47 | 2.81 | 4.26 | 13.5 | 13.74 | 14.05 | 0.73 | 1.49 | 1.01 |  |
| Unigene41967_All | K10525 allene oxide cyclase [EC:5.3.99.6] | 0.33 | 2.35 | 1.67 | 0.16 | 0 | 0 | 0 | 0 | 0 |  |
| CL1370.Contig6_All | K01723 hydroperoxide dehydratase [EC:4.2.1.92] | 0 | 0.16 | 0 | 0 | 0 | 0 | 0.13 | 0.06 | 0.44 |  |
| Unigene4688_All | K01723 hydroperoxide dehydratase [EC:4.2.1.92] | 0 | 0.16 | 0.05 | 0.27 | 0.66 | 0.3 | 2.23 | 1.01 | 1.6 |  |
| CL3334.Contig2_All | K12349 neutral ceramidase [EC:3.5.1.23] | 0.17 | 0.1 | 0 | 0.56 | 1.25 | 1.15 | 0 | 4.25 | 0 |  |
| CL3334.Contig9_All | K12349 neutral ceramidase [EC:3.5.1.23] | 33.36 | 44.28 | 36.47 | 15.72 | 12.25 | 11.75 | 7.42 | 5.68 | 6.28 |  |
| CL6932.Contig1_All | K01961 acetyl-CoA carboxylase, biotin carboxylase subunit [EC:6.4.1.2 6.3.4.14] | 0.82 | 1.42 | 1.28 | 0.91 | 0.69 | 1.07 | 0 | 0 | 0 |  |
| CL6932.Contig2_All | K01961 acetyl-CoA carboxylase, biotin carboxylase subunit [EC:6.4.1.2 6.3.4.14] | 5.94 | 6.99 | 6.24 | 1.51 | 1.01 | 0.99 | 0.3 | 0.29 | 0.71 |  |
| CL6932.Contig3_All | K01961 acetyl-CoA carboxylase, biotin carboxylase subunit [EC:6.4.1.2 6.3.4.14] | 34.36 | 33.8 | 35.47 | 7.64 | 6.71 | 7.86 | 2.12 | 1.29 | 1.19 |  |
| CL6932.Contig4_All | K01961 acetyl-CoA carboxylase, biotin carboxylase subunit [EC:6.4.1.2 6.3.4.14] | 30.87 | 39.35 | 43.8 | 8.04 | 7 | 9.55 | 1.91 | 1.63 | 1.86 |  |
| CL8437.Contig2_All | K01961 acetyl-CoA carboxylase, biotin carboxylase subunit [EC:6.4.1.2 6.3.4.14] | 5.08 | 6.27 | 5.65 | 3.87 | 5.11 | 4.7 | 0.37 | 0.23 | 0.35 |  |
| CL14917.Contig1_All | K02160 acetyl-CoA carboxylase biotin carboxyl carrier protein | 32.45 | 49.49 | 35.77 | 8.97 | 7.21 | 11.52 | 2.28 | 1.53 | 2.43 |  |
| CL14917.Contig2_All | K02160 acetyl-CoA carboxylase biotin carboxyl carrier protein | 36.71 | 45.87 | 37.34 | 8.76 | 7.61 | 9.14 | 1.95 | 2.15 | 2.58 |  |
| CL6718.Contig4_All | K02160 acetyl-CoA carboxylase biotin carboxyl carrier protein | 0.53 | 0.18 | 0 | 0.53 | 0.16 | 0 | 3.23 | 3.21 | 2 |  |
| CL750.Contig1_All | K02160 acetyl-CoA carboxylase biotin carboxyl carrier protein | 4.03 | 0.96 | 6.06 | 0.45 | 0.82 | 0.19 | 1.14 | 0.1 | 0 |  |
| CL750.Contig2_All | K02160 acetyl-CoA carboxylase biotin carboxyl carrier protein | 7.36 | 4.41 | 8.68 | 2.09 | 1.52 | 0.96 | 1.81 | 1.22 | 0.59 |  |
| CL750.Contig6_All | K02160 acetyl-CoA carboxylase biotin carboxyl carrier protein | 4.22 | 3.33 | 2.43 | 1.09 | 1.04 | 0.41 | 0.52 | 0.61 | 0.6 |  |
| Unigene23603_All | K02160 acetyl-CoA carboxylase biotin carboxyl carrier protein | 48.49 | 46.97 | 52.9 | 19.72 | 20.34 | 19.55 | 9.97 | 5.95 | 6.1 |  |
| CL3430.Contig1_All | K01853 cycloartenol synthase [EC:5.4.99.8] | 1.45 | 2.47 | 1.67 | 0.16 | 0.12 | 0.4 | 0 | 0 | 0.21 |  |
| Unigene46609_All | K01853 cycloartenol synthase [EC:5.4.99.8] | 1.61 | 3.73 | 2.39 | 1.06 | 0.31 | 2.75 | 0.67 | 0.69 | 0.18 |  |
| Unigene5575_All | K01853 cycloartenol synthase [EC:5.4.99.8] | 8.26 | 10.92 | 16.39 | 0.63 | 8.34 | 0.56 | 2.55 | 0.05 | 0.03 |  |
| Unigene7066_All | K01853 cycloartenol synthase [EC:5.4.99.8] | 3.08 | 3.89 | 3.44 | 2.34 | 1.36 | 2.95 | 0.31 | 0.88 | 0.43 |  |
| Unigene81834_All | K01853 cycloartenol synthase [EC:5.4.99.8] | 1.93 | 2.93 | 1.78 | 2.21 | 2.24 | 1.67 | 0.65 | 1.45 | 0.87 |  |
| Unigene9779_All | K01853 cycloartenol synthase [EC:5.4.99.8] | 8.35 | 10.76 | 11.7 | 6.47 | 7.33 | 6.48 | 2.56 | 1.62 | 2.38 |  |
| CL2791.Contig1_All | K00999 CDP-diacylglycerol--inositol 3-phosphatidyltransferase [EC:2.7.8.11] | 0.08 | 0.07 | 0 | 0 | 0.08 | 0.05 | 1.14 | 0.06 | 0.68 |  |
| Unigene591_All | K00999 CDP-diacylglycerol--inositol 3-phosphatidyltransferase [EC:2.7.8.11] | 12.83 | 18.29 | 15.34 | 9.61 | 8.11 | 6.59 | 5.5 | 6.77 | 4.95 |  |
| Unigene7079_All | K00999 CDP-diacylglycerol--inositol 3-phosphatidyltransferase [EC:2.7.8.11] | 0.48 | 0.57 | 0.39 | 0.22 | 0.38 | 0.65 | 1.88 | 2.21 | 3.8 |  |
| CL8513.Contig2_All | K00981 phosphatidate cytidylyltransferase [EC:2.7.7.41] | 0.23 | 0.38 | 0.07 | 0.1 | 0.14 | 0.34 | 1.58 | 1.08 | 0.94 |  |
| CL9810.Contig2_All | K00981 phosphatidate cytidylyltransferase [EC:2.7.7.41] | 2.84 | 2.86 | 4.29 | 3.12 | 2.06 | 3.76 | 9.28 | 9.87 | 8.68 |  |
| Unigene43733_All | K00981 phosphatidate cytidylyltransferase [EC:2.7.7.41] | 7.23 | 9.85 | 7.57 | 3.18 | 4.24 | 3.21 | 2 | 1.29 | 1.31 |  |
| Unigene21334_All | K15404 aldehyde decarbonylase [EC:4.1.99.5] | 0.39 | 0.87 | 0.33 | 0 | 0 | 0.11 | 0 | 0 | 0 |  |
| Unigene34375_All | K15404 aldehyde decarbonylase [EC:4.1.99.5] | 0.56 | 0.77 | 0.4 | 0 | 0 | 0 | 0 | 0 | 0 |  |
| Unigene1069_All | K04710 ceramide synthetase [EC:2.3.1.24] | 17.48 | 23.12 | 17.2 | 15.43 | 13.28 | 12.47 | 9.64 | 7.1 | 10.41 |  |
| Unigene36741_All | K04710 ceramide synthetase [EC:2.3.1.24] | 3.45 | 4.49 | 3.02 | 0.57 | 0.6 | 0.54 | 0.44 | 0.35 | 0.25 |  |
| Unigene37311_All | K04710 ceramide synthetase [EC:2.3.1.24] | 7.01 | 9.32 | 8.93 | 5.85 | 6.1 | 4.51 | 3.44 | 2.61 | 2.69 |  |
| CL2726.Contig2_All | K04628 ceramide galactosyltransferase [EC:2.4.1.47] | 46.13 | 44.35 | 46.82 | 6.71 | 8.1 | 9.62 | 0.7 | 0.16 | 0.18 |  |
| CL2726.Contig3_All | K04628 ceramide galactosyltransferase [EC:2.4.1.47] | 7.15 | 6.04 | 7.49 | 1.21 | 1.44 | 1.72 | 1.18 | 1.19 | 1.09 |  |
| CL2726.Contig4_All | K04628 ceramide galactosyltransferase [EC:2.4.1.47] | 14.3 | 16.64 | 12.17 | 15.63 | 15.67 | 11.11 | 5.04 | 5.15 | 8.06 |  |
| Unigene6167_All | K04628 ceramide galactosyltransferase [EC:2.4.1.47] | 1.61 | 2.24 | 2.26 | 0.4 | 0.33 | 0.8 | 0.85 | 1.56 | 1.22 |  |
| CL1012.Contig1_All | K00866 choline kinase [EC:2.7.1.32] | 13.76 | 14.58 | 13.86 | 13.13 | 11.93 | 9.99 | 35.25 | 52.92 | 32.2 |  |
| CL1012.Contig2_All | K00866 choline kinase [EC:2.7.1.32] | 5.45 | 7.44 | 6.91 | 4.37 | 5.51 | 3.76 | 16.06 | 18.3 | 16.99 |  |
| CL1012.Contig3_All | K00866 choline kinase [EC:2.7.1.32] | 4.56 | 4.87 | 4.15 | 4.88 | 3.81 | 4.03 | 10.8 | 18.9 | 10.65 |  |
| CL10794.Contig2_All | K00866 choline kinase [EC:2.7.1.32] | 2.32 | 2.25 | 2.9 | 4.22 | 3.45 | 2.33 | 0.71 | 0.78 | 0.18 |  |
| CL6205.Contig10_All | Caleosin/embryo-specific protein/Ca+2-binding EF hand protein | 382.73 | 418 | 506.82 | 655.64 | 735.15 | 628.6 | 1385.68 | 976.72 | 1455.59 |  |
| CL6205.Contig9_All | Caleosin/embryo-specific protein/Ca+2-binding EF hand protein | 0.1 | 0.86 | 0.49 | 1.98 | 0.64 | 3.08 | 2 | 0.63 | 1.76 |  |
| CL6205.Contig8_All | Caleosin/embryo-specific protein/Ca+2-binding EF hand protein | 15.13 | 15.22 | 14.56 | 16.49 | 26.24 | 14.39 | 50.29 | 67.06 | 57.15 |  |
| CL6205.Contig7_All | Caleosin/embryo-specific protein/Ca+2-binding EF hand protein | 0.73 | 0.83 | 0.72 | 1.13 | 3.61 | 1.34 | 0 | 0.38 | 0.93 |  |
| CL10817.Contig2_All | K07418 cytochrome P450 family 2 subfamily J [EC:1.14.14.1] | 5.69 | 10.22 | 1.83 | 1.5 | 1.2 | 1.3 | 0.03 | 0 | 0 |  |
| CL11313.Contig1_All | K07418 cytochrome P450 family 2 subfamily J [EC:1.14.14.1] | 0.05 | 0.03 | 0 | 0 | 0.81 | 0 | 466.06 | 697.34 | 3.34 |  |
| CL11313.Contig2_All | K07418 cytochrome P450 family 2 subfamily J [EC:1.14.14.1] | 0 | 0 | 0.08 | 12.92 | 6.28 | 0.44 | 476.49 | 470.55 | 2109.46 |  |
| CL874.Contig1_All | K07418 cytochrome P450 family 2 subfamily J [EC:1.14.14.1] | 12.92 | 10.06 | 11.37 | 21.68 | 18.58 | 14.44 | 70.59 | 50.03 | 60.03 |  |
| CL874.Contig2_All | K07418 cytochrome P450 family 2 subfamily J [EC:1.14.14.1] | 0.26 | 0.03 | 0.69 | 0.03 | 0.55 | 0.3 | 35.56 | 33.83 | 51.97 |  |
| CL874.Contig3_All | K07418 cytochrome P450 family 2 subfamily J [EC:1.14.14.1] | 1.08 | 0.05 | 0.82 | 0 | 0.99 | 0.46 | 28.1 | 30.23 | 48.7 |  |
| Unigene19258_All | K07418 cytochrome P450 family 2 subfamily J [EC:1.14.14.1] | 12.79 | 10.62 | 11.04 | 19.72 | 19.12 | 13.81 | 54.53 | 47.56 | 54.1 |  |
| Unigene19449_All | K07418 cytochrome P450 family 2 subfamily J [EC:1.14.14.1] | 0.49 | 0.73 | 0.58 | 2.01 | 0.67 | 0.98 | 26.66 | 38.63 | 65.06 |  |
| Unigene37292_All | K07418 cytochrome P450 family 2 subfamily J [EC:1.14.14.1] | 0 | 0.1 | 0.21 | 0.44 | 0.09 | 0.15 | 1.49 | 0.91 | 3.22 |  |
| Unigene40218_All | K07418 cytochrome P450 family 2 subfamily J [EC:1.14.14.1] | 0 | 0.12 | 0.06 | 1.01 | 0.42 | 0.39 | 2.66 | 0.43 | 2.79 |  |
| Unigene45448_All | K07418 cytochrome P450 family 2 subfamily J [EC:1.14.14.1] | 0 | 0 | 0 | 0 | 0 | 0 | 6.38 | 8.63 | 3.64 |  |
| Unigene6415_All | K07418 cytochrome P450 family 2 subfamily J [EC:1.14.14.1] | 0.67 | 1.15 | 0.91 | 1.3 | 0.82 | 1.41 | 2.07 | 3.66 | 5.74 |  |
| Unigene16781_All | K05917 sterol 14-demethylase [EC:1.14.13.70] | 80.62 | 83.24 | 87.08 | 64.03 | 61.22 | 51.42 | 30.3 | 26.14 | 27.63 |  |
| Unigene16366_All | K15398 fatty acid omega-hydroxylase [EC:1.14.-.-] | 8.26 | 19.42 | 11.44 | 0.58 | 0.04 | 0.4 | 0.22 | 0.02 | 0.25 |  |
| Unigene34331_All | K15402 fatty acid omega-hydroxylase [EC:1.14.-.-] | 7.93 | 9.86 | 6.34 | 4.24 | 3.1 | 3.27 | 0 | 0 | 0 |  |
| CL8836.Contig1_All | K16818 phospholipase A1 [EC:3.1.1.32] | 6.26 | 7.25 | 6.22 | 0.49 | 2.83 | 0.95 | 1.03 | 2.26 | 0.14 |  |
| Unigene20323_All | K16818 phospholipase A1 [EC:3.1.1.32] | 0.18 | 0.07 | 0.21 | 0.65 | 0.64 | 0.14 | 0 | 0 | 0 |  |
| Unigene34163_All | K16818 phospholipase A1 [EC:3.1.1.32] | 4.96 | 4.45 | 4.06 | 1.07 | 1.71 | 1.37 | 0 | 0 | 0 |  |
| CL6160.Contig2_All | K11155 diacylglycerol O-acyltransferase 1 [EC:2.3.1.20 2.3.1.75 2.3.1.76] | 2.73 | 4.95 | 2.44 | 4.14 | 5.1 | 3.99 | 7.68 | 10.06 | 8.9 |  |
| CL103.Contig1_All | K09480 digalactosyldiacylglycerol synthase [EC:2.4.1.241] | 7.15 | 7.62 | 6.72 | 7.46 | 5.71 | 4.89 | 1.36 | 1.11 | 2.96 |  |
| CL103.Contig2_All | K09480 digalactosyldiacylglycerol synthase [EC:2.4.1.241] | 2.67 | 4.2 | 3.57 | 4.74 | 4.38 | 2.31 | 0.81 | 1.44 | 0.68 |  |
| Unigene49020_All | K09480 digalactosyldiacylglycerol synthase [EC:2.4.1.241] | 7.86 | 6.5 | 6.06 | 6.78 | 5.42 | 3.98 | 1.44 | 2.14 | 2.44 |  |
| CL4719.Contig1_All | K00901 diacylglycerol kinase (ATP) [EC:2.7.1.107] | 7.05 | 6.29 | 4.35 | 13.1 | 7.98 | 7.5 | 5.7 | 25.58 | 17.14 |  |
| CL6390.Contig2_All | K00901 diacylglycerol kinase (ATP) [EC:2.7.1.107] | 0.09 | 0.05 | 0 | 0 | 0 | 0 | 1.16 | 1.76 | 0.2 |  |
| CL9036.Contig1_All | K00901 diacylglycerol kinase (ATP) [EC:2.7.1.107] | 43.76 | 44.18 | 38.32 | 30.28 | 26.73 | 23.51 | 17.43 | 11.69 | 16.54 |  |
| CL9036.Contig3_All | K00901 diacylglycerol kinase (ATP) [EC:2.7.1.107] | 23.16 | 25.36 | 20.27 | 15.79 | 13.32 | 10.43 | 5.12 | 2.96 | 4.7 |  |
| Unigene37120_All | K00901 diacylglycerol kinase (ATP) [EC:2.7.1.107] | 2.26 | 2.84 | 1.94 | 1.62 | 1.58 | 1.37 | 4.83 | 5.55 | 4.95 |  |
| Unigene37255_All | K00901 diacylglycerol kinase (ATP) [EC:2.7.1.107] | 12.5 | 12.15 | 13.92 | 7.71 | 7.17 | 6.77 | 3.34 | 2.76 | 4.56 |  |
| Unigene742_All | K00901 diacylglycerol kinase (ATP) [EC:2.7.1.107] | 19.11 | 21.76 | 21.11 | 20.48 | 17.15 | 13.17 | 3.83 | 3.49 | 5.19 |  |
| CL8255.Contig1_All | K09828 Delta24-sterol reductase [EC:1.3.1.72 1.3.1.-] | 55.72 | 49.43 | 48.79 | 22.89 | 19.97 | 22.07 | 18.3 | 18.58 | 12.1 |  |
| CL8255.Contig2_All | K09828 Delta24-sterol reductase [EC:1.3.1.72 1.3.1.-] | 62.68 | 59.42 | 59.07 | 28.25 | 23.35 | 23.8 | 19.92 | 22.02 | 16.65 |  |
| Unigene27021_All | K00213 7-dehydrocholesterol reductase [EC:1.3.1.21] | 28.33 | 31.99 | 27.24 | 17.29 | 16.41 | 15.58 | 12.44 | 7.26 | 13.09 |  |
| CL16386.Contig1_All | K00559 sterol 24-C-methyltransferase [EC:2.1.1.41] | 43.68 | 50.7 | 46.32 | 22.4 | 18.22 | 18.42 | 4.92 | 4.07 | 5.44 |  |
| CL16386.Contig2_All | K00559 sterol 24-C-methyltransferase [EC:2.1.1.41] | 1.15 | 3.02 | 1.73 | 0.32 | 0 | 0.56 | 0.36 | 0 | 0.47 |  |
| CL289.Contig1_All | K18693 diacylglycerol diphosphate phosphatase / phosphatidate phosphatase [EC:3.1.3.81 3.1.3.4] | 1.47 | 2.36 | 2.57 | 1.24 | 2.85 | 1.75 | 10.68 | 5.47 | 5.15 |  |
| CL289.Contig2_All | K18693 diacylglycerol diphosphate phosphatase / phosphatidate phosphatase [EC:3.1.3.81 3.1.3.4] | 0.15 | 0 | 0.34 | 1.3 | 0.16 | 1.09 | 1.7 | 6.33 | 9.36 |  |
| Unigene5615_All | K18693 diacylglycerol diphosphate phosphatase / phosphatidate phosphatase [EC:3.1.3.81 3.1.3.4] | 0.12 | 0.5 | 0.22 | 0.59 | 0.11 | 0.37 | 0 | 0 | 0 |  |
| Unigene56159_All | K18693 diacylglycerol diphosphate phosphatase / phosphatidate phosphatase [EC:3.1.3.81 3.1.3.4] | 0.83 | 0.79 | 0.67 | 0.38 | 0 | 0.25 | 0 | 0 | 0 |  |
| CL5466.Contig2_All | K00208 enoyl-[acyl-carrier protein] reductase I [EC:1.3.1.9 1.3.1.10] | 18.07 | 19.01 | 18.47 | 3.7 | 2.43 | 2.45 | 0 | 1.55 | 1.79 |  |
| CL7296.Contig2_All | K00208 enoyl-[acyl-carrier protein] reductase I [EC:1.3.1.9 1.3.1.10] | 30.18 | 68.6 | 63.06 | 4.83 | 3.35 | 10.46 | 0.15 | 0.04 | 0 |  |
| CL7296.Contig3_All | K00208 enoyl-[acyl-carrier protein] reductase I [EC:1.3.1.9 1.3.1.10] | 44.1 | 17.27 | 20.16 | 3.58 | 4.29 | 3.23 | 0 | 0 | 0 |  |
| Unigene16292_All | K01824 cholestenol Delta-isomerase [EC:5.3.3.5] | 9.53 | 10.14 | 8.99 | 6.84 | 4.13 | 3.51 | 1.03 | 0.39 | 1.18 |  |
| CL3941.Contig2_All | K10258 very-long-chain enoyl-CoA reductase [EC:1.3.1.93] | 1.55 | 3.07 | 3.01 | 3.72 | 5.95 | 1.7 | 17.71 | 23.31 | 12.2 |  |
| CL3941.Contig3_All | K10258 very-long-chain enoyl-CoA reductase [EC:1.3.1.93] | 10.67 | 13.49 | 15.53 | 16.47 | 17.54 | 12.89 | 48.8 | 43.82 | 35.8 |  |
| Unigene30380_All | K10258 very-long-chain enoyl-CoA reductase [EC:1.3.1.93] | 36.19 | 37.18 | 29.64 | 17.02 | 14.76 | 16.26 | 6.67 | 5.3 | 9.3 |  |
| CL1343.Contig1_All | K08726 soluble epoxide hydrolase / lipid-phosphate phosphatase [EC:3.3.2.10 3.1.3.76] | 7.46 | 4.96 | 5.88 | 5.66 | 4.43 | 5.17 | 48.54 | 61.75 | 63.12 |  |
| CL1996.Contig5_All | K08726 soluble epoxide hydrolase / lipid-phosphate phosphatase [EC:3.3.2.10 3.1.3.76] | 5.14 | 8.65 | 5.54 | 1.91 | 1.46 | 1.87 | 4.52 | 2.97 | 2.64 |  |
| CL8879.Contig1_All | K08726 soluble epoxide hydrolase / lipid-phosphate phosphatase [EC:3.3.2.10 3.1.3.76] | 11.94 | 9.62 | 10 | 12.54 | 7.88 | 7.11 | 90.5 | 107.02 | 119.12 |  |
| CL8879.Contig2_All | K08726 soluble epoxide hydrolase / lipid-phosphate phosphatase [EC:3.3.2.10 3.1.3.76] | 1.54 | 0.85 | 1.73 | 0.65 | 0.73 | 0.61 | 3.35 | 3.51 | 2.75 |  |
| Unigene16719_All | K08726 soluble epoxide hydrolase / lipid-phosphate phosphatase [EC:3.3.2.10 3.1.3.76] | 39.4 | 40.22 | 43.23 | 14.77 | 7.17 | 9.48 | 5.72 | 2.37 | 4.61 |  |
| Unigene16720_All | K08726 soluble epoxide hydrolase / lipid-phosphate phosphatase [EC:3.3.2.10 3.1.3.76] | 47.77 | 54.62 | 54.78 | 16.61 | 10.07 | 12.46 | 6.57 | 5.63 | 3.42 |  |
| Unigene24563_All | K08726 soluble epoxide hydrolase / lipid-phosphate phosphatase [EC:3.3.2.10 3.1.3.76] | 9.76 | 10.72 | 9.99 | 49.31 | 41.73 | 25.51 | 194.83 | 239 | 292.63 |  |
| Unigene44412_All | K08726 soluble epoxide hydrolase / lipid-phosphate phosphatase [EC:3.3.2.10 3.1.3.76] | 11.44 | 13.8 | 15.44 | 56.72 | 69.08 | 37.34 | 296.85 | 436.85 | 418.95 |  |
| Unigene44413_All | K08726 soluble epoxide hydrolase / lipid-phosphate phosphatase [EC:3.3.2.10 3.1.3.76] | 2.24 | 10.94 | 8.97 | 22.92 | 35.11 | 21.45 | 202.56 | 320.74 | 254.69 |  |
| CL12825.Contig1_All | K00993 ethanolaminephosphotransferase [EC:2.7.8.1] | 30.02 | 32.76 | 25.65 | 18.67 | 16.55 | 14.84 | 7.77 | 9.81 | 9.29 |  |
| CL2636.Contig1_All | K10256 omega-6 fatty acid desaturase / acyl-lipid omega-6 desaturase (Delta-12 desaturase) [EC:1.14.19.6 1.14.19.22] | 2.26 | 2.87 | 4.75 | 1.92 | 0 | 1.18 | 0.13 | 0.09 | 0 |  |
| CL2636.Contig2_All | K10256 omega-6 fatty acid desaturase / acyl-lipid omega-6 desaturase (Delta-12 desaturase) [EC:1.14.19.6 1.14.19.22] | 2.56 | 3.16 | 6.69 | 1.5 | 0.07 | 0.64 | 0.08 | 0 | 0 |  |
| CL2636.Contig3_All | K10256 omega-6 fatty acid desaturase / acyl-lipid omega-6 desaturase (Delta-12 desaturase) [EC:1.14.19.6 1.14.19.22] | 72.38 | 103.25 | 150.12 | 47.61 | 27.48 | 65.45 | 1.58 | 0.71 | 1.25 |  |
| CL2636.Contig4_All | K10256 omega-6 fatty acid desaturase / acyl-lipid omega-6 desaturase (Delta-12 desaturase) [EC:1.14.19.6 1.14.19.22] | 785.17 | 405.49 | 665.9 | 332.46 | 217.02 | 329.44 | 3.95 | 1.36 | 2.72 |  |
| CL6171.Contig1_All | K10256 omega-6 fatty acid desaturase / acyl-lipid omega-6 desaturase (Delta-12 desaturase) [EC:1.14.19.6 1.14.19.22] | 1.26 | 2.41 | 3.41 | 0.54 | 0.79 | 1.12 | 0 | 0 | 0.12 |  |
| CL6171.Contig2_All | K10256 omega-6 fatty acid desaturase / acyl-lipid omega-6 desaturase (Delta-12 desaturase) [EC:1.14.19.6 1.14.19.22] | 2.84 | 0.4 | 3.72 | 4.16 | 7.11 | 2.06 | 0 | 0.22 | 0.02 |  |
| CL6171.Contig3_All | K10256 omega-6 fatty acid desaturase / acyl-lipid omega-6 desaturase (Delta-12 desaturase) [EC:1.14.19.6 1.14.19.22] | 0.48 | 1.38 | 2.32 | 2.16 | 1.89 | 4.61 | 0.12 | 0.06 | 0.26 |  |
| CL6171.Contig4_All | K10256 omega-6 fatty acid desaturase / acyl-lipid omega-6 desaturase (Delta-12 desaturase) [EC:1.14.19.6 1.14.19.22] | 3.1 | 2.24 | 11.82 | 10.05 | 11.69 | 13.12 | 0.4 | 0.17 | 0.16 |  |
| Unigene37303_All | K10256 omega-6 fatty acid desaturase / acyl-lipid omega-6 desaturase (Delta-12 desaturase) [EC:1.14.19.6 1.14.19.22] | 40.07 | 36.53 | 40.77 | 40.67 | 35.02 | 36.43 | 1.7 | 1.1 | 1.44 |  |
| CL2686.Contig5_All | endoplasmic reticulum; K10257 acyl-lipid omega-3 desaturase [EC:1.14.19.25 1.14.19.35 | 54.5 | 270.48 | 0.66 | 269.82 | 108.96 | 629.84 | 0 | 0.81 | 0.71 |  |
| CL2686.Contig3_All | endoplasmic reticulum; K10257 acyl-lipid omega-3 desaturase [EC:1.14.19.25 1.14.19.35 | 0.27 | 0.95 | 1.09 | 0.09 | 0.61 | 0.09 | 0.1 | 0.01 | 0.01 |  |
| CL2686.Contig8_All | endoplasmic reticulum; K10257 acyl-lipid omega-3 desaturase [EC:1.14.19.25 1.14.19.35 | 410.47 | 311.06 | 526.27 | 32.91 | 98.65 | 0.92 | 1.56 | 0.32 | 0.42 |  |
| CL2686.Contig6_All | endoplasmic reticulum; K10257 acyl-lipid omega-3 desaturase [EC:1.14.19.25 1.14.19.35 | 439.22 | 520.31 | 481.17 | 262.84 | 177.35 | 565.75 | 1.76 | 0.39 | 0.61 |  |
| CL2686.Contig7_All | endoplasmic reticulum; K10257 acyl-lipid omega-3 desaturase [EC:1.14.19.25 1.14.19.35 | 0.42 | 0.79 | 1.42 | 0.41 | 0 | 0.05 | 0.15 | 0.05 | 0 |  |
| CL13349.Contig2_All | chloroplastic; K10257 acyl-lipid omega-3 desaturase [EC:1.14.19.25 1.14.19.35 1.14.19.36] | 10.92 | 9.05 | 9.93 | 9.3 | 9.31 | 8.51 | 0.46 | 0.32 | 0.41 |  |
| CL13349.Contig1_All | chloroplastic; K10257 acyl-lipid omega-3 desaturase [EC:1.14.19.25 1.14.19.35 1.14.19.36] | 7.02 | 7.37 | 9.24 | 5.87 | 7.51 | 6.72 | 0.3 | 0 | 0.11 |  |
| Unigene34203_All | chloroplastic; K10257 acyl-lipid omega-3 desaturase [EC:1.14.19.25 1.14.19.35 | 50.53 | 30.32 | 43.45 | 22.37 | 38.39 | 33.85 | 0.01 | 0.01 | 0.01 |  |
| Unigene34202_All | chloroplastic; K10257 acyl-lipid omega-3 desaturase [EC:1.14.19.25 1.14.19.35 | 46.41 | 29.21 | 44.17 | 19.22 | 35.19 | 32.51 | 0.04 | 0.01 | 0.01 |  |
| CL11819.Contig1_All | K13356 alcohol-forming fatty acyl-CoA reductase [EC:1.2.1.84] | 3.02 | 1.98 | 1.77 | 11.03 | 10.37 | 10.16 | 0.12 | 0 | 0 |  |
| CL15198.Contig1_All | K13356 alcohol-forming fatty acyl-CoA reductase [EC:1.2.1.84] | 0.05 | 0.37 | 0.27 | 4.49 | 2.79 | 0.85 | 0.03 | 0 | 0 |  |
| CL15198.Contig2_All | K13356 alcohol-forming fatty acyl-CoA reductase [EC:1.2.1.84] | 0.87 | 1.43 | 0.64 | 0.52 | 0.19 | 0.42 | 0.04 | 0 | 0 |  |
| CL244.Contig2_All | K13356 alcohol-forming fatty acyl-CoA reductase [EC:1.2.1.84] | 40.09 | 36.53 | 43.64 | 46.8 | 35.22 | 27.74 | 0 | 0 | 0 |  |
| CL549.Contig3_All | K13356 alcohol-forming fatty acyl-CoA reductase [EC:1.2.1.84] | 1.32 | 1.14 | 0.27 | 0.26 | 0.1 | 0.29 | 0.11 | 0.04 | 0.04 |  |
| CL14443.Contig1_All | K10782 fatty acyl-ACP thioesterase A [EC:3.1.2.14] | 44.91 | 45.88 | 46.39 | 24 | 18.52 | 21.27 | 13.51 | 8.8 | 12.81 |  |
| Unigene1453_All | K10781 fatty acyl-ACP thioesterase B [EC:3.1.2.14 3.1.2.21] | 0.04 | 0.58 | 0 | 0.33 | 0.25 | 0.08 | 0 | 0 | 0 |  |
| Unigene26774_All | K10781 fatty acyl-ACP thioesterase B [EC:3.1.2.14 3.1.2.21] | 69.36 | 83.51 | 73.45 | 40.97 | 40.73 | 44.65 | 31.01 | 38.44 | 27.91 |  |
| CL13056.Contig1_All | K00801 farnesyl-diphosphate farnesyltransferase [EC:2.5.1.21] | 197.71 | 189.15 | 171.04 | 2.69 | 2.14 | 3.15 | 1.2 | 0.09 | 1.5 |  |
| CL14445.Contig2_All | K00801 farnesyl-diphosphate farnesyltransferase [EC:2.5.1.21] | 0.43 | 1.25 | 0.39 | 0.96 | 0.95 | 0.54 | 0 | 0 | 0 |  |
| CL15743.Contig3_All | K00801 farnesyl-diphosphate farnesyltransferase [EC:2.5.1.21] | 0.99 | 0.63 | 0.5 | 0.64 | 0.16 | 0.27 | 0 | 0 | 0 |  |
| CL1637.Contig2_All | K00801 farnesyl-diphosphate farnesyltransferase [EC:2.5.1.21] | 1.27 | 2.6 | 1.26 | 1.12 | 0.72 | 0.56 | 0 | 0 | 0 |  |
| CL2944.Contig2_All | K00801 farnesyl-diphosphate farnesyltransferase [EC:2.5.1.21] | 0.49 | 3.85 | 1.76 | 19.54 | 16.58 | 8.04 | 5.22 | 4 | 0 |  |
| CL2944.Contig3_All | K00801 farnesyl-diphosphate farnesyltransferase [EC:2.5.1.21] | 7.05 | 3.92 | 6.04 | 41.79 | 34.28 | 16.03 | 0.35 | 0.16 | 3.58 |  |
| CL2992.Contig1_All | K00801 farnesyl-diphosphate farnesyltransferase [EC:2.5.1.21] | 0 | 0 | 0 | 0.05 | 0.1 | 0 | 1.82 | 0.49 | 1.49 |  |
| CL2992.Contig2_All | K00801 farnesyl-diphosphate farnesyltransferase [EC:2.5.1.21] | 0 | 0 | 0 | 0.25 | 0 | 0 | 1.38 | 0.09 | 1.12 |  |
| CL2992.Contig3_All | K00801 farnesyl-diphosphate farnesyltransferase [EC:2.5.1.21] | 0.24 | 0 | 0.47 | 1.3 | 1.52 | 1.07 | 1.73 | 3.58 | 3.62 |  |
| CL9303.Contig1_All | K00801 farnesyl-diphosphate farnesyltransferase [EC:2.5.1.21] | 10.26 | 15.97 | 9.94 | 10.99 | 6.86 | 8.69 | 2.54 | 1.1 | 2.89 |  |
| CL9303.Contig2_All | K00801 farnesyl-diphosphate farnesyltransferase [EC:2.5.1.21] | 11.1 | 9.96 | 11.45 | 8.24 | 7.11 | 6.66 | 0.83 | 1.91 | 1.05 |  |
| CL9303.Contig3_All | K00801 farnesyl-diphosphate farnesyltransferase [EC:2.5.1.21] | 9.73 | 15.96 | 10.05 | 9.57 | 7.27 | 8.67 | 2.87 | 0.8 | 3.24 |  |
| CL9303.Contig4_All | K00801 farnesyl-diphosphate farnesyltransferase [EC:2.5.1.21] | 9.53 | 12.29 | 9.13 | 7.09 | 6.57 | 7.34 | 1.19 | 1.55 | 1.05 |  |
| Unigene16854_All | K00801 farnesyl-diphosphate farnesyltransferase [EC:2.5.1.21] | 1.24 | 2.21 | 0.72 | 0.62 | 1.92 | 0.66 | 0 | 0 | 0 |  |
| Unigene20575_All | K00801 farnesyl-diphosphate farnesyltransferase [EC:2.5.1.21] | 74.84 | 64.43 | 74.99 | 126 | 89.23 | 84.15 | 0.07 | 0 | 0 |  |
| Unigene24805_All | K00801 farnesyl-diphosphate farnesyltransferase [EC:2.5.1.21] | 0.04 | 0.18 | 0.09 | 1.01 | 1.3 | 1.01 | 0 | 0 | 0 |  |
| Unigene30788_All | K00801 farnesyl-diphosphate farnesyltransferase [EC:2.5.1.21] | 31.54 | 25.23 | 28.59 | 2.81 | 1.41 | 12.4 | 0 | 0 | 0 |  |
| Unigene34307_All | K00801 farnesyl-diphosphate farnesyltransferase [EC:2.5.1.21] | 34.29 | 49.1 | 10.86 | 13.54 | 9.12 | 11.44 | 0.1 | 0 | 0.07 |  |
| Unigene41108_All | K00801 farnesyl-diphosphate farnesyltransferase [EC:2.5.1.21] | 69.32 | 47.58 | 61.73 | 140.79 | 103.8 | 67.35 | 0.1 | 0.03 | 0.03 |  |
| Unigene44978_All | K00801 farnesyl-diphosphate farnesyltransferase [EC:2.5.1.21] | 0.06 | 0.26 | 0.31 | 0.12 | 0.54 | 0.12 | 0 | 0 | 0 |  |
| CL1452.Contig1_All | K07407 alpha-galactosidase [EC:3.2.1.22] | 5.72 | 4.87 | 1.93 | 1.32 | 1.47 | 0.46 | 0.15 | 0 | 0.42 |  |
| CL1452.Contig2_All | K07407 alpha-galactosidase [EC:3.2.1.22] | 0.94 | 2.76 | 1.19 | 0.5 | 0.78 | 0.79 | 0 | 0.15 | 0 |  |
| CL4714.Contig2_All | K07407 alpha-galactosidase [EC:3.2.1.22] | 17.15 | 14.8 | 21.25 | 10.82 | 9.78 | 7.6 | 3.93 | 5.62 | 3.94 |  |
| CL8793.Contig2_All | K07407 alpha-galactosidase [EC:3.2.1.22] | 36.2 | 40.16 | 33.26 | 153.89 | 133.91 | 78.8 | 7.66 | 10.66 | 10.35 |  |
| CL8793.Contig3_All | K07407 alpha-galactosidase [EC:3.2.1.22] | 31.43 | 37.77 | 32.1 | 151.24 | 130.9 | 78.44 | 7.48 | 11.51 | 9.78 |  |
| Unigene1060_All | K07407 alpha-galactosidase [EC:3.2.1.22] | 25.59 | 26.89 | 26.81 | 37.22 | 33.29 | 22.33 | 13.76 | 11.75 | 9.69 |  |
| CL11012.Contig2_All | K17108 non-lysosomal glucosylceramidase [EC:3.2.1.45] | 7.28 | 7.76 | 7.44 | 5.29 | 3.94 | 4.3 | 0.67 | 1.45 | 0.92 |  |
| CL5509.Contig1_All | K17108 non-lysosomal glucosylceramidase [EC:3.2.1.45] | 0.16 | 0.14 | 0.07 | 0.43 | 0.21 | 0 | 0.61 | 0.55 | 0.8 |  |
| CL5509.Contig2_All | K17108 non-lysosomal glucosylceramidase [EC:3.2.1.45] | 2.75 | 3.47 | 3.63 | 1.27 | 1.24 | 1.34 | 0.47 | 1.3 | 1.25 |  |
| CL11992.Contig1_All | K18696 glycerophosphodiester phosphodiesterase [EC:3.1.4.46] | 20.8 | 6.93 | 8.5 | 21.87 | 17.65 | 14.02 | 0.81 | 0.08 | 0.67 |  |
| CL11992.Contig2_All | K18696 glycerophosphodiester phosphodiesterase [EC:3.1.4.46] | 5.01 | 0.2 | 2.8 | 25.69 | 43.35 | 21.87 | 22.86 | 16.47 | 49.24 |  |
| CL11992.Contig4_All | K18696 glycerophosphodiester phosphodiesterase [EC:3.1.4.46] | 4.64 | 0.08 | 2.49 | 24.92 | 43.82 | 21.02 | 22.61 | 14.73 | 47.48 |  |
| CL6734.Contig3_All | K18696 glycerophosphodiester phosphodiesterase [EC:3.1.4.46] | 0.48 | 0.32 | 2.08 | 0.43 | 0.62 | 0 | 0 | 0 | 0 |  |
| CL6734.Contig4_All | K18696 glycerophosphodiester phosphodiesterase [EC:3.1.4.46] | 0.29 | 1.04 | 1.06 | 1.27 | 1.59 | 2.06 | 0 | 0 | 0.04 |  |
| CL6734.Contig5_All | K18696 glycerophosphodiester phosphodiesterase [EC:3.1.4.46] | 7.52 | 8.14 | 9.16 | 12.6 | 10.4 | 7.92 | 0.62 | 0.49 | 0.15 |  |
| Unigene40962_All | K18696 glycerophosphodiester phosphodiesterase [EC:3.1.4.46] | 3.2 | 1.45 | 0.91 | 3.15 | 2.67 | 1.92 | 0 | 0 | 0 |  |
| CL7829.Contig2_All | K01094 phosphatidylglycerophosphatase GEP4 [EC:3.1.3.27] | 3.16 | 3.44 | 2.54 | 0.92 | 0.56 | 0.57 | 1.82 | 2.12 | 2.98 |  |
| CL7829.Contig4_All | K01094 phosphatidylglycerophosphatase GEP4 [EC:3.1.3.27] | 0.27 | 0.16 | 0 | 0 | 0 | 0 | 0.57 | 0.25 | 0.31 |  |
| CL9384.Contig1_All | K18592 gamma-glutamyltranspeptidase / glutathione hydrolase / leukotriene-C4 hydrolase [EC:2.3.2.2 3.4.19.13 3.4.19.14] | 0.04 | 0.18 | 0.05 | 0.19 | 0.27 | 0.64 | 0 | 0.05 | 0.11 |  |
| Unigene41023_All | K18592 gamma-glutamyltranspeptidase / glutathione hydrolase / leukotriene-C4 hydrolase [EC:2.3.2.2 3.4.19.13 3.4.19.14] | 28.85 | 35.63 | 26.88 | 25.1 | 23.95 | 18.71 | 0.08 | 0.08 | 0.21 |  |
| CL14885.Contig1_All | K00864 glycerol kinase [EC:2.7.1.30] | 12.31 | 10.35 | 9.99 | 19.88 | 19.77 | 13.33 | 7.58 | 6.15 | 9.24 |  |
| CL10908.Contig1_All | K12309 beta-galactosidase [EC:3.2.1.23] | 2.05 | 1.33 | 2.33 | 0.47 | 0.31 | 0.6 | 0 | 0 | 0.04 |  |
| CL1227.Contig2_All | K12309 beta-galactosidase [EC:3.2.1.23] | 26.6 | 23.45 | 28.8 | 1.51 | 0.48 | 2.47 | 0.31 | 0.35 | 0.65 |  |
| CL12750.Contig3_All | K12309 beta-galactosidase [EC:3.2.1.23] | 2.79 | 2.62 | 3.2 | 1.65 | 1.2 | 1.83 | 0.06 | 0.06 | 0 |  |
| CL14704.Contig2_All | K12309 beta-galactosidase [EC:3.2.1.23] | 3.5 | 2.96 | 2.07 | 3.3 | 3.18 | 1.97 | 6.14 | 8.79 | 7.21 |  |
| CL16020.Contig1_All | K12309 beta-galactosidase [EC:3.2.1.23] | 7.65 | 9.3 | 5.9 | 6.5 | 4.73 | 5.99 | 0.07 | 0 | 0 |  |
| CL16020.Contig2_All | K12309 beta-galactosidase [EC:3.2.1.23] | 1.94 | 2.09 | 1.98 | 1.03 | 0.56 | 1.62 | 0 | 0 | 0 |  |
| CL16020.Contig3_All | K12309 beta-galactosidase [EC:3.2.1.23] | 0.14 | 0.53 | 0.43 | 0.13 | 0 | 0 | 0 | 0.02 | 0 |  |
| CL3688.Contig1_All | K12309 beta-galactosidase [EC:3.2.1.23] | 10.21 | 9.73 | 9.86 | 7.95 | 8.41 | 6.68 | 2.89 | 2.27 | 3.21 |  |
| CL3688.Contig2_All | K12309 beta-galactosidase [EC:3.2.1.23] | 2.67 | 2.08 | 4.02 | 1.81 | 0.97 | 1.55 | 0.12 | 0.84 | 0.26 |  |
| CL3934.Contig1_All | K12309 beta-galactosidase [EC:3.2.1.23] | 5.71 | 2.51 | 11.19 | 2.03 | 1.19 | 2.12 | 0.18 | 0.16 | 0.17 |  |
| CL3934.Contig4_All | K12309 beta-galactosidase [EC:3.2.1.23] | 72.71 | 70.6 | 68.14 | 34.59 | 53.06 | 49.52 | 19.28 | 18.19 | 26.96 |  |
| CL3943.Contig2_All | K12309 beta-galactosidase [EC:3.2.1.23] | 22.55 | 28.58 | 21.72 | 10.39 | 5.66 | 10.25 | 4.41 | 3.49 | 1.21 |  |
| CL4644.Contig1_All | K12309 beta-galactosidase [EC:3.2.1.23] | 7.15 | 6.54 | 5.55 | 10.44 | 10.85 | 8.85 | 0 | 0 | 0 |  |
| CL4644.Contig2_All | K12309 beta-galactosidase [EC:3.2.1.23] | 5.85 | 5.34 | 4.21 | 10.98 | 10.55 | 9.94 | 0 | 0 | 0 |  |
| CL4644.Contig6_All | K12309 beta-galactosidase [EC:3.2.1.23] | 1.22 | 1.28 | 1.47 | 0.81 | 0.41 | 0.53 | 0 | 0 | 0 |  |
| CL4644.Contig7_All | K12309 beta-galactosidase [EC:3.2.1.23] | 8.77 | 7.91 | 7.96 | 3.76 | 4.81 | 4.2 | 0 | 0 | 0 |  |
| CL6711.Contig1_All | K12309 beta-galactosidase [EC:3.2.1.23] | 20.52 | 20.12 | 17.1 | 43.74 | 38.25 | 40.3 | 103.79 | 115.95 | 113.54 |  |
| CL711.Contig11_All | K12309 beta-galactosidase [EC:3.2.1.23] | 0.13 | 0.37 | 0.25 | 0.11 | 0.18 | 0.14 | 0.7 | 1 | 1.31 |  |
| CL711.Contig15_All | K12309 beta-galactosidase [EC:3.2.1.23] | 0.73 | 1.48 | 1.55 | 1.76 | 0.77 | 0.86 | 3.94 | 5.76 | 4.25 |  |
| CL711.Contig3_All | K12309 beta-galactosidase [EC:3.2.1.23] | 0.33 | 1.14 | 0.77 | 0.46 | 0 | 0.15 | 0.15 | 0.16 | 0 |  |
| CL8334.Contig1_All | K12309 beta-galactosidase [EC:3.2.1.23] | 200.05 | 174.73 | 183.72 | 280.61 | 269.86 | 231.8 | 0.17 | 0 | 0.03 |  |
| CL8771.Contig1_All | K12309 beta-galactosidase [EC:3.2.1.23] | 16.16 | 20.56 | 20.46 | 60.23 | 27.48 | 79.93 | 7.63 | 3.42 | 3.65 |  |
| CL8771.Contig4_All | K12309 beta-galactosidase [EC:3.2.1.23] | 3.29 | 4.38 | 3.97 | 6.86 | 3.29 | 6.62 | 2.83 | 2.08 | 1.82 |  |
| CL9116.Contig1_All | K12309 beta-galactosidase [EC:3.2.1.23] | 5.02 | 6.53 | 7.04 | 3.84 | 2.57 | 3.07 | 0.97 | 0.53 | 0.64 |  |
| CL9116.Contig2_All | K12309 beta-galactosidase [EC:3.2.1.23] | 5.32 | 6.24 | 7.09 | 3.58 | 2.93 | 3.34 | 0.75 | 0.83 | 0.66 |  |
| Unigene147_All | K12309 beta-galactosidase [EC:3.2.1.23] | 15.14 | 20.23 | 18.98 | 61.84 | 28.1 | 78.68 | 7.06 | 3.41 | 2.7 |  |
| Unigene16631_All | K12309 beta-galactosidase [EC:3.2.1.23] | 106.55 | 149.93 | 128.85 | 67.16 | 88.26 | 75.41 | 13.24 | 3.46 | 9.29 |  |
| Unigene19742_All | K12309 beta-galactosidase [EC:3.2.1.23] | 1.01 | 1.33 | 0.89 | 0.86 | 1.4 | 0.84 | 0 | 0 | 0 |  |
| Unigene23857_All | K12309 beta-galactosidase [EC:3.2.1.23] | 0.32 | 0.38 | 0.73 | 0 | 0 | 0 | 0 | 0 | 0.05 |  |
| Unigene30602_All | K12309 beta-galactosidase [EC:3.2.1.23] | 0.02 | 0.02 | 0.08 | 1.65 | 0.12 | 1 | 0.56 | 0.31 | 0.28 |  |
| Unigene33543_All | K12309 beta-galactosidase [EC:3.2.1.23] | 0.08 | 0.04 | 0.08 | 0.53 | 0.34 | 0.61 | 3.01 | 2.2 | 4.61 |  |
| Unigene33736_All | K12309 beta-galactosidase [EC:3.2.1.23] | 0.69 | 0.74 | 0.97 | 7.9 | 7.36 | 1.87 | 0.04 | 0 | 0 |  |
| Unigene34158_All | K12309 beta-galactosidase [EC:3.2.1.23] | 0.15 | 0.8 | 0.16 | 0.4 | 0.17 | 0.17 | 3.11 | 2.07 | 0.79 |  |
| Unigene34170_All | K12309 beta-galactosidase [EC:3.2.1.23] | 0.17 | 0.04 | 0 | 1.46 | 3.43 | 1.9 | 42.24 | 21.3 | 23.09 |  |
| Unigene36956_All | K12309 beta-galactosidase [EC:3.2.1.23] | 4.03 | 3.48 | 2.49 | 2.62 | 2.7 | 1.63 | 0.32 | 0.16 | 0.3 |  |
| Unigene40200_All | K12309 beta-galactosidase [EC:3.2.1.23] | 0.71 | 0.81 | 0.19 | 1.19 | 1 | 0.71 | 0 | 0 | 0 |  |
| CL12440.Contig2_All | K01126 glycerophosphoryl diester phosphodiesterase [EC:3.1.4.46] | 9.26 | 7.79 | 8.93 | 8.76 | 5.55 | 6.3 | 0.03 | 1.4 | 0.73 |  |
| CL12440.Contig3_All | K01126 glycerophosphoryl diester phosphodiesterase [EC:3.1.4.46] | 8.09 | 10.37 | 7.96 | 9.8 | 6.48 | 6.51 | 0 | 1.63 | 0.93 |  |
| CL265.Contig1_All | K01126 glycerophosphoryl diester phosphodiesterase [EC:3.1.4.46] | 0.22 | 0.98 | 0.36 | 0.28 | 0.07 | 0.28 | 0 | 0 | 0.08 |  |
| CL265.Contig3_All | K01126 glycerophosphoryl diester phosphodiesterase [EC:3.1.4.46] | 113.42 | 107.55 | 95.21 | 49.08 | 52.73 | 52.87 | 4.15 | 3.69 | 1.68 |  |
| CL265.Contig4_All | K01126 glycerophosphoryl diester phosphodiesterase [EC:3.1.4.46] | 115.26 | 113.33 | 100.9 | 50.96 | 55.7 | 50.99 | 4.59 | 4.66 | 2.51 |  |
| CL265.Contig5_All | K01126 glycerophosphoryl diester phosphodiesterase [EC:3.1.4.46] | 0.93 | 1.46 | 0.84 | 1.05 | 0.39 | 0.48 | 0.27 | 0.38 | 0.31 |  |
| CL9492.Contig1_All | K01126 glycerophosphoryl diester phosphodiesterase [EC:3.1.4.46] | 0.44 | 0.74 | 0.48 | 0.59 | 0.29 | 0.43 | 0 | 0 | 0 |  |
| CL9492.Contig4_All | K01126 glycerophosphoryl diester phosphodiesterase [EC:3.1.4.46] | 1.03 | 0.52 | 0.26 | 0.37 | 0.28 | 0.3 | 0 | 0 | 0 |  |
| CL13606.Contig3_All | K15918 D-glycerate 3-kinase [EC:2.7.1.31] | 1.28 | 1.98 | 1.22 | 2.12 | 1.23 | 1.85 | 5.44 | 2.32 | 5.33 |  |
| CL12348.Contig2_All | K00630 glycerol-3-phosphate O-acyltransferase [EC:2.3.1.15] | 1.66 | 2.44 | 2.29 | 0.75 | 0.56 | 0.6 | 0.6 | 0.29 | 0.99 |  |
| CL12348.Contig3_All | K00630 glycerol-3-phosphate O-acyltransferase [EC:2.3.1.15] | 1.88 | 2.37 | 1.74 | 0.69 | 0.79 | 1.05 | 0.49 | 0.36 | 0.44 |  |
| Unigene30381_All | K00630 glycerol-3-phosphate O-acyltransferase [EC:2.3.1.15] | 7.02 | 9.91 | 6.29 | 4.2 | 2.84 | 3.77 | 3.23 | 2.09 | 1.5 |  |
| Unigene38234_All | K00630 glycerol-3-phosphate O-acyltransferase [EC:2.3.1.15] | 0.09 | 0.06 | 0 | 9.98 | 7.55 | 2.4 | 0.03 | 0 | 0 |  |
| CL12015.Contig1_All | K00006 glycerol-3-phosphate dehydrogenase (NAD+) [EC:1.1.1.8] | 20.48 | 16.9 | 17.72 | 23.28 | 20.87 | 17.39 | 9.41 | 8.34 | 10.58 |  |
| CL12015.Contig2_All | K00006 glycerol-3-phosphate dehydrogenase (NAD+) [EC:1.1.1.8] | 19.9 | 17.31 | 15.47 | 20.58 | 17.06 | 15.11 | 8.23 | 7.86 | 9.28 |  |
| Unigene30444_All | K00006 glycerol-3-phosphate dehydrogenase (NAD+) [EC:1.1.1.8] | 13.82 | 15.03 | 14.12 | 10.21 | 7.08 | 10.51 | 1.77 | 2.84 | 3.15 |  |
| Unigene600_All | K00006 glycerol-3-phosphate dehydrogenase (NAD+) [EC:1.1.1.8] | 1.82 | 3.75 | 1.62 | 0.75 | 0.61 | 0.71 | 0 | 0.12 | 0.03 |  |
| CL6460.Contig1_All | K00432 glutathione peroxidase [EC:1.11.1.9] | 3.24 | 2.21 | 3.57 | 5.1 | 2.06 | 3.04 | 1.03 | 0.85 | 0.98 |  |
| CL6460.Contig2_All | K00432 glutathione peroxidase [EC:1.11.1.9] | 50.82 | 72.79 | 75.49 | 50.32 | 40.6 | 36.46 | 7.59 | 7.72 | 14.11 |  |
| CL6460.Contig4_All | K00432 glutathione peroxidase [EC:1.11.1.9] | 43.97 | 62.86 | 61.15 | 43.67 | 32.72 | 32.65 | 8.11 | 7.92 | 11.41 |  |
| Unigene30171_All | K00432 glutathione peroxidase [EC:1.11.1.9] | 14.84 | 19.67 | 15.46 | 10.07 | 8.11 | 7.43 | 3.97 | 3.52 | 3.29 |  |
| CL1701.Contig1_All | K02372 3-hydroxyacyl-[acyl-carrier-protein] dehydratase [EC:4.2.1.59] | 8.73 | 7.33 | 6.52 | 1.76 | 1.85 | 0.95 | 0.05 | 0.1 | 0.26 |  |
| CL1701.Contig2_All | K02372 3-hydroxyacyl-[acyl-carrier-protein] dehydratase [EC:4.2.1.59] | 7.63 | 7.03 | 7.35 | 1.29 | 1.72 | 1.96 | 1.24 | 0.42 | 0.77 |  |
| CL1701.Contig4_All | K02372 3-hydroxyacyl-[acyl-carrier-protein] dehydratase [EC:4.2.1.59] | 21.58 | 15.93 | 16.56 | 1.96 | 0.92 | 1.82 | 0.46 | 0.33 | 0.66 |  |
| CL1701.Contig5_All | K02372 3-hydroxyacyl-[acyl-carrier-protein] dehydratase [EC:4.2.1.59] | 48.33 | 40.85 | 40.68 | 14.1 | 13.2 | 13.76 | 15.35 | 11.76 | 13.23 |  |
| CL6818.Contig4_All | K10703 very-long-chain (3R)-3-hydroxyacyl-CoA dehydratase [EC:4.2.1.134] | 12.34 | 12.95 | 10.41 | 5.24 | 4.37 | 4.31 | 1.83 | 0.49 | 0.81 |  |
| CL6953.Contig3_All | K10703 very-long-chain (3R)-3-hydroxyacyl-CoA dehydratase [EC:4.2.1.134] | 0.86 | 0.77 | 1.05 | 1.64 | 0.51 | 0.39 | 3.52 | 34.79 | 18.61 |  |
| Unigene33616_All | K10703 very-long-chain (3R)-3-hydroxyacyl-CoA dehydratase [EC:4.2.1.134] | 3.02 | 4.63 | 7.18 | 5.23 | 4.36 | 2.54 | 2.36 | 0.75 | 0.85 |  |
| CL15006.Contig1_All | K15400 omega-hydroxypalmitate O-feruloyl transferase [EC:2.3.1.188] | 33.52 | 36.32 | 31.28 | 12.55 | 6.67 | 5.42 | 4.61 | 5.7 | 1.13 |  |
| CL15006.Contig2_All | K15400 omega-hydroxypalmitate O-feruloyl transferase [EC:2.3.1.188] | 34.4 | 38.51 | 32.08 | 12.27 | 6.66 | 5 | 5.23 | 5.14 | 0.82 |  |
| CL2680.Contig6_All | K15400 omega-hydroxypalmitate O-feruloyl transferase [EC:2.3.1.188] | 2.32 | 2.54 | 2.79 | 2.97 | 1.87 | 1.8 | 0.62 | 0.45 | 0.66 |  |
| Unigene12950_All | K15400 omega-hydroxypalmitate O-feruloyl transferase [EC:2.3.1.188] | 0 | 0 | 0 | 0.53 | 0.22 | 0.3 | 0 | 0 | 0 |  |
| Unigene37567_All | K15400 omega-hydroxypalmitate O-feruloyl transferase [EC:2.3.1.188] | 0.19 | 0.69 | 0.22 | 8.21 | 0.97 | 9.91 | 0 | 0 | 0 |  |
| Unigene42058_All | K15400 omega-hydroxypalmitate O-feruloyl transferase [EC:2.3.1.188] | 4.29 | 5.02 | 5.41 | 0.06 | 0.06 | 0.16 | 0 | 0 | 0.04 |  |
| CL16109.Contig1_All | K08241 jasmonate O-methyltransferase [EC:2.1.1.141] | 7.26 | 3.42 | 4.01 | 19.29 | 16.43 | 14.09 | 2.22 | 2.67 | 2.81 |  |
| CL16109.Contig2_All | K08241 jasmonate O-methyltransferase [EC:2.1.1.141] | 0.54 | 1.49 | 0.59 | 2.37 | 1.27 | 2.02 | 0 | 0 | 0.19 |  |
| CL16109.Contig3_All | K08241 jasmonate O-methyltransferase [EC:2.1.1.141] | 0.59 | 0.14 | 0.44 | 52.69 | 72.62 | 7.99 | 193.04 | 180.01 | 174.86 |  |
| CL16109.Contig4_All | K08241 jasmonate O-methyltransferase [EC:2.1.1.141] | 35.85 | 43.49 | 35 | 11.26 | 12.87 | 12.07 | 0 | 0 | 0 |  |
| CL16109.Contig5_All | K08241 jasmonate O-methyltransferase [EC:2.1.1.141] | 25.4 | 29.63 | 23.68 | 7.79 | 8.4 | 7.74 | 0 | 0 | 0 |  |
| CL8527.Contig1_All | K08241 jasmonate O-methyltransferase [EC:2.1.1.141] | 1.92 | 3.26 | 0.5 | 6.32 | 4.03 | 1.9 | 0 | 0 | 0 |  |
| CL8527.Contig2_All | K08241 jasmonate O-methyltransferase [EC:2.1.1.141] | 0.77 | 0.55 | 0 | 1.68 | 1 | 0.49 | 0.11 | 0 | 0.11 |  |
| CL2376.Contig1_All | K00059 3-oxoacyl-[acyl-carrier protein] reductase [EC:1.1.1.100] | 3.71 | 0.74 | 7.08 | 154.37 | 214.87 | 73.01 | 1.39 | 497.19 | 1.94 |  |
| CL2376.Contig2_All | K00059 3-oxoacyl-[acyl-carrier protein] reductase [EC:1.1.1.100] | 1.1 | 4.39 | 0.59 | 99.65 | 85.28 | 152.96 | 1415.45 | 448.97 | 1348.91 |  |
| CL2376.Contig3_All | K00059 3-oxoacyl-[acyl-carrier protein] reductase [EC:1.1.1.100] | 0.21 | 3.54 | 0.39 | 1.44 | 427.73 | 2.94 | 0.5 | 0 | 0 |  |
| CL2573.Contig1_All | K00059 3-oxoacyl-[acyl-carrier protein] reductase [EC:1.1.1.100] | 2.56 | 1.87 | 3.05 | 1.36 | 1.3 | 1.03 | 1.48 | 0.72 | 0.7 |  |
| CL7002.Contig5_All | K00059 3-oxoacyl-[acyl-carrier protein] reductase [EC:1.1.1.100] | 0.22 | 0.39 | 0.76 | 0.43 | 0 | 0.72 | 3.54 | 8.55 | 5.22 |  |
| CL7002.Contig6_All | K00059 3-oxoacyl-[acyl-carrier protein] reductase [EC:1.1.1.100] | 0.67 | 0.93 | 0.71 | 0.54 | 0.54 | 0.56 | 3.88 | 3.32 | 4.05 |  |
| CL7002.Contig7_All | K00059 3-oxoacyl-[acyl-carrier protein] reductase [EC:1.1.1.100] | 0 | 0.14 | 0 | 0 | 0 | 0.06 | 0.85 | 0.65 | 1.05 |  |
| Unigene20183_All | K00059 3-oxoacyl-[acyl-carrier protein] reductase [EC:1.1.1.100] | 165.19 | 191.42 | 164.23 | 76.07 | 71.11 | 94.84 | 45.67 | 38.84 | 53.71 |  |
| CL14889.Contig1_All | K09458 3-oxoacyl-[acyl-carrier-protein] synthase II [EC:2.3.1.179] | 55.83 | 64.22 | 68.82 | 32.51 | 24.31 | 27.3 | 3.78 | 3.54 | 4.6 |  |
| Unigene16003_All | K09458 3-oxoacyl-[acyl-carrier-protein] synthase II [EC:2.3.1.179] | 1.56 | 2.2 | 0.95 | 0.59 | 0.44 | 0.59 | 0.83 | 0.36 | 0.68 |  |
| Unigene29386_All | K09458 3-oxoacyl-[acyl-carrier-protein] synthase II [EC:2.3.1.179] | 55.01 | 61.69 | 57.55 | 19.04 | 15.08 | 19.08 | 6.42 | 5.96 | 9.7 |  |
| Unigene34228_All | K09458 3-oxoacyl-[acyl-carrier-protein] synthase II [EC:2.3.1.179] | 8.45 | 4.06 | 3.6 | 0.06 | 0.06 | 0.99 | 0 | 0.07 | 0 |  |
| Unigene37345_All | K00648 3-oxoacyl-[acyl-carrier-protein] synthase III [EC:2.3.1.180] | 44.67 | 50.51 | 42.09 | 16.44 | 17.95 | 17.65 | 16.2 | 11.73 | 14.23 |  |
| CL15103.Contig1_All | K10251 17beta-estradiol 17-dehydrogenase / very-long-chain 3-oxoacyl-CoA reductase [EC:1.1.1.62 1.1.1.330] | 471.25 | 311.95 | 393.5 | 822.18 | 923.26 | 630.48 | 17.08 | 15.9 | 18.4 |  |
| CL15103.Contig2_All | K10251 17beta-estradiol 17-dehydrogenase / very-long-chain 3-oxoacyl-CoA reductase [EC:1.1.1.62 1.1.1.330] | 1.58 | 2.87 | 2.07 | 3.07 | 3.35 | 1.8 | 0.06 | 0.11 | 0.24 |  |
| Unigene17156_All | K10251 17beta-estradiol 17-dehydrogenase / very-long-chain 3-oxoacyl-CoA reductase [EC:1.1.1.62 1.1.1.330] | 0.77 | 0.87 | 1.01 | 0.46 | 0.11 | 0.12 | 0 | 0.06 | 0 |  |
| Unigene7734_All | K10251 17beta-estradiol 17-dehydrogenase / very-long-chain 3-oxoacyl-CoA reductase [EC:1.1.1.62 1.1.1.330] | 2.02 | 2.51 | 2.15 | 1.42 | 0.79 | 0.69 | 0.29 | 0.16 | 0.29 |  |
| CL14039.Contig2_All | K15397 3-ketoacyl-CoA synthase [EC:2.3.1.199] | 56.09 | 56.93 | 51.89 | 29.88 | 23 | 22.9 | 2.07 | 0.41 | 0.39 |  |
| CL14705.Contig1_All | K15397 3-ketoacyl-CoA synthase [EC:2.3.1.199] | 6.8 | 2.86 | 2.01 | 0.23 | 0.06 | 0.09 | 0.06 | 0.03 | 0.29 |  |
| CL14705.Contig2_All | K15397 3-ketoacyl-CoA synthase [EC:2.3.1.199] | 13.41 | 13.12 | 9.14 | 0.13 | 0.05 | 0.11 | 0.21 | 0.03 | 0.56 |  |
| CL8909.Contig2_All | K15397 3-ketoacyl-CoA synthase [EC:2.3.1.199] | 4.97 | 3.7 | 3.53 | 1.17 | 1.37 | 1.08 | 2.02 | 1.48 | 1.82 |  |
| CL8909.Contig3_All | K15397 3-ketoacyl-CoA synthase [EC:2.3.1.199] | 5.3 | 3.26 | 4.25 | 1.36 | 1.38 | 1.01 | 2.05 | 1.45 | 1.41 |  |
| Unigene1248_All | K15397 3-ketoacyl-CoA synthase [EC:2.3.1.199] | 0.75 | 0.58 | 0.7 | 0.24 | 0.05 | 0.05 | 0 | 0 | 0 |  |
| Unigene20808_All | K15397 3-ketoacyl-CoA synthase [EC:2.3.1.199] | 0.93 | 1.08 | 0.73 | 0.07 | 0 | 0.07 | 0 | 0 | 0 |  |
| Unigene30412_All | K15397 3-ketoacyl-CoA synthase [EC:2.3.1.199] | 6.65 | 5.09 | 9.25 | 5.43 | 3.2 | 3.79 | 1.37 | 2.6 | 2.49 |  |
| Unigene30555_All | K15397 3-ketoacyl-CoA synthase [EC:2.3.1.199] | 1.43 | 1.14 | 1.12 | 1.13 | 1.48 | 1.13 | 0 | 0.03 | 0 |  |
| Unigene33652_All | K15397 3-ketoacyl-CoA synthase [EC:2.3.1.199] | 2.62 | 2.97 | 2.06 | 5.04 | 2.56 | 3.99 | 0.19 | 0.65 | 0.88 |  |
| Unigene37896_All | K15397 3-ketoacyl-CoA synthase [EC:2.3.1.199] | 0.52 | 0.41 | 0.57 | 0 | 0 | 0.04 | 0 | 0.09 | 0.05 |  |
| Unigene40102_All | K15397 3-ketoacyl-CoA synthase [EC:2.3.1.199] | 2.01 | 1.3 | 1.3 | 0.63 | 0.88 | 0.99 | 0.56 | 0.71 | 0.58 |  |
| Unigene82243_All | K15397 3-ketoacyl-CoA synthase [EC:2.3.1.199] | 29.62 | 27.55 | 25.31 | 18.32 | 16.48 | 13.29 | 13.52 | 8.87 | 18 |  |
| Unigene1206_All | K04708 3-dehydrosphinganine reductase [EC:1.1.1.102] | 2.02 | 2.43 | 1.94 | 0.25 | 0.59 | 0.44 | 0.49 | 0 | 0 |  |
| Unigene40220_All | K04708 3-dehydrosphinganine reductase [EC:1.1.1.102] | 0.72 | 1.77 | 0.82 | 1.91 | 1.16 | 1.97 | 4.05 | 3.19 | 3.36 |  |
| CL8536.Contig1_All | K01897 long-chain acyl-CoA synthetase [EC:6.2.1.3] | 0.97 | 1.17 | 0.93 | 1.19 | 0.69 | 1.15 | 2.47 | 1.86 | 2.93 |  |
| Unigene13215_All | K01897 long-chain acyl-CoA synthetase [EC:6.2.1.3] | 0.13 | 0 | 0.16 | 0 | 0.07 | 0.05 | 0.83 | 0.29 | 0.74 |  |
| Unigene19998_All | K01897 long-chain acyl-CoA synthetase [EC:6.2.1.3] | 29.19 | 35.53 | 41.23 | 20.26 | 16.8 | 16.37 | 17.17 | 13.85 | 12.86 |  |
| CL6438.Contig2_All | K01052 lysosomal acid lipase/cholesteryl ester hydrolase [EC:3.1.1.13] | 0 | 0 | 0 | 0 | 0 | 0 | 3.01 | 0.56 | 0 |  |
| Unigene20130_All | K01052 lysosomal acid lipase/cholesteryl ester hydrolase [EC:3.1.1.13] | 5.68 | 6.55 | 5.96 | 11.77 | 11.46 | 11.55 | 16.57 | 13.35 | 14.12 |  |
| Unigene33225_All | K01052 lysosomal acid lipase/cholesteryl ester hydrolase [EC:3.1.1.13] | 0.29 | 0.21 | 0.25 | 0.36 | 1.42 | 4.27 | 0.85 | 1.27 | 0.32 |  |
| CL8239.Contig1_All | K15718 linoleate 9S-lipoxygenase [EC:1.13.11.58] | 26.81 | 25.13 | 20.6 | 15.42 | 20.98 | 19.9 | 0 | 0.05 | 0 |  |
| CL8239.Contig2_All | K15718 linoleate 9S-lipoxygenase [EC:1.13.11.58] | 1.63 | 1.05 | 1.73 | 0.79 | 0.78 | 0.53 | 0 | 0 | 0 |  |
| Unigene2066_All | K15718 linoleate 9S-lipoxygenase [EC:1.13.11.58] | 0 | 0 | 0 | 0.18 | 0.7 | 0.41 | 0 | 0 | 0 |  |
| Unigene23569_All | K15718 linoleate 9S-lipoxygenase [EC:1.13.11.58] | 3.14 | 3.3 | 2.26 | 2.19 | 2.03 | 2.12 | 0.02 | 0.06 | 0.02 |  |
| Unigene26799_All | K15718 linoleate 9S-lipoxygenase [EC:1.13.11.58] | 23.19 | 31.28 | 20.47 | 9.99 | 10.77 | 9.98 | 12.3 | 6.36 | 13.43 |  |
| Unigene34215_All | K15718 linoleate 9S-lipoxygenase [EC:1.13.11.58] | 47.18 | 47.32 | 40.68 | 31.03 | 36.46 | 31.18 | 0.23 | 0 | 0.07 |  |
| Unigene36874_All | K15718 linoleate 9S-lipoxygenase [EC:1.13.11.58] | 0.12 | 0 | 0.15 | 0.05 | 0.1 | 0.15 | 0.62 | 0.9 | 0.6 |  |
| Unigene41337_All | K15718 linoleate 9S-lipoxygenase [EC:1.13.11.58] | 1.01 | 1.07 | 1.01 | 0.24 | 0.24 | 0.24 | 0 | 0 | 0 |  |
| CL14106.Contig1_All | K00454 lipoxygenase [EC:1.13.11.12] | 0.99 | 1.04 | 1.16 | 5.1 | 3.24 | 3.11 | 2.01 | 2 | 2.02 |  |
| CL14106.Contig3_All | K00454 lipoxygenase [EC:1.13.11.12] | 7.12 | 7.67 | 6.43 | 2.14 | 1.49 | 2 | 1.75 | 2.03 | 1.87 |  |
| CL16387.Contig1_All | K00454 lipoxygenase [EC:1.13.11.12] | 8.25 | 8.18 | 9.83 | 3.45 | 12.38 | 4.04 | 6.17 | 2.06 | 1.81 |  |
| CL5662.Contig1_All | K00454 lipoxygenase [EC:1.13.11.12] | 6.59 | 7.49 | 7.82 | 5.73 | 5.52 | 4.32 | 0.59 | 1.87 | 0.57 |  |
| CL5662.Contig2_All | K00454 lipoxygenase [EC:1.13.11.12] | 18.75 | 18.14 | 13.49 | 7.76 | 7.08 | 6.58 | 4.36 | 1.3 | 5.92 |  |
| CL5662.Contig4_All | K00454 lipoxygenase [EC:1.13.11.12] | 0.74 | 1.87 | 0.82 | 0.99 | 0.79 | 1.2 | 0.23 | 0.27 | 0 |  |
| Unigene15299_All | K00454 lipoxygenase [EC:1.13.11.12] | 0.6 | 0.67 | 0.73 | 1.73 | 1.15 | 1.32 | 1.1 | 0.72 | 0.87 |  |
| Unigene18961_All | K00454 lipoxygenase [EC:1.13.11.12] | 8.7 | 331.22 | 57.16 | 129.33 | 79.8 | 522.61 | 51.86 | 45.64 | 35.24 |  |
| Unigene205_All | K00454 lipoxygenase [EC:1.13.11.12] | 0.84 | 0.71 | 0.68 | 0.88 | 0.9 | 0.5 | 0.04 | 0.1 | 0 |  |
| Unigene44232_All | K00454 lipoxygenase [EC:1.13.11.12] | 4.55 | 7.82 | 2.56 | 4.06 | 3.83 | 26.97 | 0.84 | 0.16 | 1.2 |  |
| Unigene5976_All | K00454 lipoxygenase [EC:1.13.11.12] | 2.38 | 351.07 | 27.64 | 142.39 | 58.3 | 583.47 | 24.1 | 49.81 | 39.62 |  |
| Unigene7769_All | K00454 lipoxygenase [EC:1.13.11.12] | 1.9 | 23.03 | 4.73 | 10.01 | 7.22 | 41.85 | 3.85 | 3.48 | 2.42 |  |
| CL10192.Contig1_All | K00655 1-acyl-sn-glycerol-3-phosphate acyltransferase [EC:2.3.1.51] | 8.26 | 8.53 | 4.09 | 2.94 | 5.49 | 1.66 | 2.76 | 2.35 | 2.14 |  |
| CL3672.Contig1_All | K00655 1-acyl-sn-glycerol-3-phosphate acyltransferase [EC:2.3.1.51] | 14.1 | 12.76 | 13.01 | 21.44 | 11.38 | 11.38 | 51.15 | 38.36 | 36.36 |  |
| CL3672.Contig6_All | K00655 1-acyl-sn-glycerol-3-phosphate acyltransferase [EC:2.3.1.51] | 13.72 | 13.64 | 11.43 | 19.49 | 8 | 12.2 | 44.12 | 30.04 | 31.08 |  |
| Unigene40734_All | K00655 1-acyl-sn-glycerol-3-phosphate acyltransferase [EC:2.3.1.51] | 8.05 | 7.2 | 6.84 | 4.48 | 4.38 | 5.17 | 3.87 | 2.83 | 4 |  |
| CL6245.Contig1_All | K06130 lysophospholipase II [EC:3.1.1.5] | 7.08 | 12.97 | 10.75 | 4.76 | 5.29 | 3.04 | 2.83 | 2.48 | 2.17 |  |
| CL6245.Contig2_All | K06130 lysophospholipase II [EC:3.1.1.5] | 8.55 | 11.12 | 8.1 | 5.27 | 4.08 | 3.92 | 3.46 | 2.92 | 2.46 |  |
| Unigene20258_All | K06130 lysophospholipase II [EC:3.1.1.5] | 14.42 | 22.76 | 20.39 | 13.94 | 10.39 | 9.03 | 2.12 | 2.08 | 2.08 |  |
| Unigene30452_All | K06129 lysophospholipase III [EC:3.1.1.5] | 5.43 | 5.51 | 5.36 | 6.67 | 5.94 | 6.28 | 1.14 | 2.3 | 2.44 |  |
| Unigene20594_All | K15405 midchain alkane hydroxylase | 0.03 | 0 | 0 | 1.76 | 0.69 | 1.1 | 0.36 | 4.18 | 4.26 |  |
| Unigene20202_All | K00645 [acyl-carrier-protein] S-malonyltransferase [EC:2.3.1.39] | 21.65 | 20.62 | 19.04 | 8.13 | 4.15 | 7.75 | 3.79 | 5.06 | 4.94 |  |
| CL10351.Contig1_All | K08242 24-methylenesterol C-methyltransferase [EC:2.1.1.143] | 100.73 | 90.9 | 86.83 | 83.41 | 68.87 | 62.54 | 40.69 | 40.63 | 29.71 |  |
| CL10351.Contig2_All | K08242 24-methylenesterol C-methyltransferase [EC:2.1.1.143] | 76.26 | 69.02 | 68.78 | 62.39 | 48.87 | 42.43 | 30.19 | 32.17 | 20.13 |  |
| Unigene24767_All | K07512 mitochondrial trans-2-enoyl-CoA reductase [EC:1.3.1.38] | 0.53 | 0.74 | 0.78 | 0.09 | 0.1 | 0.15 | 0.1 | 0.11 | 0.33 |  |
| CL3462.Contig3_All | K14457 2-acylglycerol O-acyltransferase 2 [EC:2.3.1.22] | 6.46 | 8.94 | 5.8 | 8.43 | 6.21 | 8.35 | 4.27 | 3.28 | 3.65 |  |
| CL5052.Contig4_All | K14457 2-acylglycerol O-acyltransferase 2 [EC:2.3.1.22] | 0.92 | 5.48 | 2.14 | 6.43 | 4.56 | 3.2 | 9.83 | 13.39 | 11.8 |  |
| CL4776.Contig1_All | K03715 1,2-diacylglycerol 3-beta-galactosyltransferase [EC:2.4.1.46] | 2.24 | 2.46 | 2.39 | 6.75 | 4.61 | 3.43 | 1.71 | 1.57 | 2.18 |  |
| CL4776.Contig4_All | K03715 1,2-diacylglycerol 3-beta-galactosyltransferase [EC:2.4.1.46] | 0.81 | 1.17 | 1.29 | 3.54 | 2.37 | 2.05 | 0.64 | 1.17 | 0.72 |  |
| CL12466.Contig1_All | K01054 acylglycerol lipase [EC:3.1.1.23] | 31.43 | 30.83 | 28 | 100.2 | 90.7 | 54.36 | 31.14 | 31.84 | 43.33 |  |
| CL13725.Contig1_All | K01054 acylglycerol lipase [EC:3.1.1.23] | 15.98 | 24.14 | 15.37 | 52.65 | 31.08 | 36.05 | 70.13 | 80.14 | 71.44 |  |
| CL13725.Contig2_All | K01054 acylglycerol lipase [EC:3.1.1.23] | 2.74 | 3.92 | 4.67 | 7.98 | 15.06 | 5.29 | 19.87 | 2.83 | 10.79 |  |
| CL14491.Contig1_All | K01054 acylglycerol lipase [EC:3.1.1.23] | 12.28 | 18.27 | 11.84 | 5.2 | 2.83 | 4.24 | 1.02 | 3.84 | 0 |  |
| CL14491.Contig2_All | K01054 acylglycerol lipase [EC:3.1.1.23] | 28.11 | 20.15 | 22.56 | 6.74 | 5.43 | 5.6 | 1.72 | 2.31 | 4.42 |  |
| CL4703.Contig1_All | K01054 acylglycerol lipase [EC:3.1.1.23] | 0.69 | 1.15 | 0 | 0.44 | 0.08 | 0.46 | 0 | 0 | 0 |  |
| CL4703.Contig4_All | K01054 acylglycerol lipase [EC:3.1.1.23] | 0.53 | 0.35 | 0.43 | 0.21 | 0.18 | 0 | 0.04 | 0 | 0.04 |  |
| CL4703.Contig5_All | K01054 acylglycerol lipase [EC:3.1.1.23] | 0.87 | 0.72 | 0.87 | 0.21 | 0.62 | 0.47 | 0.19 | 0 | 0.11 |  |
| CL4703.Contig7_All | K01054 acylglycerol lipase [EC:3.1.1.23] | 0.89 | 1.32 | 1.87 | 0.63 | 0.64 | 0.35 | 0.21 | 0 | 0.08 |  |
| CL4703.Contig8_All | K01054 acylglycerol lipase [EC:3.1.1.23] | 2.83 | 3.26 | 2.39 | 0.97 | 0.81 | 1.12 | 0.29 | 0.19 | 0.31 |  |
| CL4943.Contig3_All | K01054 acylglycerol lipase [EC:3.1.1.23] | 12.79 | 12.44 | 10.9 | 59.64 | 38 | 45.45 | 20.25 | 18.76 | 21.88 |  |
| CL4943.Contig5_All | K01054 acylglycerol lipase [EC:3.1.1.23] | 9.53 | 12.25 | 9.54 | 45.99 | 37.97 | 43.52 | 12.85 | 16.28 | 21.36 |  |
| CL5476.Contig1_All | K01054 acylglycerol lipase [EC:3.1.1.23] | 7.57 | 3.67 | 4.76 | 45.77 | 36.4 | 21.91 | 0.15 | 0.11 | 0 |  |
| CL873.Contig3_All | K01054 acylglycerol lipase [EC:3.1.1.23] | 0.53 | 1.4 | 2.26 | 4.59 | 6.76 | 6.13 | 1.18 | 0.71 | 0.86 |  |
| Unigene12830_All | K01054 acylglycerol lipase [EC:3.1.1.23] | 0.64 | 0.22 | 0 | 0.16 | 0.3 | 0 | 3.05 | 3.6 | 5.1 |  |
| Unigene13181_All | K01054 acylglycerol lipase [EC:3.1.1.23] | 0.04 | 0.29 | 0.15 | 0.12 | 0.12 | 0.22 | 2.95 | 3.15 | 1.72 |  |
| Unigene13917_All | K01054 acylglycerol lipase [EC:3.1.1.23] | 0 | 0 | 0 | 0 | 0 | 0.13 | 3.29 | 1.84 | 0.26 |  |
| Unigene14040_All | K01054 acylglycerol lipase [EC:3.1.1.23] | 0 | 0.05 | 0 | 0 | 0 | 0.09 | 1.76 | 7.97 | 0.95 |  |
| Unigene14107_All | K01054 acylglycerol lipase [EC:3.1.1.23] | 0.09 | 0.02 | 0.11 | 0.05 | 0.09 | 0.12 | 2.13 | 2.13 | 0.78 |  |
| Unigene15608_All | K01054 acylglycerol lipase [EC:3.1.1.23] | 0 | 0 | 0 | 0 | 0 | 0 | 1.04 | 2.45 | 0.31 |  |
| Unigene29979_All | K01054 acylglycerol lipase [EC:3.1.1.23] | 4.45 | 2.7 | 2.48 | 3.6 | 3.59 | 1.88 | 1.2 | 1.02 | 1.91 |  |
| Unigene34190_All | K01054 acylglycerol lipase [EC:3.1.1.23] | 147.03 | 115.41 | 111.21 | 174.41 | 169.58 | 118.33 | 0.24 | 0.04 | 0.49 |  |
| Unigene40947_All | K01054 acylglycerol lipase [EC:3.1.1.23] | 1.87 | 2.1 | 2.89 | 0.45 | 0.06 | 0.39 | 0 | 0 | 0.08 |  |
| Unigene44281_All | K01054 acylglycerol lipase [EC:3.1.1.23] | 23.27 | 21.25 | 19.93 | 22.81 | 21.43 | 18 | 0.84 | 0 | 0.32 |  |
| Unigene44448_All | K01054 acylglycerol lipase [EC:3.1.1.23] | 93.44 | 81.19 | 67.15 | 57.22 | 41.31 | 37 | 0 | 0.04 | 0.04 |  |
| Unigene6153_All | K01054 acylglycerol lipase [EC:3.1.1.23] | 0 | 0 | 0 | 0 | 0 | 0 | 2.89 | 1.07 | 1.34 |  |
| Unigene7042_All | K01054 acylglycerol lipase [EC:3.1.1.23] | 0 | 0 | 0.37 | 0 | 0 | 0 | 7.87 | 5.86 | 4.08 |  |
| Unigene8932_All | K01054 acylglycerol lipase [EC:3.1.1.23] | 0 | 0 | 0 | 1.16 | 0 | 0.21 | 1.4 | 1.81 | 3 |  |
| CL10135.Contig2_All | K05929 phosphoethanolamine N-methyltransferase [EC:2.1.1.103] | 36.12 | 50.75 | 46.26 | 2.09 | 1.15 | 3.1 | 0.06 | 0.17 | 0.12 |  |
| CL5796.Contig4_All | K07748 sterol-4alpha-carboxylate 3-dehydrogenase (decarboxylating) [EC:1.1.1.170] | 2.16 | 0.9 | 0.72 | 0.75 | 0 | 0 | 0.23 | 0.47 | 0.4 |  |
| CL5796.Contig7_All | K07748 sterol-4alpha-carboxylate 3-dehydrogenase (decarboxylating) [EC:1.1.1.170] | 5.92 | 5.27 | 3.91 | 2.44 | 2.25 | 1.37 | 2.5 | 1.02 | 2.01 |  |
| CL5796.Contig9_All | K07748 sterol-4alpha-carboxylate 3-dehydrogenase (decarboxylating) [EC:1.1.1.170] | 5.27 | 7.69 | 4.85 | 1.87 | 3.15 | 2.1 | 2.34 | 1.93 | 2.61 |  |
| Unigene26649_All | Oleosin | 300.36 | 210.82 | 240.14 | 1165.29 | 1016.43 | 933.91 | 703.65 | 689.74 | 634.03 |  |
| Unigene23397_All | Oleosin | 168.52 | 153.08 | 156.48 | 764.75 | 467.33 | 444.29 | 649.4 | 418.1 | 571.96 |  |
| CL8973.Contig1_All | Oleosin | 159.74 | 156.4 | 23 | 4367.14 | 3.42 | 2420.57 | 2165.81 | 2298.65 | 7098.4 |  |
| CL8973.Contig3_All | Oleosin | 148.01 | 124.06 | 163.83 | 1998.35 | 1652.42 | 1626.78 | 2693.2 | 2980.54 | 3605.9 |  |
| CL8973.Contig4_All | Oleosin | 90.62 | 93.91 | 62.26 | 416.98 | 33.19 | 195.55 | 138.83 | 200.97 | 885.71 |  |
| CL8973.Contig2_All | Oleosin | 46.55 | 19.98 | 131.41 | 11.11 | 1638.03 | 512.91 | 1705.08 | 1955.5 | 43.55 |  |
| CL10203.Contig2_All | K10526 OPC-8:0 CoA ligase 1 [EC:6.2.1.-] | 19.85 | 23.3 | 18.61 | 13.39 | 13.73 | 13.67 | 5.72 | 4.03 | 6.31 |  |
| CL10203.Contig3_All | K10526 OPC-8:0 CoA ligase 1 [EC:6.2.1.-] | 4.17 | 4.55 | 4.37 | 1.97 | 1.46 | 1.8 | 0 | 0 | 0 |  |
| CL755.Contig1_All | K10526 OPC-8:0 CoA ligase 1 [EC:6.2.1.-] | 21.09 | 21.62 | 19.15 | 14.47 | 13.53 | 12.69 | 5.05 | 4.85 | 3.3 |  |
| CL2517.Contig10_All | K05894 12-oxophytodienoic acid reductase [EC:1.3.1.42] | 6.26 | 7 | 4.16 | 3.53 | 2.3 | 2.62 | 0 | 0.21 | 0.49 |  |
| CL2517.Contig12_All | K05894 12-oxophytodienoic acid reductase [EC:1.3.1.42] | 6.66 | 8.77 | 3.99 | 2.35 | 3.46 | 2.79 | 0.76 | 0.35 | 0.4 |  |
| CL2517.Contig2_All | K05894 12-oxophytodienoic acid reductase [EC:1.3.1.42] | 7.52 | 7.92 | 7.58 | 18.92 | 15.19 | 10.46 | 4.74 | 3.27 | 3.08 |  |
| CL2517.Contig5_All | K05894 12-oxophytodienoic acid reductase [EC:1.3.1.42] | 0 | 0 | 1.9 | 3.42 | 1.38 | 0.91 | 0 | 0 | 0 |  |
| CL2517.Contig9_All | K05894 12-oxophytodienoic acid reductase [EC:1.3.1.42] | 4.23 | 6.55 | 4.11 | 5.71 | 5.4 | 4.2 | 1.05 | 2.21 | 1.91 |  |
| CL7124.Contig1_All | K05894 12-oxophytodienoic acid reductase [EC:1.3.1.42] | 36.67 | 36.44 | 34.98 | 23.56 | 17.93 | 16.61 | 9.5 | 4.97 | 7.95 |  |
| CL13997.Contig1_All | K15728 phosphatidate phosphatase LPIN [EC:3.1.3.4] | 1.92 | 2.2 | 1.75 | 3.93 | 2.93 | 3.99 | 4.41 | 10.55 | 11.7 |  |
| CL13997.Contig3_All | K15728 phosphatidate phosphatase LPIN [EC:3.1.3.4] | 5.19 | 6.36 | 4.85 | 6.24 | 7.26 | 6.35 | 13.44 | 12.01 | 14.34 |  |
| CL3787.Contig1_All | K15728 phosphatidate phosphatase LPIN [EC:3.1.3.4] | 3.96 | 4.75 | 3.8 | 7.56 | 6.71 | 7.62 | 15.92 | 24.74 | 18.49 |  |
| CL3787.Contig2_All | K15728 phosphatidate phosphatase LPIN [EC:3.1.3.4] | 3.38 | 4.45 | 3.24 | 6.55 | 6.21 | 6.48 | 21.11 | 22.74 | 21.78 |  |
| CL3787.Contig3_All | K15728 phosphatidate phosphatase LPIN [EC:3.1.3.4] | 0 | 0.41 | 0 | 0 | 0 | 0 | 7.19 | 0.04 | 7.85 |  |
| CL1150.Contig3_All | K00967 ethanolamine-phosphate cytidylyltransferase [EC:2.7.7.14] | 0.84 | 1.92 | 2.55 | 10.16 | 12.66 | 14.52 | 39.59 | 46.32 | 55.97 |  |
| CL15256.Contig2_All | K00967 ethanolamine-phosphate cytidylyltransferase [EC:2.7.7.14] | 2.51 | 3.53 | 3.26 | 5.36 | 4.81 | 1.26 | 39.21 | 10.47 | 9.51 |  |
| CL15256.Contig3_All | K00967 ethanolamine-phosphate cytidylyltransferase [EC:2.7.7.14] | 11.55 | 10.59 | 15.42 | 1.49 | 4.43 | 2.9 | 0.44 | 0.06 | 2.94 |  |
| Unigene36437_All | K00967 ethanolamine-phosphate cytidylyltransferase [EC:2.7.7.14] | 12.61 | 14.97 | 12.05 | 4.64 | 5.14 | 3.61 | 1.91 | 2.41 | 2.57 |  |
| CL14912.Contig1_All | K00679 phospholipid:diacylglycerol acyltransferase [EC:2.3.1.158] | 0.28 | 0.15 | 0.11 | 0.38 | 1.26 | 1.07 | 0.21 | 0.37 | 0.31 |  |
| Unigene43934_All | K00679 phospholipid:diacylglycerol acyltransferase [EC:2.3.1.158] | 11.51 | 12.45 | 11.48 | 11.33 | 10.2 | 10.83 | 6.24 | 4.52 | 4.63 |  |
| CL13909.Contig1_All | Phosphatidylcholine:diacylglycerol cholinephosphotransferase 1 | 39.42 | 29.39 | 42.17 | 11.1 | 13.83 | 14.21 | 1.01 | 1.29 | 1.81 |  |
| Unigene33843_All | K00995 CDP-diacylglycerol--glycerol-3-phosphate 3-phosphatidyltransferase [EC:2.7.8.5] | 10.22 | 11.63 | 9.87 | 5.87 | 6.24 | 4.86 | 4.06 | 3.94 | 5.84 |  |
| Unigene27449_All | K14674 TAG lipase / steryl ester hydrolase / phospholipase A2 / LPA acyltransferase [EC:3.1.1.3 3.1.1.13 3.1.1.4 2.3.1.51] | 4.67 | 9.71 | 3.31 | 4.26 | 3.47 | 8.04 | 0.09 | 0 | 0.18 |  |
| Unigene44246_All | K14674 TAG lipase / steryl ester hydrolase / phospholipase A2 / LPA acyltransferase [EC:3.1.1.3 3.1.1.13 3.1.1.4 2.3.1.51] | 0.55 | 2.06 | 0.95 | 1.36 | 0.6 | 0.84 | 0.26 | 0.17 | 0.18 |  |
| CL12271.Contig2_All | K14674 TAG lipase / steryl ester hydrolase / phospholipase A2 / LPA acyltransferase [EC:3.1.1.3 3.1.1.13 3.1.1.4 2.3.1.51] | 0.98 | 2.03 | 1.43 | 1.72 | 2.62 | 0.77 | 5.98 | 3.31 | 4.27 |  |
| CL4964.Contig1_All | K14674 TAG lipase / steryl ester hydrolase / phospholipase A2 / LPA acyltransferase [EC:3.1.1.3 3.1.1.13 3.1.1.4 2.3.1.51] | 7.12 | 11.67 | 6.16 | 8.18 | 5.92 | 8.69 | 4.31 | 16.34 | 32.74 |  |
| CL4964.Contig3_All | K14674 TAG lipase / steryl ester hydrolase / phospholipase A2 / LPA acyltransferase [EC:3.1.1.3 3.1.1.13 3.1.1.4 2.3.1.51] | 12.49 | 11.26 | 14.71 | 10.04 | 12.56 | 7.9 | 35.04 | 21 | 6.66 |  |
| CL12771.Contig1_All | K14674 TAG lipase / steryl ester hydrolase / phospholipase A2 / LPA acyltransferase [EC:3.1.1.3 3.1.1.13 3.1.1.4 2.3.1.51] | 11.88 | 10.99 | 11.69 | 7.35 | 6.92 | 8.71 | 20.09 | 21.44 | 17.55 |  |
| CL4542.Contig1_All | K14674 TAG lipase / steryl ester hydrolase / phospholipase A2 / LPA acyltransferase [EC:3.1.1.3 3.1.1.13 3.1.1.4 2.3.1.51] | 4.45 | 6.02 | 3.17 | 1.55 | 0.01 | 1.5 | 5.59 | 5.66 | 2.49 |  |
| CL10929.Contig1_All | K01114 phospholipase C [EC:3.1.4.3] | 84.84 | 76.64 | 74 | 70.18 | 50.69 | 57.24 | 5.27 | 10.96 | 8.52 |  |
| CL10929.Contig2_All | K01114 phospholipase C [EC:3.1.4.3] | 79 | 70.6 | 71.38 | 68.41 | 49.54 | 57.69 | 4.65 | 10.32 | 7.68 |  |
| CL14138.Contig1_All | K01114 phospholipase C [EC:3.1.4.3] | 13.35 | 18.35 | 14.49 | 4.08 | 4.03 | 5.55 | 0.08 | 0.14 | 0.09 |  |
| CL14138.Contig2_All | K01114 phospholipase C [EC:3.1.4.3] | 3.77 | 3.82 | 4.03 | 0.86 | 0.37 | 0.68 | 0.05 | 0 | 0 |  |
| Unigene20447_All | K01114 phospholipase C [EC:3.1.4.3] | 11.37 | 8.86 | 8.28 | 30.45 | 21.92 | 12.56 | 0.26 | 0 | 0.06 |  |
| CL10971.Contig2_All | K01115 phospholipase D1/2 [EC:3.1.4.4] | 2.33 | 2.84 | 3.4 | 1.02 | 0.79 | 0.56 | 0.35 | 0.53 | 0.53 |  |
| CL448.Contig1_All | K01115 phospholipase D1/2 [EC:3.1.4.4] | 195.97 | 162.11 | 180.97 | 99.59 | 108.05 | 89.31 | 71.3 | 65.78 | 76.61 |  |
| CL6834.Contig3_All | K01115 phospholipase D1/2 [EC:3.1.4.4] | 2.63 | 2.44 | 2.07 | 1.65 | 1.53 | 1.52 | 0.99 | 1.11 | 0.29 |  |
| CL939.Contig1_All | K01115 phospholipase D1/2 [EC:3.1.4.4] | 6.78 | 6.92 | 5.94 | 5.25 | 4.44 | 4.64 | 1.91 | 2.16 | 1.96 |  |
| CL939.Contig2_All | K01115 phospholipase D1/2 [EC:3.1.4.4] | 1.18 | 0.85 | 1.75 | 0.66 | 1.05 | 0.56 | 0.02 | 0.1 | 0.43 |  |
| CL939.Contig3_All | K01115 phospholipase D1/2 [EC:3.1.4.4] | 2.63 | 2.78 | 1.2 | 3.63 | 1.95 | 3.76 | 0 | 0.57 | 0.24 |  |
| Unigene11601_All | K01115 phospholipase D1/2 [EC:3.1.4.4] | 0.11 | 0.18 | 0.14 | 1 | 0.19 | 0.55 | 0 | 0 | 0 |  |
| Unigene12269_All | K01115 phospholipase D1/2 [EC:3.1.4.4] | 0.28 | 0.63 | 0.82 | 0.3 | 0.07 | 0.69 | 3.8 | 1.65 | 2.1 |  |
| Unigene24267_All | K01115 phospholipase D1/2 [EC:3.1.4.4] | 0.15 | 0 | 0 | 1.14 | 0.74 | 0.3 | 0 | 0.11 | 0.23 |  |
| Unigene36509_All | K01115 phospholipase D1/2 [EC:3.1.4.4] | 2.86 | 3.38 | 3.24 | 1.19 | 0.52 | 1.15 | 0.25 | 0.24 | 0 |  |
| CL3895.Contig1_All | K01074 palmitoyl-protein thioesterase [EC:3.1.2.22] | 0.57 | 1.26 | 0.19 | 0.27 | 0.7 | 0.78 | 4.26 | 1.8 | 5.27 |  |
| CL9109.Contig1_All | K01074 palmitoyl-protein thioesterase [EC:3.1.2.22] | 0.51 | 0.44 | 0.45 | 0.29 | 0.2 | 0.21 | 0.76 | 1.47 | 1.27 |  |
| Unigene23037_All | K01074 palmitoyl-protein thioesterase [EC:3.1.2.22] | 0.93 | 0.53 | 0.7 | 0.65 | 0.59 | 0.6 | 2.22 | 1.96 | 2.45 |  |
| Unigene23752_All | K01074 palmitoyl-protein thioesterase [EC:3.1.2.22] | 14.63 | 18.3 | 13.68 | 13.96 | 10.54 | 10.14 | 8.72 | 6.47 | 6.59 |  |
| Unigene33698_All | K15717 prostamide/prostaglandin F2alpha synthase [EC:1.11.1.20] | 13.95 | 11.35 | 14.42 | 6.66 | 5.26 | 5.42 | 5.54 | 7.73 | 6.32 |  |
| CL9914.Contig1_All | K01613 phosphatidylserine decarboxylase [EC:4.1.1.65] | 4.42 | 6.52 | 3.45 | 2.79 | 2.4 | 1.85 | 9.51 | 9.99 | 7.67 |  |
| CL4619.Contig7_All | K08730 phosphatidylserine synthase 2 [EC:2.7.8.29] | 0.05 | 0 | 0 | 0 | 0 | 0 | 0.2 | 0.51 | 0.22 |  |
| CL4619.Contig5_All | K08730 phosphatidylserine synthase 2 [EC:2.7.8.29] | 1.13 | 1.35 | 0.68 | 1.11 | 0.9 | 1.28 | 3.77 | 3.14 | 2.14 |  |
| CL10095.Contig1_All | K17991 peroxygenase [EC:1.11.2.3] | 19.2 | 25.12 | 17.35 | 0.18 | 0.36 | 0.73 | 0 | 0 | 0 |  |
| CL5378.Contig2_All | K17991 peroxygenase [EC:1.11.2.3] | 5.3 | 11.04 | 7.33 | 0.2 | 0 | 0 | 1.2 | 0.37 | 0.86 |  |
| CL5378.Contig3_All | K17991 peroxygenase [EC:1.11.2.3] | 3.08 | 4.45 | 4.06 | 0.07 | 0 | 0 | 1.51 | 0 | 0.23 |  |
| CL6439.Contig1_All | K17991 peroxygenase [EC:1.11.2.3] | 33.67 | 48.43 | 31.99 | 61.9 | 37.96 | 24.22 | 0.42 | 0.06 | 0.06 |  |
| CL6439.Contig2_All | K17991 peroxygenase [EC:1.11.2.3] | 22.3 | 31.41 | 22.95 | 54.39 | 33.57 | 23.01 | 0.3 | 0 | 0.51 |  |
| Unigene19369_All | K17991 peroxygenase [EC:1.11.2.3] | 0.4 | 1.54 | 1.25 | 3.04 | 1.31 | 1.16 | 0.06 | 0 | 0 |  |
| Unigene41867_All | K17991 peroxygenase [EC:1.11.2.3] | 0.49 | 0.72 | 0.39 | 0 | 0 | 0 | 0 | 0 | 0 |  |
| Unigene11242_All | K03921 acyl-[acyl-carrier-protein] desaturase [EC:1.14.19.2 1.14.19.11 1.14.19.26] | 0 | 0 | 0.13 | 0 | 0 | 0 | 0.7 | 0 | 0.84 |  |
| Unigene12815_All | K03921 acyl-[acyl-carrier-protein] desaturase [EC:1.14.19.2 1.14.19.11 1.14.19.26] | 0 | 0 | 0.36 | 0 | 0 | 0 | 1.16 | 0 | 0.48 |  |
| Unigene13977_All | K03921 acyl-[acyl-carrier-protein] desaturase [EC:1.14.19.2 1.14.19.11 1.14.19.26] | 187.93 | 217.95 | 247.36 | 111.03 | 128.22 | 106.49 | 19.82 | 10.72 | 14.4 |  |
| Unigene32879_All | K03921 acyl-[acyl-carrier-protein] desaturase [EC:1.14.19.2 1.14.19.11 1.14.19.26] | 169.89 | 113.15 | 108.53 | 63.26 | 128.81 | 66.97 | 5.48 | 16.71 | 39.87 |  |
| Unigene39711_All | K03921 acyl-[acyl-carrier-protein] desaturase [EC:1.14.19.2 1.14.19.11 1.14.19.26] | 47.89 | 27.46 | 31.99 | 14.32 | 40.39 | 18.23 | 5.06 | 1.46 | 8.34 |  |
| Unigene4234_All | K03921 acyl-[acyl-carrier-protein] desaturase [EC:1.14.19.2 1.14.19.11 1.14.19.26] | 43.27 | 64.17 | 40.27 | 40.49 | 29.2 | 35.18 | 5.35 | 8.06 | 6.57 |  |
| CL10085.Contig1_All | K01634 sphinganine-1-phosphate aldolase [EC:4.1.2.27] | 13.05 | 14.1 | 15.27 | 20.23 | 18.31 | 12.61 | 4.93 | 5.77 | 7.83 |  |
| CL10085.Contig2_All | K01634 sphinganine-1-phosphate aldolase [EC:4.1.2.27] | 10.49 | 11.6 | 10.46 | 12.05 | 14.96 | 13.43 | 3.42 | 3.18 | 4.35 |  |
| Unigene28208_All | K01634 sphinganine-1-phosphate aldolase [EC:4.1.2.27] | 6.86 | 8.26 | 9.69 | 11.83 | 10.66 | 7.47 | 2.82 | 4.74 | 5.11 |  |
| CL5840.Contig1_All | K14423 4,4-dimethyl-9beta,19-cyclopropylsterol-4alpha-methyl oxidase [EC:1.14.13.72] | 11.4 | 12.26 | 9.15 | 5.55 | 13.76 | 5.24 | 4.27 | 2.68 | 1.44 |  |
| Unigene16863_All | K14423 4,4-dimethyl-9beta,19-cyclopropylsterol-4alpha-methyl oxidase [EC:1.14.13.72] | 5.11 | 6.28 | 4.35 | 1.46 | 1.1 | 0.96 | 0 | 0.28 | 1 |  |
| CL4354.Contig1_All | K14424 4-alpha-methyl-delta7-sterol-4alpha-methyl oxidase [EC:1.14.13.72] | 10.43 | 13.07 | 11.32 | 4.66 | 4.33 | 3.83 | 1.23 | 1.14 | 0.91 |  |
| CL4354.Contig3_All | K14424 4-alpha-methyl-delta7-sterol-4alpha-methyl oxidase [EC:1.14.13.72] | 3.19 | 7.13 | 4.37 | 4.43 | 1.88 | 4.49 | 0.34 | 1.73 | 1.41 |  |
| CL10210.Contig2_All | K00511 squalene monooxygenase [EC:1.14.14.17] | 26.15 | 20.02 | 20.74 | 25.44 | 23.09 | 14.49 | 0.7 | 1.74 | 1.25 |  |
| Unigene29944_All | K00511 squalene monooxygenase [EC:1.14.14.17] | 7.6 | 9.33 | 7.69 | 2.5 | 2.07 | 3.03 | 0.77 | 0.45 | 0.88 |  |
| Unigene384_All | K00511 squalene monooxygenase [EC:1.14.14.17] | 6.67 | 7.6 | 5.54 | 6.47 | 6.07 | 6.58 | 0.78 | 0.37 | 0.46 |  |
| Unigene385_All | K00511 squalene monooxygenase [EC:1.14.14.17] | 7.23 | 9.56 | 5.96 | 7.86 | 7.27 | 6.85 | 1 | 0.54 | 0.34 |  |
| CL10423.Contig1_All | K04713 sphinganine C4-monooxygenase [EC:1.14.18.5] | 7.01 | 8.09 | 4.84 | 7.31 | 4.66 | 7.83 | 1.25 | 2.73 | 2.78 |  |
| CL10423.Contig2_All | K04713 sphinganine C4-monooxygenase [EC:1.14.18.5] | 23.84 | 22.05 | 23.26 | 8.39 | 9.53 | 8.39 | 2.45 | 1.47 | 1.85 |  |
| CL10867.Contig1_All | K04713 sphinganine C4-monooxygenase [EC:1.14.18.5] | 7.7 | 9.37 | 8.07 | 3.93 | 2.73 | 2.72 | 0.66 | 0.74 | 0.32 |  |
| CL10867.Contig2_All | K04713 sphinganine C4-monooxygenase [EC:1.14.18.5] | 7.88 | 7.43 | 4.84 | 2.81 | 3.94 | 1.48 | 1.32 | 0.64 | 0 |  |
| Unigene16750_All | K04713 sphinganine C4-monooxygenase [EC:1.14.18.5] | 30.76 | 34.39 | 28.16 | 37.03 | 29.29 | 32.32 | 94.67 | 131.18 | 135.66 |  |
| Unigene26956_All | K00222 Delta14-sterol reductase [EC:1.3.1.70] | 18.67 | 17.63 | 19.21 | 10.01 | 8.84 | 7.67 | 3.3 | 3.75 | 3.08 |  |
| CL12570.Contig1_All | K15406 wax-ester synthase / diacylglycerol O-acyltransferase [EC:2.3.1.75 2.3.1.20] | 0.5 | 1.39 | 0.78 | 0 | 0 | 0.04 | 0 | 0 | 0.26 |  |
| CL7151.Contig1_All | K15406 wax-ester synthase / diacylglycerol O-acyltransferase [EC:2.3.1.75 2.3.1.20] | 6.21 | 5.92 | 6.82 | 13.05 | 13.23 | 11.28 | 21.41 | 27.64 | 27.52 |  |
| Unigene37472_All | K15406 wax-ester synthase / diacylglycerol O-acyltransferase [EC:2.3.1.75 2.3.1.20] | 2.5 | 4 | 2.66 | 2.09 | 1.16 | 1.63 | 0.06 | 0.03 | 0.16 |  |
| CL5887.Contig1_All | K01962 acetyl-CoA carboxylase carboxyl transferase subunit alpha [EC:6.4.1.2] | 61.6 | 71.63 | 62.8 | 20.75 | 20 | 22.23 | 12.27 | 12.51 | 14.02 |  |
| Unigene30129_All | K01962 acetyl-CoA carboxylase carboxyl transferase subunit alpha [EC:6.4.1.2] | 3.11 | 0.73 | 2.22 | 0.02 | 0.43 | 0.17 | 0.08 | 0.02 | 0 |  |
| Unigene5237_All | K01962 acetyl-CoA carboxylase carboxyl transferase subunit alpha [EC:6.4.1.2] | 2.3 | 3.4 | 2.41 | 1.07 | 1.04 | 0.51 | 0 | 0.27 | 0 |  |
|  |  |  |  |  |  |  |  |  |  |  |  |
