# Supplementary material for: Identification of genes associated with the biosynthesis of unsaturated fatty acid and oil accumulation in herbaceous peony ‘Hangshao’ (Paeonia lactiflora ‘Hangshao’) seeds based on transcriptome analysis
Source: BMC Genomics. 2021 Feb 1;22:94. doi: 10.1186/s12864-020-07339-7 (PMC7849092; doi:10.1186/s12864-020-07339-7)
Supplement: Supplementary file 12 — Additional file 12: Table S9. One hundred eleven DEGs used for the cluster analysis [file 12864_2020_7339_MOESM12_ESM.docx]

| Table S9: 111 DEGs used for the cluster analysis | | | | | | | | | | | | | | | | |
| --- | --- | --- | --- | --- | --- | --- | --- | --- | --- | --- | --- | --- | --- | --- | --- | --- |
| **GeneID** | **Enzyme  abbreviation** | **Mean-30d FPKM** | **HS30d_1 FPKM** | **HS30d_2 FPKM** | **HS30d_3 FPKM** | **Mean-60d FPKM** | **HS60d_1 FPKM** | **HS60d_2 FPKM** | **HS60d_3 FPKM** | **Mean-90d FPKM** | **HS90d_1 FPKM** | **HS90d_2 FPKM** | **HS90d_3 FPKM** | **LOG(60/30,2)** | **LOG(90/60,2)** | **LOG(90/30,2)** |
| CL1727.Contig4_All | ACAA1 | 18.63 | 15.37 | 23.94 | 16.59 | 10.60 | 12.90 | 9.79 | 9.12 | 6.14 | 9.23 | 7.18 | 2.02 | -0.81 | -0.79 | -1.60 |
| Unigene41549_All | ACE | 1.90 | 1.40 | 3.03 | 1.27 | 0.09 | 0.05 | 0.14 | 0.09 | 0.01 | 0.01 | 0.01 | 0.01 | -4.35 | -3.22 | -7.57 |
| CL2966.Contig1_All | ACER3 | 1.11 | 0.86 | 0.74 | 1.74 | 1.62 | 1.98 | 1.38 | 1.51 | 7.92 | 8.50 | 7.15 | 8.11 | 0.54 | 2.29 | 2.83 |
| Unigene23060_All | ACOX1 | 35.80 | 34.54 | 36.90 | 35.96 | 62.69 | 69.27 | 56.92 | 61.87 | 140.78 | 177.55 | 106.50 | 138.29 | 0.81 | 1.17 | 1.98 |
| Unigene23270_All | adh | 14.35 | 13.60 | 17.21 | 12.23 | 9.70 | 10.78 | 9.61 | 8.71 | 3.52 | 4.99 | 2.73 | 2.85 | -0.56 | -1.46 | -2.03 |
| CL656.Contig5_All | ADH1 | 149.00 | 131.33 | 156.55 | 159.13 | 320.42 | 253.06 | 459.61 | 248.59 | 1036.75 | 1421.50 | 695.47 | 993.27 | 1.10 | 1.69 | 2.80 |
| CL4829.Contig5_All | ADH5 | 70.39 | 65.15 | 79.55 | 66.47 | 20.31 | 23.57 | 18.37 | 19.00 | 44.12 | 49.70 | 51.94 | 30.72 | -1.79 | 1.12 | -0.67 |
| CL15410.Contig1_All | ADPRM | 0.43 | 0.40 | 0.49 | 0.40 | 2.65 | 2.70 | 3.52 | 1.74 | 29.81 | 13.47 | 44.36 | 31.59 | 2.63 | 3.49 | 6.12 |
| CL5515.Contig7_All | AKR1B | 0.05 | 0.10 | 0.05 | 0.01 | 4.44 | 6.08 | 6.70 | 0.55 | 61.44 | 44.61 | 101.47 | 38.25 | 6.38 | 3.79 | 10.17 |
| CL476.Contig3_All | ALDH | 269.13 | 249.22 | 278.74 | 279.44 | 126.91 | 141.57 | 120.15 | 119.00 | 37.69 | 47.38 | 30.41 | 35.29 | -1.08 | -1.75 | -2.84 |
| CL7300.Contig2_All | ALDH7A1 | 0.73 | 0.42 | 1.00 | 0.78 | 0.31 | 0.46 | 0.17 | 0.29 | 5.23 | 3.00 | 5.35 | 7.33 | -1.26 | 4.09 | 2.83 |
| CL12584.Contig1_All | AOC | 29.82 | 33.22 | 30.78 | 25.47 | 17.40 | 19.38 | 17.49 | 15.33 | 8.24 | 7.73 | 7.71 | 9.27 | -0.78 | -1.08 | -1.86 |
| Unigene4688_All | AOS | 0.07 | 0.00 | 0.16 | 0.05 | 0.41 | 0.27 | 0.66 | 0.30 | 1.61 | 2.23 | 1.01 | 1.60 | 2.55 | 1.98 | 4.53 |
| CL3334.Contig9_All | ASAH2 | 38.04 | 33.36 | 44.28 | 36.47 | 13.24 | 15.72 | 12.25 | 11.75 | 6.46 | 7.42 | 5.68 | 6.28 | -1.52 | -1.04 | -2.56 |
| CL6932.Contig4_All | BC | 38.01 | 30.87 | 39.35 | 43.80 | 8.20 | 8.04 | 7.00 | 9.55 | 1.80 | 1.91 | 1.63 | 1.86 | -2.21 | -2.19 | -4.40 |
| Unigene23603_All | BCCP | 49.45 | 48.49 | 46.97 | 52.90 | 19.87 | 19.72 | 20.34 | 19.55 | 7.34 | 9.97 | 5.95 | 6.10 | -1.32 | -1.44 | -2.75 |
| Unigene5575_All | CAS1 | 11.86 | 8.26 | 10.92 | 16.39 | 3.18 | 0.63 | 8.34 | 0.56 | 0.88 | 2.55 | 0.05 | 0.03 | -1.90 | -1.86 | -3.76 |
| Unigene591_All | CDIPT | 15.49 | 12.83 | 18.29 | 15.34 | 8.10 | 9.61 | 8.11 | 6.59 | 5.74 | 5.50 | 6.77 | 4.95 | -0.93 | -0.50 | -1.43 |
| CL9810.Contig2_All | CDS1 | 3.33 | 2.84 | 2.86 | 4.29 | 2.98 | 3.12 | 2.06 | 3.76 | 9.28 | 9.28 | 9.87 | 8.68 | -0.16 | 1.64 | 1.48 |
| Unigene21334_All | CER1 | 0.53 | 0.39 | 0.87 | 0.33 | 0.04 | 0.01 | 0.00 | 0.11 | 0.01 | 0.01 | 0.01 | 0.01 | -3.73 | -2.00 | -5.73 |
| Unigene1069_All | CERS1_2_3_4 | 19.27 | 17.48 | 23.12 | 17.20 | 13.73 | 15.43 | 13.28 | 12.47 | 9.05 | 9.64 | 7.10 | 10.41 | -0.49 | -0.60 | -1.09 |
| CL2726.Contig2_All | CGT | 45.77 | 46.13 | 44.35 | 46.82 | 8.14 | 6.71 | 8.10 | 9.62 | 0.35 | 0.70 | 0.16 | 0.18 | -2.49 | -4.55 | -7.04 |
| CL1012.Contig1_All | CKI1 | 14.07 | 13.76 | 14.58 | 13.86 | 11.68 | 13.13 | 11.93 | 9.99 | 40.12 | 35.25 | 52.92 | 32.20 | -0.27 | 1.78 | 1.51 |
| CL6205.Contig10_All | CLO | 435.85 | 382.73 | 418.00 | 506.82 | 673.13 | 655.64 | 735.15 | 628.60 | 1272.66 | 1385.68 | 976.72 | 1455.59 | 0.63 | 0.92 | 1.55 |
| CL11313.Contig2_All | CYP2J | 0.03 | 0.01 | 0.01 | 0.08 | 6.55 | 12.92 | 6.28 | 0.44 | 1018.83 | 476.49 | 470.55 | 2109.46 | 7.62 | 7.28 | 14.90 |
| Unigene16781_All | CYP51 | 83.65 | 80.62 | 83.24 | 87.08 | 58.89 | 64.03 | 61.22 | 51.42 | 28.02 | 30.30 | 26.14 | 27.63 | -0.51 | -1.07 | -1.58 |
| Unigene16366_All | CYP86A4S | 13.04 | 8.26 | 19.42 | 11.44 | 0.34 | 0.58 | 0.04 | 0.40 | 0.16 | 0.22 | 0.02 | 0.25 | -5.26 | -1.06 | -6.32 |
| Unigene34331_All | CYP86B1 | 8.04 | 7.93 | 9.86 | 6.34 | 3.54 | 4.24 | 3.10 | 3.27 | 0.01 | 0.01 | 0.01 | 0.01 | -1.19 | -8.47 | -9.65 |
| CL8836.Contig1_All | DAD1 | 6.58 | 6.26 | 7.25 | 6.22 | 1.42 | 0.49 | 2.83 | 0.95 | 1.14 | 1.03 | 2.26 | 0.14 | -2.21 | -0.32 | -2.52 |
| CL6160.Contig2_All | DGAT | 3.37 | 2.73 | 4.95 | 2.44 | 4.41 | 4.14 | 5.10 | 3.99 | 8.88 | 7.68 | 10.06 | 8.90 | 0.39 | 1.01 | 1.40 |
| CL103.Contig1_All | DGD | 7.16 | 7.15 | 7.62 | 6.72 | 6.02 | 7.46 | 5.71 | 4.89 | 1.81 | 1.36 | 1.11 | 2.96 | -0.25 | -1.73 | -1.98 |
| Unigene742_All | DGK | 20.66 | 19.11 | 21.76 | 21.11 | 16.93 | 20.48 | 17.15 | 13.17 | 4.17 | 3.83 | 3.49 | 5.19 | -0.29 | -2.02 | -2.31 |
| CL8255.Contig2_All | DHCR24 | 60.39 | 62.68 | 59.42 | 59.07 | 25.13 | 28.25 | 23.35 | 23.80 | 19.53 | 19.92 | 22.02 | 16.65 | -1.26 | -0.36 | -1.63 |
| Unigene27021_All | DHCR7 | 29.19 | 28.33 | 31.99 | 27.24 | 16.43 | 17.29 | 16.41 | 15.58 | 10.93 | 12.44 | 7.26 | 13.09 | -0.83 | -0.59 | -1.42 |
| CL16386.Contig1_All | DMT1 | 46.90 | 43.68 | 50.70 | 46.32 | 19.68 | 22.40 | 18.22 | 18.42 | 4.81 | 4.92 | 4.07 | 5.44 | -1.25 | -2.03 | -3.29 |
| CL289.Contig2_All | DPPL | 0.17 | 0.15 | 0.01 | 0.34 | 0.85 | 1.30 | 0.16 | 1.09 | 5.80 | 1.70 | 6.33 | 9.36 | 2.35 | 2.77 | 5.12 |
| CL7296.Contig2_All | EAR | 53.95 | 30.18 | 68.60 | 63.06 | 6.21 | 4.83 | 3.35 | 10.46 | 0.07 | 0.15 | 0.04 | 0.01 | -3.12 | -6.54 | -9.66 |
| Unigene16292_All | EBP | 9.55 | 9.53 | 10.14 | 8.99 | 4.83 | 6.84 | 4.13 | 3.51 | 0.87 | 1.03 | 0.39 | 1.18 | -0.98 | -2.48 | -3.46 |
| CL3941.Contig3_All | ECR | 13.23 | 10.67 | 13.49 | 15.53 | 15.63 | 16.47 | 17.54 | 12.89 | 42.81 | 48.80 | 43.82 | 35.80 | 0.24 | 1.45 | 1.69 |
| Unigene44412_All | EPHX2 | 13.56 | 11.44 | 13.80 | 15.44 | 54.38 | 56.72 | 69.08 | 37.34 | 384.22 | 296.85 | 436.85 | 418.95 | 2.00 | 2.82 | 4.82 |
| CL12825.Contig1_All | EPT1 | 29.48 | 30.02 | 32.76 | 25.65 | 16.69 | 18.67 | 16.55 | 14.84 | 8.96 | 7.77 | 9.81 | 9.29 | -0.82 | -0.90 | -1.72 |
| CL2636.Contig4_All | FAD2 | 618.85 | 785.17 | 405.49 | 665.90 | 292.97 | 332.46 | 217.02 | 329.44 | 2.68 | 3.95 | 1.36 | 2.72 | -1.08 | -6.77 | -7.85 |
| CL2686.Contig6_All | FAD3 | 480.23 | 439.22 | 520.31 | 481.17 | 335.31 | 262.84 | 177.35 | 565.75 | 0.92 | 1.76 | 0.39 | 0.61 | -0.52 | -8.51 | -9.03 |
| CL13349.Contig1_All | FAD7 | 7.88 | 7.02 | 7.37 | 9.24 | 6.70 | 5.87 | 7.51 | 6.72 | 0.14 | 0.30 | 0.01 | 0.11 | -0.23 | -5.58 | -5.81 |
| Unigene34203_All | FAD8 | 41.43 | 50.53 | 30.32 | 43.45 | 31.54 | 22.37 | 38.39 | 33.85 | 0.01 | 0.01 | 0.01 | 0.01 | -0.39 | -11.62 | -12.02 |
| CL244.Contig2_All | FAR | 40.09 | 40.09 | 36.53 | 43.64 | 36.59 | 46.80 | 35.22 | 27.74 | 0.01 | 0.01 | 0.01 | 0.01 | -0.13 | -11.84 | -11.97 |
| CL14443.Contig1_All | FATA | 45.73 | 44.91 | 45.88 | 46.39 | 21.26 | 24.00 | 18.52 | 21.27 | 11.71 | 13.51 | 8.80 | 12.81 | -1.10 | -0.86 | -1.97 |
| Unigene26774_All | FATB | 75.44 | 69.36 | 83.51 | 73.45 | 42.12 | 40.97 | 40.73 | 44.65 | 32.45 | 31.01 | 38.44 | 27.91 | -0.84 | -0.38 | -1.22 |
| CL13056.Contig1_All | FDFT1 | 185.97 | 197.71 | 189.15 | 171.04 | 2.66 | 2.69 | 2.14 | 3.15 | 0.93 | 1.20 | 0.09 | 1.50 | -6.13 | -1.52 | -7.64 |
| CL8793.Contig2_All | galA | 36.54 | 36.20 | 40.16 | 33.26 | 122.20 | 153.89 | 133.91 | 78.80 | 9.56 | 7.66 | 10.66 | 10.35 | 1.74 | -3.68 | -1.93 |
| CL11012.Contig2_All | GBA2 | 7.49 | 7.28 | 7.76 | 7.44 | 4.51 | 5.29 | 3.94 | 4.30 | 1.01 | 0.67 | 1.45 | 0.92 | -0.73 | -2.15 | -2.89 |
| CL11992.Contig2_All | GDE1 | 2.67 | 5.01 | 0.20 | 2.80 | 30.30 | 25.69 | 43.35 | 21.87 | 29.52 | 22.86 | 16.47 | 49.24 | 3.50 | -0.04 | 3.47 |
| CL7829.Contig2_All | GEP4 | 3.05 | 3.16 | 3.44 | 2.54 | 0.68 | 0.92 | 0.56 | 0.57 | 2.31 | 1.82 | 2.12 | 2.98 | -2.16 | 1.76 | -0.40 |
| Unigene41023_All | GGT1_5 | 30.45 | 28.85 | 35.63 | 26.88 | 22.59 | 25.10 | 23.95 | 18.71 | 0.12 | 0.08 | 0.08 | 0.21 | -0.43 | -7.52 | -7.95 |
| CL14885.Contig1_All | GK | 10.88 | 12.31 | 10.35 | 9.99 | 17.66 | 19.88 | 19.77 | 13.33 | 7.66 | 7.58 | 6.15 | 9.24 | 0.70 | -1.21 | -0.51 |
| CL6711.Contig1_All | GLB1 | 19.25 | 20.52 | 20.12 | 17.10 | 40.76 | 43.74 | 38.25 | 40.30 | 111.09 | 103.79 | 115.95 | 113.54 | 1.08 | 1.45 | 2.53 |
| CL265.Contig4_All | glpQ | 109.83 | 115.26 | 113.33 | 100.90 | 52.55 | 50.96 | 55.70 | 50.99 | 3.92 | 4.59 | 4.66 | 2.51 | -1.06 | -3.74 | -4.81 |
| CL13606.Contig3_All | GLYK | 1.49 | 1.28 | 1.98 | 1.22 | 1.73 | 2.12 | 1.23 | 1.85 | 4.36 | 5.44 | 2.32 | 5.33 | 0.22 | 1.33 | 1.55 |
| Unigene30381_All | GPAT | 7.74 | 7.02 | 9.91 | 6.29 | 3.60 | 4.20 | 2.84 | 3.77 | 2.27 | 3.23 | 2.09 | 1.50 | -1.10 | -0.66 | -1.77 |
| CL12015.Contig1_All | GPD1 | 18.37 | 20.48 | 16.90 | 17.72 | 20.51 | 23.28 | 20.87 | 17.39 | 9.44 | 9.41 | 8.34 | 10.58 | 0.16 | -1.12 | -0.96 |
| CL6460.Contig2_All | gpx | 66.37 | 50.82 | 72.79 | 75.49 | 42.46 | 50.32 | 40.60 | 36.46 | 9.81 | 7.59 | 7.72 | 14.11 | -0.64 | -2.11 | -2.76 |
| CL1701.Contig5_All | HAD | 43.29 | 48.33 | 40.85 | 40.68 | 13.69 | 14.10 | 13.20 | 13.76 | 13.45 | 15.35 | 11.76 | 13.23 | -1.66 | -0.03 | -1.69 |
| CL6953.Contig3_All | HCD | 0.89 | 0.86 | 0.77 | 1.05 | 0.85 | 1.64 | 0.51 | 0.39 | 18.97 | 3.52 | 34.79 | 18.61 | -0.08 | 4.49 | 4.41 |
| CL15006.Contig2_All | HHT1 | 35.00 | 34.40 | 38.51 | 32.08 | 7.98 | 12.27 | 6.66 | 5.00 | 3.73 | 5.23 | 5.14 | 0.82 | -2.13 | -1.10 | -3.23 |
| CL16109.Contig3_All | JMT | 0.39 | 0.59 | 0.14 | 0.44 | 44.43 | 52.69 | 72.62 | 7.99 | 182.64 | 193.04 | 180.01 | 174.86 | 6.83 | 2.04 | 8.87 |
| CL2376.Contig2_All | KAR | 2.03 | 1.10 | 4.39 | 0.59 | 112.63 | 99.65 | 85.28 | 152.96 | 1071.11 | 1415.45 | 448.97 | 1348.91 | 5.80 | 3.25 | 9.05 |
| CL14889.Contig1_All | KASII | 62.96 | 55.83 | 64.22 | 68.82 | 28.04 | 32.51 | 24.31 | 27.30 | 3.97 | 3.78 | 3.54 | 4.60 | -1.17 | -2.82 | -3.99 |
| Unigene37345_All | KASIII | 45.76 | 44.67 | 50.51 | 42.09 | 17.35 | 16.44 | 17.95 | 17.65 | 14.05 | 16.20 | 11.73 | 14.23 | -1.40 | -0.30 | -1.70 |
| CL15103.Contig1_All | KCR | 392.23 | 471.25 | 311.95 | 393.50 | 791.97 | 822.18 | 923.26 | 630.48 | 17.13 | 17.08 | 15.90 | 18.40 | 1.01 | -5.53 | -4.52 |
| CL14039.Contig2_All | KCS | 54.97 | 56.09 | 56.93 | 51.89 | 25.26 | 29.88 | 23.00 | 22.90 | 0.96 | 2.07 | 0.41 | 0.39 | -1.12 | -4.72 | -5.84 |
| Unigene40220_All | KDSR | 1.10 | 0.72 | 1.77 | 0.82 | 1.68 | 1.91 | 1.16 | 1.97 | 3.53 | 4.05 | 3.19 | 3.36 | 0.61 | 1.07 | 1.68 |
| Unigene19998_All | LACS | 35.32 | 29.19 | 35.53 | 41.23 | 17.81 | 20.26 | 16.80 | 16.37 | 14.63 | 17.17 | 13.85 | 12.86 | -0.99 | -0.28 | -1.27 |
| Unigene20130_All | LIPA | 6.06 | 5.68 | 6.55 | 5.96 | 11.59 | 11.77 | 11.46 | 11.55 | 14.68 | 16.57 | 13.35 | 14.12 | 0.94 | 0.34 | 1.28 |
| Unigene34215_All | LOX1_5 | 45.06 | 47.18 | 47.32 | 40.68 | 32.89 | 31.03 | 36.46 | 31.18 | 0.10 | 0.23 | 0.00 | 0.07 | -0.45 | -8.36 | -8.82 |
| Unigene5976_All | LOX2S | 127.03 | 2.38 | 351.07 | 27.64 | 261.39 | 142.39 | 58.30 | 583.47 | 37.84 | 24.10 | 49.81 | 39.62 | 1.04 | -2.79 | -1.75 |
| CL3672.Contig1_All | LPAAT | 13.29 | 14.10 | 12.76 | 13.01 | 14.73 | 21.44 | 11.38 | 11.38 | 41.96 | 51.15 | 38.36 | 36.36 | 0.15 | 1.51 | 1.66 |
| Unigene20258_All | LYPLA2 | 19.19 | 14.42 | 22.76 | 20.39 | 11.12 | 13.94 | 10.39 | 9.03 | 2.09 | 2.12 | 2.08 | 2.08 | -0.79 | -2.41 | -3.20 |
| Unigene30452_All | LYPLA3 | 5.43 | 5.43 | 5.51 | 5.36 | 6.30 | 6.67 | 5.94 | 6.28 | 1.96 | 1.14 | 2.30 | 2.44 | 0.21 | -1.68 | -1.47 |
| Unigene20594_All | MAH1 | 0.01 | 0.03 | 0.00 | 0.00 | 1.18 | 1.76 | 0.69 | 1.10 | 2.93 | 0.36 | 4.18 | 4.26 | 6.89 | 1.31 | 8.20 |
| Unigene20202_All | MCAT | 20.44 | 21.65 | 20.62 | 19.04 | 6.68 | 8.13 | 4.15 | 7.75 | 4.60 | 3.79 | 5.06 | 4.94 | -1.61 | -0.54 | -2.15 |
| CL10351.Contig1_All | MCMT | 92.82 | 100.73 | 90.90 | 86.83 | 71.61 | 83.41 | 68.87 | 62.54 | 37.01 | 40.69 | 40.63 | 29.71 | -0.37 | -0.95 | -1.33 |
| CL5052.Contig4_All | MGAT2 | 2.85 | 0.92 | 5.48 | 2.14 | 4.73 | 6.43 | 4.56 | 3.20 | 11.67 | 9.83 | 13.39 | 11.80 | 0.73 | 1.30 | 2.04 |
| CL4776.Contig1_All | MGD | 2.36 | 2.24 | 2.46 | 2.39 | 4.93 | 6.75 | 4.61 | 3.43 | 1.82 | 1.71 | 1.57 | 2.18 | 1.06 | -1.44 | -0.38 |
| CL13725.Contig1_All | MGLL | 18.50 | 15.98 | 24.14 | 15.37 | 39.93 | 52.65 | 31.08 | 36.05 | 73.90 | 70.13 | 80.14 | 71.44 | 1.11 | 0.89 | 2.00 |
| CL10135.Contig2_All | NMT | 44.38 | 36.12 | 50.75 | 46.26 | 2.11 | 2.09 | 1.15 | 3.10 | 0.12 | 0.06 | 0.17 | 0.12 | -4.39 | -4.18 | -8.57 |
| CL5796.Contig9_All | NSDHL | 5.94 | 5.27 | 7.69 | 4.85 | 2.37 | 1.87 | 3.15 | 2.10 | 2.29 | 2.34 | 1.93 | 2.61 | -1.32 | -0.05 | -1.37 |
| CL8973.Contig1_All | OLE | 113.05 | 159.74 | 156.40 | 23.00 | 2263.71 | 4367.14 | 3.42 | 2420.57 | 3854.29 | 2165.81 | 2298.65 | 7098.40 | 4.32 | 0.77 | 5.09 |
| CL755.Contig1_All | OPCL1 | 20.62 | 21.09 | 21.62 | 19.15 | 13.56 | 14.47 | 13.53 | 12.69 | 4.40 | 5.05 | 4.85 | 3.30 | -0.60 | -1.62 | -2.23 |
| CL7124.Contig1_All | OPR | 36.03 | 36.67 | 36.44 | 34.98 | 19.37 | 23.56 | 17.93 | 16.61 | 7.47 | 9.50 | 4.97 | 7.95 | -0.90 | -1.37 | -2.27 |
| CL3787.Contig2_All | PAP | 3.69 | 3.38 | 4.45 | 3.24 | 6.41 | 6.55 | 6.21 | 6.48 | 21.88 | 21.11 | 22.74 | 21.78 | 0.80 | 1.77 | 2.57 |
| CL1150.Contig3_All | PCYT2 | 1.77 | 0.84 | 1.92 | 2.55 | 12.45 | 10.16 | 12.66 | 14.52 | 47.29 | 39.59 | 46.32 | 55.97 | 2.81 | 1.93 | 4.74 |
| Unigene43934_All | PDAT | 11.81 | 11.51 | 12.45 | 11.48 | 10.79 | 11.33 | 10.20 | 10.83 | 5.13 | 6.24 | 4.52 | 4.63 | -0.13 | -1.07 | -1.20 |
| CL13909.Contig1_All | PDCT | 36.99 | 39.42 | 29.39 | 42.17 | 13.05 | 11.10 | 13.83 | 14.21 | 1.37 | 1.01 | 1.29 | 1.81 | -1.50 | -3.25 | -4.76 |
| Unigene33843_All | pgsA | 10.57 | 10.22 | 11.63 | 9.87 | 5.66 | 5.87 | 6.24 | 4.86 | 4.61 | 4.06 | 3.94 | 5.84 | -0.90 | -0.29 | -1.20 |
| CL4964.Contig1_All | PLA2 | 8.32 | 7.12 | 11.67 | 6.16 | 7.60 | 8.18 | 5.92 | 8.69 | 17.80 | 4.31 | 16.34 | 32.74 | -0.13 | 1.23 | 1.10 |
| CL10929.Contig1_All | plc | 78.49 | 84.84 | 76.64 | 74.00 | 59.37 | 70.18 | 50.69 | 57.24 | 8.25 | 5.27 | 10.96 | 8.52 | -0.40 | -2.85 | -3.25 |
| CL448.Contig1_All | PLD1_2 | 179.68 | 195.97 | 162.11 | 180.97 | 98.98 | 99.59 | 108.05 | 89.31 | 71.23 | 71.30 | 65.78 | 76.61 | -0.86 | -0.47 | -1.33 |
| Unigene23752_All | PPT | 15.54 | 14.63 | 18.30 | 13.68 | 11.55 | 13.96 | 10.54 | 10.14 | 7.26 | 8.72 | 6.47 | 6.59 | -0.43 | -0.67 | -1.10 |
| Unigene33698_All | PRXL2B | 13.24 | 13.95 | 11.35 | 14.42 | 5.78 | 6.66 | 5.26 | 5.42 | 6.53 | 5.54 | 7.73 | 6.32 | -1.20 | 0.18 | -1.02 |
| CL9914.Contig1_All | psd | 4.80 | 4.42 | 6.52 | 3.45 | 2.35 | 2.79 | 2.40 | 1.85 | 9.06 | 9.51 | 9.99 | 7.67 | -1.03 | 1.95 | 0.92 |
| CL4619.Contig5_All | PTDSS2 | 1.05 | 1.13 | 1.35 | 0.68 | 1.10 | 1.11 | 0.90 | 1.28 | 3.02 | 3.77 | 3.14 | 2.14 | 0.06 | 1.46 | 1.52 |
| CL6439.Contig1_All | PXG | 38.03 | 33.67 | 48.43 | 31.99 | 41.36 | 61.90 | 37.96 | 24.22 | 0.18 | 0.42 | 0.06 | 0.06 | 0.12 | -7.84 | -7.72 |
| Unigene13977_All | SAD | 217.75 | 187.93 | 217.95 | 247.36 | 115.25 | 111.03 | 128.22 | 106.49 | 14.98 | 19.82 | 10.72 | 14.40 | -0.92 | -2.94 | -3.86 |
| CL10085.Contig1_All | SGPL1 | 14.14 | 13.05 | 14.10 | 15.27 | 17.05 | 20.23 | 18.31 | 12.61 | 6.18 | 4.93 | 5.77 | 7.83 | 0.27 | -1.46 | -1.19 |
| CL5840.Contig1_All | SMO1 | 10.94 | 11.40 | 12.26 | 9.15 | 8.18 | 5.55 | 13.76 | 5.24 | 2.80 | 4.27 | 2.68 | 1.44 | -0.42 | -1.55 | -1.97 |
| CL4354.Contig1_All | SMO2 | 11.61 | 10.43 | 13.07 | 11.32 | 4.27 | 4.66 | 4.33 | 3.83 | 1.09 | 1.23 | 1.14 | 0.91 | -1.44 | -1.97 | -3.41 |
| CL10210.Contig2_All | SQLE | 22.30 | 26.15 | 20.02 | 20.74 | 21.01 | 25.44 | 23.09 | 14.49 | 1.23 | 0.70 | 1.74 | 1.25 | -0.09 | -4.09 | -4.18 |
| Unigene16750_All | SUR2 | 31.10 | 30.76 | 34.39 | 28.16 | 32.88 | 37.03 | 29.29 | 32.32 | 120.50 | 94.67 | 131.18 | 135.66 | 0.08 | 1.87 | 1.95 |
| Unigene26956_All | TM7SF2 | 18.50 | 18.67 | 17.63 | 19.21 | 8.84 | 10.01 | 8.84 | 7.67 | 3.38 | 3.30 | 3.75 | 3.08 | -1.07 | -1.39 | -2.45 |
| CL7151.Contig1_All | WSD1 | 6.32 | 6.21 | 5.92 | 6.82 | 12.52 | 13.05 | 13.23 | 11.28 | 25.52 | 21.41 | 27.64 | 27.52 | 0.99 | 1.03 | 2.01 |
| CL5887.Contig1_All | α-CT | 65.34 | 61.60 | 71.63 | 62.80 | 20.99 | 20.75 | 20.00 | 22.23 | 12.93 | 12.27 | 12.51 | 14.02 | -1.64 | -0.70 | -2.34 |
