# Supplementary material for: Identification of genes associated with the biosynthesis of unsaturated fatty acid and oil accumulation in herbaceous peony ‘Hangshao’ (Paeonia lactiflora ‘Hangshao’) seeds based on transcriptome analysis
Source: BMC Genomics. 2021 Feb 1;22:94. doi: 10.1186/s12864-020-07339-7 (PMC7849092; doi:10.1186/s12864-020-07339-7)
Supplement: Supplementary file 13 — Additional file 13: Table S10. DEGs related to fatty acid biosynthesis and oil accumulation in herbaceous peony ‘Hangshao’ seeds [file 12864_2020_7339_MOESM13_ESM.docx]

| **Table S10 DEGs related to fatty acid biosynthesis and oil accumulation in herbaceous peony 'Hangshao' seeds** | | | | | | | | | | | | | | | | | | |
| --- | --- | --- | --- | --- | --- | --- | --- | --- | --- | --- | --- | --- | --- | --- | --- | --- | --- | --- |
| **pathway** | **Enzyme  abbreviation** | **Enzyme full name** | **Gene ID** | **Mean30dFPKM** | **HS30d_1 FPKM** | **HS30d_2 FPKM** | **HS30d_3 FPKM** | **Mean60dFPKM** | **HS60d_1 FPKM** | **HS60d_2 FPKM** | **HS60d_3 FPKM** | **Mean90dFPKM** | **HS90d_1 FPKM** | **HS90d_2 FPKM** | **HS90d_3 FPKM** | **GroupI LOG (60d/30d,2)** | **Group II LOG (90d/60d,2)** | **Group III LOG (90d/30d,2)** |
| **Fatty acid  biosynthsis** | BC | acetyl-CoA carboxylase, biotin carboxylase subunit | CL6932.Contig1_All | 1.17 | 0.82 | 1.42 | 1.28 | 0.89 | 0.91 | 0.69 | 1.07 | 0.01 | 0.01 | 0.01 | 0.01 | -0.40 | -6.48 | -6.87 |
|  |  |  | CL6932.Contig2_All | 6.39 | 5.94 | 6.99 | 6.24 | 1.17 | 1.51 | 1.01 | 0.99 | 0.43 | 0.30 | 0.29 | 0.71 | -2.45 | -1.43 | -3.88 |
|  |  |  | CL6932.Contig3_All | 34.54 | 34.36 | 33.80 | 35.47 | 7.40 | 7.64 | 6.71 | 7.86 | 1.53 | 2.12 | 1.29 | 1.19 | -2.22 | -2.27 | -4.49 |
|  |  |  | CL6932.Contig4_All | 38.01 | 30.87 | 39.35 | 43.80 | 8.20 | 8.04 | 7.00 | 9.55 | 1.80 | 1.91 | 1.63 | 1.86 | -2.21 | -2.19 | -4.40 |
|  |  |  | CL8437.Contig2_All | 5.67 | 5.08 | 6.27 | 5.65 | 4.56 | 3.87 | 5.11 | 4.70 | 0.32 | 0.37 | 0.23 | 0.35 | -0.31 | -3.85 | -4.16 |
|  | α-CT | acetyl-CoA carboxylase carboxyl transferase subunit alpha | CL5887.Contig1_All | 65.34 | 61.60 | 71.63 | 62.80 | 20.99 | 20.75 | 20.00 | 22.23 | 12.93 | 12.27 | 12.51 | 14.02 | -1.64 | -0.70 | -2.34 |
|  |  |  | Unigene30129_All | 2.02 | 3.11 | 0.73 | 2.22 | 0.21 | 0.02 | 0.43 | 0.17 | 0.04 | 0.08 | 0.02 | 0.01 | -3.29 | -2.49 | -5.78 |
|  |  |  | Unigene5237_All | 2.70 | 2.30 | 3.40 | 2.41 | 0.87 | 1.07 | 1.04 | 0.51 | 0.10 | 0.01 | 0.27 | 0.01 | -1.63 | -3.18 | -4.81 |
|  | BCCP | acetyl-CoA carboxylase biotin carboxyl carrier protein | CL14917.Contig1_All | 39.24 | 32.45 | 49.49 | 35.77 | 9.23 | 8.97 | 7.21 | 11.52 | 2.08 | 2.28 | 1.53 | 2.43 | -2.09 | -2.15 | -4.24 |
|  |  |  | CL14917.Contig2_All | 39.97 | 36.71 | 45.87 | 37.34 | 8.50 | 8.76 | 7.61 | 9.14 | 2.23 | 1.95 | 2.15 | 2.58 | -2.23 | -1.93 | -4.17 |
|  |  |  | CL6718.Contig4_All | 0.24 | 0.53 | 0.18 | 0.01 | 0.23 | 0.53 | 0.16 | 0.01 | 2.81 | 3.23 | 3.21 | 2.00 | -0.04 | 3.59 | 3.55 |
|  |  |  | CL750.Contig1_All | 3.68 | 4.03 | 0.96 | 6.06 | 0.49 | 0.45 | 0.82 | 0.19 | 0.42 | 1.14 | 0.10 | 0.01 | -2.92 | -0.22 | -3.14 |
|  |  |  | CL750.Contig2_All | 6.82 | 7.36 | 4.41 | 8.68 | 1.52 | 2.09 | 1.52 | 0.96 | 1.21 | 1.81 | 1.22 | 0.59 | -2.16 | -0.34 | -2.50 |
|  |  |  | CL750.Contig6_All | 3.33 | 4.22 | 3.33 | 2.43 | 0.85 | 1.09 | 1.04 | 0.41 | 0.58 | 0.52 | 0.61 | 0.60 | -1.97 | -0.55 | -2.53 |
|  |  |  | Unigene23603_All | 49.45 | 48.49 | 46.97 | 52.90 | 19.87 | 19.72 | 20.34 | 19.55 | 7.34 | 9.97 | 5.95 | 6.10 | -1.32 | -1.44 | -2.75 |
|  | MCAT | [acyl-carrier-protein] S-malonyltransferase | Unigene20202_All | 20.44 | 21.65 | 20.62 | 19.04 | 6.68 | 8.13 | 4.15 | 7.75 | 4.60 | 3.79 | 5.06 | 4.94 | -1.61 | -0.54 | -2.15 |
|  | KASIII | 3-oxoacyl-[acyl-carrier-protein] synthase III | Unigene37345_All | 45.76 | 44.67 | 50.51 | 42.09 | 17.35 | 16.44 | 17.95 | 17.65 | 14.05 | 16.20 | 11.73 | 14.23 | -1.40 | -0.30 | -1.70 |
|  | KAR | 3-oxoacyl-[acyl-carrier protein] reductase | Unigene20183_All | 173.61 | 165.19 | 191.42 | 164.23 | 80.67 | 76.07 | 71.11 | 94.84 | 46.07 | 45.67 | 38.84 | 53.71 | -1.11 | -0.81 | -1.91 |
|  |  |  | CL2376.Contig1_All | 3.84 | 3.71 | 0.74 | 7.08 | 147.42 | 154.37 | 214.87 | 73.01 | 166.84 | 1.39 | 497.19 | 1.94 | 5.26 | 0.18 | 5.44 |
|  |  |  | CL2376.Contig2_All | 2.03 | 1.10 | 4.39 | 0.59 | 112.63 | 99.65 | 85.28 | 152.96 | 1071.11 | ###### | 448.97 | ###### | 5.80 | 3.25 | 9.05 |
|  |  |  | CL2376.Contig3_All | 1.38 | 0.21 | 3.54 | 0.39 | 144.04 | 1.44 | 427.73 | 2.94 | 0.17 | 0.50 | 0.01 | 0.01 | 6.71 | -9.70 | -2.99 |
|  |  |  | CL2573.Contig1_All | 2.49 | 2.56 | 1.87 | 3.05 | 1.23 | 1.36 | 1.30 | 1.03 | 0.97 | 1.48 | 0.72 | 0.70 | -1.02 | -0.35 | -1.37 |
|  |  |  | CL7002.Contig5_All | 0.46 | 0.22 | 0.39 | 0.76 | 0.39 | 0.43 | 0.01 | 0.72 | 5.77 | 3.54 | 8.55 | 5.22 | -0.24 | 3.90 | 3.66 |
|  |  |  | CL7002.Contig6_All | 0.77 | 0.67 | 0.93 | 0.71 | 0.55 | 0.54 | 0.54 | 0.56 | 3.75 | 3.88 | 3.32 | 4.05 | -0.49 | 2.78 | 2.28 |
|  |  |  | CL7002.Contig7_All | 0.05 | 0.01 | 0.14 | 0.01 | 0.03 | 0.01 | 0.01 | 0.06 | 0.85 | 0.85 | 0.65 | 1.05 | -1.00 | 4.99 | 3.99 |
|  | HAD | 3-hydroxyacyl-[acyl-carrier-protein] dehydratase | CL1701.Contig1_All | 7.53 | 8.73 | 7.33 | 6.52 | 1.52 | 1.76 | 1.85 | 0.95 | 0.14 | 0.05 | 0.10 | 0.26 | -2.31 | -3.48 | -5.78 |
|  |  |  | CL1701.Contig2_All | 7.34 | 7.63 | 7.03 | 7.35 | 1.66 | 1.29 | 1.72 | 1.96 | 0.81 | 1.24 | 0.42 | 0.77 | -2.15 | -1.03 | -3.18 |
|  |  |  | CL1701.Contig4_All | 18.02 | 21.58 | 15.93 | 16.56 | 1.57 | 1.96 | 0.92 | 1.82 | 0.48 | 0.46 | 0.33 | 0.66 | -3.52 | -1.70 | -5.22 |
|  |  |  | CL1701.Contig5_All | 43.29 | 48.33 | 40.85 | 40.68 | 13.69 | 14.10 | 13.20 | 13.76 | 13.45 | 15.35 | 11.76 | 13.23 | -1.66 | -0.03 | -1.69 |
|  | EAR | enoyl-[acyl-carrier protein] reductase I | CL5466.Contig2_All | 18.52 | 18.07 | 19.01 | 18.47 | 2.86 | 3.70 | 2.43 | 2.45 | 1.12 | 0.01 | 1.55 | 1.79 | -2.69 | -1.36 | -4.05 |
|  |  |  | CL7296.Contig2_All | 53.95 | 30.18 | 68.60 | 63.06 | 6.21 | 4.83 | 3.35 | 10.46 | 0.07 | 0.15 | 0.04 | 0.01 | -3.12 | -6.54 | -9.66 |
|  |  |  | CL7296.Contig3_All | 27.18 | 44.10 | 17.27 | 20.16 | 3.70 | 3.58 | 4.29 | 3.23 | 0.01 | 0.01 | 0.01 | 0.01 | -2.88 | -8.53 | -11.41 |
|  | KASII | 3-oxoacyl-[acyl-carrier-protein] synthase II | CL14889.Contig1_All | 62.96 | 55.83 | 64.22 | 68.82 | 28.04 | 32.51 | 24.31 | 27.30 | 3.97 | 3.78 | 3.54 | 4.60 | -1.17 | -2.82 | -3.99 |
|  |  |  | Unigene16003_All | 1.57 | 1.56 | 2.20 | 0.95 | 0.54 | 0.59 | 0.44 | 0.59 | 0.62 | 0.83 | 0.36 | 0.68 | -1.54 | 0.21 | -1.33 |
|  |  |  | Unigene29386_All | 58.08 | 55.01 | 61.69 | 57.55 | 17.73 | 19.04 | 15.08 | 19.08 | 7.36 | 6.42 | 5.96 | 9.70 | -1.71 | -1.27 | -2.98 |
|  |  |  | Unigene34228_All | 5.37 | 8.45 | 4.06 | 3.60 | 0.37 | 0.06 | 0.06 | 0.99 | 0.03 | 0.01 | 0.07 | 0.01 | -3.86 | -3.62 | -7.48 |
|  | FATA | fatty acyl-ACP thioesterase A | CL14443.Contig1_All | 45.73 | 44.91 | 45.88 | 46.39 | 21.26 | 24.00 | 18.52 | 21.27 | 11.71 | 13.51 | 8.80 | 12.81 | -1.10 | -0.86 | -1.97 |
|  | FATB | fatty acyl-ACP thioesterase B | Unigene1453_All | 0.21 | 0.04 | 0.58 | 0.00 | 0.22 | 0.33 | 0.25 | 0.08 | 0.01 | 0.01 | 0.01 | 0.01 | 0.09 | -4.46 | -4.37 |
|  |  |  | Unigene26774_All | 75.44 | 69.36 | 83.51 | 73.45 | 42.12 | 40.97 | 40.73 | 44.65 | 32.45 | 31.01 | 38.44 | 27.91 | -0.84 | -0.38 | -1.22 |
| **Fatty acid  elongation** | LACS | long-chain acyl-CoA synthetase | CL8536.Contig1_All | 1.02 | 0.97 | 1.17 | 0.93 | 1.01 | 1.19 | 0.69 | 1.15 | 2.42 | 2.47 | 1.86 | 2.93 | -0.02 | 1.26 | 1.24 |
|  |  |  | Unigene13215_All | 0.10 | 0.13 | 0.01 | 0.16 | 0.04 | 0.01 | 0.07 | 0.05 | 0.62 | 0.83 | 0.29 | 0.74 | -1.21 | 3.84 | 2.63 |
|  |  |  | Unigene19998_All | 35.32 | 29.19 | 35.53 | 41.23 | 17.81 | 20.26 | 16.80 | 16.37 | 14.63 | 17.17 | 13.85 | 12.86 | -0.99 | -0.28 | -1.27 |
|  | KCS | 3-ketoacyl-CoA synthase | CL14039.Contig2_All | 54.97 | 56.09 | 56.93 | 51.89 | 25.26 | 29.88 | 23.00 | 22.90 | 0.96 | 2.07 | 0.41 | 0.39 | -1.12 | -4.72 | -5.84 |
|  |  |  | CL14705.Contig1_All | 3.89 | 6.80 | 2.86 | 2.01 | 0.13 | 0.23 | 0.06 | 0.09 | 0.13 | 0.06 | 0.03 | 0.29 | -4.94 | 0.00 | -4.94 |
|  |  |  | CL14705.Contig2_All | 11.89 | 13.41 | 13.12 | 9.14 | 0.10 | 0.13 | 0.05 | 0.11 | 0.27 | 0.21 | 0.03 | 0.56 | -6.94 | 1.46 | -5.48 |
|  |  |  | CL8909.Contig2_All | 4.07 | 4.97 | 3.70 | 3.53 | 1.21 | 1.17 | 1.37 | 1.08 | 1.77 | 2.02 | 1.48 | 1.82 | -1.75 | 0.56 | -1.20 |
|  |  |  | CL8909.Contig3_All | 4.27 | 5.30 | 3.26 | 4.25 | 1.25 | 1.36 | 1.38 | 1.01 | 1.64 | 2.05 | 1.45 | 1.41 | -1.77 | 0.39 | -1.38 |
|  |  |  | Unigene1248_All | 0.68 | 0.75 | 0.58 | 0.70 | 0.11 | 0.24 | 0.05 | 0.05 | 0.01 | 0.01 | 0.01 | 0.01 | -2.58 | -3.50 | -6.08 |
|  |  |  | Unigene20808_All | 0.91 | 0.93 | 1.08 | 0.73 | 0.05 | 0.07 | 0.01 | 0.07 | 0.01 | 0.01 | 0.01 | 0.01 | -4.19 | -2.32 | -6.51 |
|  |  |  | Unigene30412_All | 7.00 | 6.65 | 5.09 | 9.25 | 4.14 | 5.43 | 3.20 | 3.79 | 2.15 | 1.37 | 2.60 | 2.49 | -0.76 | -0.94 | -1.70 |
|  |  |  | Unigene30555_All | 1.23 | 1.43 | 1.14 | 1.12 | 1.25 | 1.13 | 1.48 | 1.13 | 0.02 | 0.01 | 0.03 | 0.01 | 0.02 | -6.22 | -6.21 |
|  |  |  | Unigene33652_All | 2.55 | 2.62 | 2.97 | 2.06 | 3.86 | 5.04 | 2.56 | 3.99 | 0.57 | 0.19 | 0.65 | 0.88 | 0.60 | -2.75 | -2.15 |
|  |  |  | Unigene37896_All | 0.50 | 0.52 | 0.41 | 0.57 | 0.02 | 0.01 | 0.01 | 0.04 | 0.05 | 0.01 | 0.09 | 0.05 | -4.64 | 1.32 | -3.32 |
|  |  |  | Unigene40102_All | 1.54 | 2.01 | 1.30 | 1.30 | 0.83 | 0.63 | 0.88 | 0.99 | 0.62 | 0.56 | 0.71 | 0.58 | -0.88 | -0.43 | -1.32 |
|  |  |  | Unigene82243_All | 27.49 | 29.62 | 27.55 | 25.31 | 16.03 | 18.32 | 16.48 | 13.29 | 13.46 | 13.52 | 8.87 | 18.00 | -0.78 | -0.25 | -1.03 |
|  | KCR | very-long-chain 3-oxoacyl-CoA reductase | CL15103.Contig1_All | 392.23 | 471.25 | 311.95 | 393.50 | 791.97 | 822.18 | 923.26 | 630.48 | 17.13 | 17.08 | 15.90 | 18.40 | 1.01 | -5.53 | -4.52 |
|  |  |  | CL15103.Contig2_All | 2.17 | 1.58 | 2.87 | 2.07 | 2.74 | 3.07 | 3.35 | 1.80 | 0.14 | 0.06 | 0.11 | 0.24 | 0.33 | -4.33 | -3.99 |
|  |  |  | Unigene17156_All | 0.88 | 0.77 | 0.87 | 1.01 | 0.23 | 0.46 | 0.11 | 0.12 | 0.03 | 0.01 | 0.06 | 0.01 | -1.94 | -3.11 | -5.05 |
|  |  |  | Unigene7734_All | 2.23 | 2.02 | 2.51 | 2.15 | 0.97 | 1.42 | 0.79 | 0.69 | 0.25 | 0.29 | 0.16 | 0.29 | -1.20 | -1.97 | -3.17 |
|  | HCD | very-long-chain (3R)-3-hydroxyacyl-CoA dehydratase | CL6818.Contig4_All | 11.90 | 12.34 | 12.95 | 10.41 | 4.64 | 5.24 | 4.37 | 4.31 | 1.04 | 1.83 | 0.49 | 0.81 | -1.36 | -2.15 | -3.51 |
|  |  |  | CL6953.Contig3_All | 0.89 | 0.86 | 0.77 | 1.05 | 0.85 | 1.64 | 0.51 | 0.39 | 18.97 | 3.52 | 34.79 | 18.61 | -0.08 | 4.49 | 4.41 |
|  |  |  | Unigene33616_All | 4.94 | 3.02 | 4.63 | 7.18 | 4.04 | 5.23 | 4.36 | 2.54 | 1.32 | 2.36 | 0.75 | 0.85 | -0.29 | -1.62 | -1.90 |
|  | ECR | very-long-chain enoyl-CoA reductase | CL3941.Contig2_All | 2.54 | 1.55 | 3.07 | 3.01 | 3.79 | 3.72 | 5.95 | 1.70 | 17.74 | 17.71 | 23.31 | 12.20 | 0.58 | 2.23 | 2.80 |
|  |  |  | CL3941.Contig3_All | 13.23 | 10.67 | 13.49 | 15.53 | 15.63 | 16.47 | 17.54 | 12.89 | 42.81 | 48.80 | 43.82 | 35.80 | 0.24 | 1.45 | 1.69 |
|  |  |  | Unigene30380_All | 34.34 | 36.19 | 37.18 | 29.64 | 16.01 | 17.02 | 14.76 | 16.26 | 7.09 | 6.67 | 5.30 | 9.30 | -1.10 | -1.18 | -2.28 |
|  | PLA2 | phospholipase A2 | CL12271.Contig1_All | 11.52 | 11.88 | 10.99 | 11.69 | 7.66 | 7.35 | 6.92 | 8.71 | 19.69 | 20.09 | 21.44 | 17.55 | -0.59 | 1.36 | 0.77 |
|  |  |  | CL12271.Contig2_All | 1.48 | 0.98 | 2.03 | 1.43 | 1.70 | 1.72 | 2.62 | 0.77 | 4.52 | 5.98 | 3.31 | 4.27 | 0.20 | 1.41 | 1.61 |
|  |  |  | CL4542.Contig1_All | 4.55 | 4.45 | 6.02 | 3.17 | 1.02 | 1.55 | 0.01 | 1.50 | 4.58 | 5.59 | 5.66 | 2.49 | -2.16 | 2.17 | 0.01 |
|  |  |  | CL4964.Contig1_All | 8.32 | 7.12 | 11.67 | 6.16 | 7.60 | 8.18 | 5.92 | 8.69 | 17.80 | 4.31 | 16.34 | 32.74 | -0.13 | 1.23 | 1.10 |
|  |  |  | CL4964.Contig3_All | 12.82 | 12.49 | 11.26 | 14.71 | 10.17 | 10.04 | 12.56 | 7.90 | 20.90 | 35.04 | 21.00 | 6.66 | -0.33 | 1.04 | 0.71 |
|  |  |  | Unigene27449_All | 5.90 | 4.67 | 9.71 | 3.31 | 5.26 | 4.26 | 3.47 | 8.04 | 0.09 | 0.09 | 0.01 | 0.18 | -0.17 | -5.82 | -5.98 |
|  |  |  | Unigene44246_All | 1.19 | 0.55 | 2.06 | 0.95 | 0.93 | 1.36 | 0.60 | 0.84 | 0.20 | 0.26 | 0.17 | 0.18 | -0.35 | -2.20 | -2.54 |
| **Biosynthesis of  unsaturated fatty acid** | SAD | acyl-[acyl-carrier-protein] desaturase | Unigene11242_All | 0.05 | 0.01 | 0.01 | 0.13 | 0.01 | 0.01 | 0.01 | 0.01 | 0.52 | 0.70 | 0.01 | 0.84 | -2.32 | 5.69 | 3.37 |
|  |  |  | Unigene12815_All | 0.13 | 0.01 | 0.01 | 0.36 | 0.01 | 0.01 | 0.01 | 0.01 | 0.55 | 1.16 | 0.01 | 0.48 | -3.66 | 5.78 | 2.12 |
|  |  |  | Unigene13977_All | 217.75 | 187.93 | 217.95 | 247.36 | 115.25 | 111.03 | 128.22 | 106.49 | 14.98 | 19.82 | 10.72 | 14.40 | -0.92 | -2.94 | -3.86 |
|  |  |  | Unigene32879_All | 130.52 | 169.89 | 113.15 | 108.53 | 86.35 | 63.26 | 128.81 | 66.97 | 20.69 | 5.48 | 16.71 | 39.87 | -0.60 | -2.06 | -2.66 |
|  |  |  | Unigene39711_All | 35.78 | 47.89 | 27.46 | 31.99 | 24.31 | 14.32 | 40.39 | 18.23 | 4.95 | 5.06 | 1.46 | 8.34 | -0.56 | -2.30 | -2.85 |
|  |  |  | Unigene4234_All | 49.24 | 43.27 | 64.17 | 40.27 | 34.96 | 40.49 | 29.20 | 35.18 | 6.66 | 5.35 | 8.06 | 6.57 | -0.49 | -2.39 | -2.89 |
|  | FAD2 | omega-6 fatty acid desaturase / acyl-lipid omega-6 desaturase (Delta-12 desaturase) | CL6171.Contig1_All | 2.36 | 1.26 | 2.41 | 3.41 | 0.82 | 0.54 | 0.79 | 1.12 | 0.04 | 0.00 | 0.00 | 0.12 | -1.53 | -4.35 | -5.88 |
|  |  |  | CL6171.Contig2_All | 2.32 | 2.84 | 0.40 | 3.72 | 4.44 | 4.16 | 7.11 | 2.06 | 0.08 | 0.00 | 0.22 | 0.02 | 0.94 | -5.80 | -4.86 |
|  |  |  | CL6171.Contig3_All | 1.39 | 0.48 | 1.38 | 2.32 | 2.89 | 2.16 | 1.89 | 4.61 | 0.15 | 0.12 | 0.06 | 0.26 | 1.05 | -4.30 | -3.25 |
|  |  |  | CL6171.Contig4_All | 5.72 | 3.10 | 2.24 | 11.82 | 11.62 | 10.05 | 11.69 | 13.12 | 0.24 | 0.40 | 0.17 | 0.16 | 1.02 | -5.58 | -4.56 |
|  |  |  | CL2636.Contig1_All | 3.29 | 2.26 | 2.87 | 4.75 | 1.04 | 1.92 | 0.01 | 1.18 | 0.08 | 0.13 | 0.09 | 0.01 | -1.67 | -3.76 | -5.42 |
|  |  |  | CL2636.Contig2_All | 4.14 | 2.56 | 3.16 | 6.69 | 0.74 | 1.50 | 0.07 | 0.64 | 0.03 | 0.08 | 0.01 | 0.01 | -2.49 | -4.47 | -6.96 |
|  |  |  | CL2636.Contig3_All | 108.58 | 72.38 | 103.25 | 150.12 | 46.85 | 47.61 | 27.48 | 65.45 | 1.18 | 1.58 | 0.71 | 1.25 | -1.21 | -5.31 | -6.52 |
|  |  |  | CL2636.Contig4_All | 618.85 | 785.17 | 405.49 | 665.90 | 292.97 | 332.46 | 217.02 | 329.44 | 2.68 | 3.95 | 1.36 | 2.72 | -1.08 | -6.77 | -7.85 |
|  |  |  | Unigene37303_All | 39.12 | 40.07 | 36.53 | 40.77 | 37.37 | 40.67 | 35.02 | 36.43 | 1.41 | 1.70 | 1.10 | 1.44 | -0.07 | -4.72 | -4.79 |
|  | FAD3 | acyl-lipid omega-3 desaturase | CL2686.Contig5_All | 108.55 | 54.50 | 270.48 | 0.66 | 336.21 | 269.82 | 108.96 | 629.84 | 0.51 | 0.01 | 0.81 | 0.71 | 1.63 | -9.36 | -7.73 |
|  |  |  | CL2686.Contig3_All | 0.77 | 0.27 | 0.95 | 1.09 | 0.26 | 0.09 | 0.61 | 0.09 | 0.04 | 0.10 | 0.01 | 0.01 | -1.55 | -2.72 | -4.27 |
|  |  |  | CL2686.Contig8_All | 415.93 | 410.47 | 311.06 | 526.27 | 44.16 | 32.91 | 98.65 | 0.92 | 0.77 | 1.56 | 0.32 | 0.42 | -3.24 | -5.85 | -9.08 |
|  |  |  | CL2686.Contig6_All | 480.23 | 439.22 | 520.31 | 481.17 | 335.31 | 262.84 | 177.35 | 565.75 | 0.92 | 1.76 | 0.39 | 0.61 | -0.52 | -8.51 | -9.03 |
|  |  |  | CL2686.Contig7_All | 0.88 | 0.42 | 0.79 | 1.42 | 0.16 | 0.41 | 0.01 | 0.05 | 0.07 | 0.15 | 0.05 | 0.01 | -2.48 | -1.16 | -3.65 |
|  | FAD7 | chloroplastic;acyl-lipid omega-3 desaturase | CL13349.Contig2_All | 9.97 | 10.92 | 9.05 | 9.93 | 9.04 | 9.30 | 9.31 | 8.51 | 0.40 | 0.46 | 0.32 | 0.41 | -0.14 | -4.51 | -4.65 |
|  |  |  | CL13349.Contig1_All | 7.88 | 7.02 | 7.37 | 9.24 | 6.70 | 5.87 | 7.51 | 6.72 | 0.14 | 0.30 | 0.01 | 0.11 | -0.23 | -5.58 | -5.81 |
|  | FAD8 | chloroplastic;acyl-lipid omega-3 desaturase | Unigene34203_All | 41.43 | 50.53 | 30.32 | 43.45 | 31.54 | 22.37 | 38.39 | 33.85 | 0.01 | 0.01 | 0.01 | 0.01 | -0.39 | -11.62 | -12.02 |
|  |  |  | Unigene34202_All | 39.93 | 46.41 | 29.21 | 44.17 | 28.97 | 19.22 | 35.19 | 32.51 | 0.02 | 0.04 | 0.01 | 0.01 | -0.46 | -10.50 | -10.96 |
| **Triacylglycerol assembly** | GPAT | glycerol-3-phosphate acyltransferase | CL12348.Contig2_All | 2.13 | 1.66 | 2.44 | 2.29 | 0.64 | 0.75 | 0.56 | 0.60 | 0.63 | 0.60 | 0.29 | 0.99 | -1.74 | -0.02 | -1.77 |
|  |  |  | CL12348.Contig3_All | 2.00 | 1.88 | 2.37 | 1.74 | 0.84 | 0.69 | 0.79 | 1.05 | 0.43 | 0.49 | 0.36 | 0.44 | -1.24 | -0.97 | -2.22 |
|  |  |  | Unigene30381_All | 7.74 | 7.02 | 9.91 | 6.29 | 3.60 | 4.20 | 2.84 | 3.77 | 2.27 | 3.23 | 2.09 | 1.50 | -1.10 | -0.66 | -1.77 |
|  |  |  | Unigene38234_All | 0.05 | 0.09 | 0.06 | 0.01 | 6.64 | 9.98 | 7.55 | 2.40 | 0.02 | 0.03 | 0.01 | 0.01 | 6.96 | -8.64 | -1.68 |
|  | LPAAT | 1-acyl-sn-glycerol-3-phosphate acyltransferase | Unigene40734_All | 7.36 | 8.05 | 7.20 | 6.84 | 4.68 | 4.48 | 4.38 | 5.17 | 3.57 | 3.87 | 2.83 | 4.00 | -0.65 | -0.39 | -1.05 |
|  |  |  | CL10192.Contig1_All | 6.96 | 8.26 | 8.53 | 4.09 | 3.36 | 2.94 | 5.49 | 1.66 | 2.42 | 2.76 | 2.35 | 2.14 | -1.05 | -0.48 | -1.53 |
|  |  |  | CL3672.Contig1_All | 13.29 | 14.10 | 12.76 | 13.01 | 14.73 | 21.44 | 11.38 | 11.38 | 41.96 | 51.15 | 38.36 | 36.36 | 0.15 | 1.51 | 1.66 |
|  |  |  | CL3672.Contig6_All | 12.93 | 13.72 | 13.64 | 11.43 | 13.23 | 19.49 | 8.00 | 12.20 | 35.08 | 44.12 | 30.04 | 31.08 | 0.03 | 1.41 | 1.44 |
|  | PAP | phosphatidate phosphatase | CL13997.Contig1_All | 1.96 | 1.92 | 2.20 | 1.75 | 3.62 | 3.93 | 2.93 | 3.99 | 8.89 | 4.41 | 10.55 | 11.70 | 0.89 | 1.30 | 2.18 |
|  |  |  | CL13997.Contig3_All | 5.47 | 5.19 | 6.36 | 4.85 | 6.62 | 6.24 | 7.26 | 6.35 | 13.26 | 13.44 | 12.01 | 14.34 | 0.28 | 1.00 | 1.28 |
|  |  |  | CL3787.Contig1_All | 4.17 | 3.96 | 4.75 | 3.80 | 7.30 | 7.56 | 6.71 | 7.62 | 19.72 | 15.92 | 24.74 | 18.49 | 0.81 | 1.43 | 2.24 |
|  |  |  | CL3787.Contig2_All | 3.69 | 3.38 | 4.45 | 3.24 | 6.41 | 6.55 | 6.21 | 6.48 | 21.88 | 21.11 | 22.74 | 21.78 | 0.80 | 1.77 | 2.57 |
|  |  |  | CL3787.Contig3_All | 0.14 | 0.01 | 0.41 | 0.01 | 0.01 | 0.01 | 0.01 | 0.01 | 5.03 | 7.19 | 0.04 | 7.85 | -3.84 | 8.97 | 5.13 |
|  | DGAT | diacylglycerol O-acyltransferase | CL6160.Contig2_All | 3.37 | 2.73 | 4.95 | 2.44 | 4.41 | 4.14 | 5.10 | 3.99 | 8.88 | 7.68 | 10.06 | 8.90 | 0.39 | 1.01 | 1.40 |
|  | PDAT | phospholipid:diacylglycerol acyltransferase | Unigene43934_All | 11.81 | 11.51 | 12.45 | 11.48 | 10.79 | 11.33 | 10.20 | 10.83 | 5.13 | 6.24 | 4.52 | 4.63 | -0.13 | -1.07 | -1.20 |
|  |  |  | CL14912.Contig1_All | 0.18 | 0.28 | 0.15 | 0.11 | 0.90 | 0.38 | 1.26 | 1.07 | 0.30 | 0.21 | 0.37 | 0.31 | 2.33 | -1.61 | 0.72 |
|  | PDCT | Phosphatidylcholine:diacylglycerol cholinephosphotransferase 1 | CL13909.Contig1_All | 36.99 | 39.42 | 29.39 | 42.17 | 13.05 | 11.10 | 13.83 | 14.21 | 1.37 | 1.01 | 1.29 | 1.81 | -1.50 | -3.25 | -4.76 |
| **Lipid storage** | OLE | Oleosin | CL8973.Contig1_All | 113.05 | 159.74 | 156.40 | 23.00 | ###### | ###### | 3.42 | ###### | 3854.29 | ###### | ###### | ###### | 4.32 | 0.77 | 5.09 |
|  |  |  | CL8973.Contig2_All | 65.98 | 46.55 | 19.98 | 131.41 | 720.68 | 11.11 | ###### | 512.91 | 1234.71 | ###### | ###### | 43.55 | 3.45 | 0.78 | 4.23 |
|  |  |  | CL8973.Contig3_All | 145.30 | 148.01 | 124.06 | 163.83 | ###### | ###### | ###### | ###### | 3093.21 | ###### | ###### | ###### | 3.60 | 0.81 | 4.41 |
|  |  |  | CL8973.Contig4_All | 82.26 | 90.62 | 93.91 | 62.26 | 215.24 | 416.98 | 33.19 | 195.55 | 408.50 | 138.83 | 200.97 | 885.71 | 1.39 | 0.92 | 2.31 |
|  |  |  | Unigene23397_All | 159.36 | 168.52 | 153.08 | 156.48 | 558.79 | 764.75 | 467.33 | 444.29 | 546.49 | 649.40 | 418.10 | 571.96 | 1.81 | -0.03 | 1.78 |
|  |  |  | Unigene26649_All | 250.44 | 300.36 | 210.82 | 240.14 | ###### | ###### | ###### | 933.91 | 675.81 | 703.65 | 689.74 | 634.03 | 2.05 | -0.62 | 1.43 |
|  | CLO | Caleosin | CL6205.Contig10_All | 435.85 | 382.73 | 418.00 | 506.82 | 673.13 | 655.64 | 735.15 | 628.60 | 1272.66 | ###### | 976.72 | ###### | 0.63 | 0.92 | 1.55 |
|  |  |  | CL6205.Contig7_All | 0.76 | 0.73 | 0.83 | 0.72 | 2.03 | 1.13 | 3.61 | 1.34 | 0.44 | 0.00 | 0.38 | 0.93 | 1.42 | -2.21 | -0.80 |
|  |  |  | CL6205.Contig8_All | 14.97 | 15.13 | 15.22 | 14.56 | 19.04 | 16.49 | 26.24 | 14.39 | 58.17 | 50.29 | 67.06 | 57.15 | 0.35 | 1.61 | 1.96 |
|  |  |  | CL6205.Contig9_All | 0.48 | 0.10 | 0.86 | 0.49 | 1.90 | 1.98 | 0.64 | 3.08 | 1.46 | 2.00 | 0.63 | 1.76 | 1.97 | -0.38 | 1.60 |
