# Supplementary material for: Identification of genes associated with the biosynthesis of unsaturated fatty acid and oil accumulation in herbaceous peony ‘Hangshao’ (Paeonia lactiflora ‘Hangshao’) seeds based on transcriptome analysis
Source: BMC Genomics. 2021 Feb 1;22:94. doi: 10.1186/s12864-020-07339-7 (PMC7849092; doi:10.1186/s12864-020-07339-7)
Supplement: Supplementary file 14 — Additional file 14: Table S11. Gene sequences of 17 BCCP in herbaceous peony ‘Hangshao’ from tanscriptome database [file 12864_2020_7339_MOESM14_ESM.docx]

**Table S11 Gene sequence of 17 BCCP in *Paeonia lactiflora* 'Hangshao' from tanscriptome database**

| NO. | Gene ID | Gene Sequence |
| --- | --- | --- |
| 01 | CL14917.Contig1_All | GCCACACAAGAAACAGAAAACTGCATGCATTGTTACTTAATACAAATTAAAAATTGAAGAACAAAATTATTACTTGAAACTATTGTCAAATGTATAATACCAATTACATTCATTCACCGGCTACACTTATGTCCCTCAATTGCACAATGCAACGAGACAAACAAAAAGATGAAAAGAATTAAAAAAAAATGCCAAAATGCAAACCTTGATCTCTCCCAACAAGGTTGCCTCAACTAAACTACAGCAGAAATATAGGAGACAACCCTACTTGATTCCATGAAATGATGGCAACACTGCCAAAAGAGCATCTCCATAACCAACACCCTCTCCTTCATCAAAGAGAAGCTTCAATACTTCTCCAGCCACATCCGACTTCACAGGAAGTTCGGTGCCAAACTGATCCACATAACCTATTACTTGCCCTTCTTTAATCACATCACCCTCTTTACAAATAGGTGGTTGCTTCTTTCCTTTCATTGTCCTCCCCCTTCGAAACGACCCAACCGTTGGAGATGACACTAGAACATAAGCATTAGATCCAGAAGCTTCAAGAGCCTCTAATTTTGAGGATTTTGCCACGGCAACATTTTTAAATGGATTGATTTTCTCTGAAGATGATTTTGGTGGAGAAGCTGGTGAGGCAGCAGGAGCTGATTCAATCATCGGTTTAGATGGAACAGGTGGTGGAGTTGAAGGTGAAATGAAACTAGTTGGAGTTTTTGTGACTCCAATGTTACGCTTTAGATGCATTTCAAAGTCTCCAACCTTCATCTTTAGCTCAGCAACTTCAGTCCCATCACAGACCTCCAGAATCAATGCCTCAAATCCATTGGGAAAAATAGGACTTGCTAGAGTATTCTTCTCCAATGAGCTATCTAACTCACCAACAGACTTGGCTGTTTTGGCAGGTTCGGATGTATTTACACATGACACTAGCATTCCCTTGCGCTGTACTGGAAGAGAAGAGATTTTTCCACCAACTGTGAGGCCTTGGATATGTAGTTTACTTGCAAATGAACAGGTGGCACTATACATAGAAACCATGCCAGGCCTCTCAAATAAGCTCCGCATATGTGAGACAGTTCCTACAGAATAGTGAAAGGATCGGAGAACTGCAGCAGACTCCATGAATGGAATGAGCAGAGATTCGAAAAAAATCGGAGAGAGTGGGCAAAATGTAGGTGGAGGTTTGAAGGGGAATTATTATTAAAGGAGAAACGAGTTAGGTTAGAGAGTGATGGATTTATAGTGATTTAGACGAGCAGGTATGAGTTTGGTTAGCACAGTTGGAAGGGTGGTGTCAGGTGGTGTCTTCGAAATGTCCGCCTATCGCATCAGTTTCTCCACCCTCCCCGTCATAAGTTCGTTTTAGTTTCCAC |
| 02 | CL14917.Contig2_All | TGGAAACTAAAACGAACTTATGACGGGGAGGGTGGAGAAACTGATGCGATAGGCGGACATTTCGAAGACACCACCTGACACCACCCTTCCAACTGTGCTAACCAAACTCATACCTGCTCGTCTAAATCACTATAAATCCATCACTCTCTAACCTAACTCGTTTCTCCTTTAATAATAATTCCCCTTCAAACCTCCACCTACATTTTGCCCACTCTCTCCGATTTTTTTCGAATCTCTGCTCATTCCATTCATGGAGTCTGCTGCAGTTCTCCGATCCTTTCACTATTCTGTAGGAACTGTCTCACATATGCGGAGCTTATTTGAGAGGCCTGGCATGGTTTCTATGTATAGTGCCACCTGTTCATTTGCAAGTAAACTACATATCCAAGGCCTCACAGTTGGTGGAAAAATCTCTTCTCTTCCAGTACAGCGCAAGGGAATGCTAGTGTCATGTGTAAATACATCCGAACCTGCCAAAACAGCCAAGTCTGTTGGTGAGTTAGATAGCTCATTGGAGAAGAATACTCTAGCAAGTCCTATTTTTCCCAATGGATTTGAGGCATTGATTCTGGAGGTCTGTGATGGGACTGAAGTTGCTGAGCTAAAGATGAAGGTTGGAGACTTTGAAATGCATCTAAAGCGTAACATTGGAGTCACAAAAACTCCAACTAGTTTCATTTCACCTTCAACTCCACCACCTGTTCCATCTAAACCGATGATTGAATCAGCTCCTGCTGCCTCACCAGCTTCTCCACCAAAATCAGCTTCAGAGAAAATCAATCCATTTAAAAATGTTGCCGTGGCAAAATCCTCAAAATTAGAGGCTCTTGAAGCTTCTGGATCTAATGCTTATGTTCTAGTGTCATCTCCAACGGTTGGGTCGTTTCGAAGGGGGAGGACAATGAAAGGAAAGAAGCAACCACCTATTTGTAAAGAGGGTGATGTGATTAAAGAAGGGCAAGTAATAGGTTATGTGGATCAGTTTGGCACCGAACTTCCTGTGAAGTCGGATGTGGCTGGAGAAGTATTGAAGCTTCTCTTTGATGAAGGAGAGGGTGTTGGTTATGGAGATGCTCTTTTGGCAGTGTTGCCATCATTTCATGGAATCAAGTAGGGTTGTCTCCTATATTTCTGCTGTAGTTTAGTTGAGGCAACCTTGTTGGGAGAGATCAAGGTTTGCATTTTGGCATTTTTTTTTAATTCTTTTCATCTTTTTGTTTGTCTCGTTGATCAAGGTTTGCATTTTGGCATTTTTTTTTAATTCTTTTCATCTTTTTGTTTGTCTCGTTGCATTGTGCAATTGAGGGACATAAGTGTAGCCGGTGAATGGATGTAATTGGTATTATACATTTGACAATAGTTTCAAGTAATAATTTTGTTCTTCAATTTTTAATTTGTATTAAGTAACAATGCATGCAGTTTTCTGTTTCT |
| 03 | CL4684.Contig1_All | AAAATAACACAATAGAAAACAATTGTGGTACTCGTCTTCTAGCTTATTCTATTCATTTCCAAGTGCATCTCTTTTGGTGCTAAACTGCAATGATGTTAAAAATAGCAACTTGCAAATTCAGAAGCAGGTAGTCGCATCTGAACACAGTTGCATACTACCTTTCTGGTGCATTAATCTCACCTCGTTATCCTTTTCAGCTTCTCTCTGTAACTCCATTCCATTACAACGTCTTTATATTTTTTAGAGTTTCAGATTCGATTGGTTTTAATCGCTCTGAAGTTTGAACCCTACTGGGATTCTCTGATTTGCCAAGACTGAAAGGTTTCAATGGGTTCTTGTAGCTTAAGAACTTCAAATTTAAAAATTTTGAGCTTGGACTTCGGTAGAATAAAAGTTGGTGGTTTGAAACCATGGTATGACATGAAAACTTGGAAAGCACGGACACCATTACATTGTAAAGGTTTGGTGATATCGCGGAGTGCAGAGAAAGCATCAACTGTATGTTGTGATCCATCTTCAGAAACTGACTCTGCTACAAATATAGAGCATGGTTCTGTAGAGACAAAAGTTTCTGGGTTGACAAGCAAGCGCATTCCGAATTCATATGAGGTGGAATATCTGCTGAGAGAAATATGTGATACAACTTCAATTGCAGAGTTCGAAATGAAGCTAAATGGGTTTCGGTTATACATGACAAGGGACTTGACTGGAAAAGTTACAAGTCCACCTCCCCCTAGTTTTTCTCATGTTGGTGTAAATACAAGTATTGAGGCACCAGATGTAGACGGATCAGTATCTGAGCCAAATTTAGCCGTGTCCCAATCTCTACCTTCTTCAGGAAGTATTCGGAGATTGCTTGATAATGCTGCAGATGAAGGCTTAATGATAATCCAGTCTCCTAGAGTTGGGTATTTTAGGCGGTCACGAACCATAAAGGGGAAGCGTGCTCCTCCATCATGTAAAGAGAAGCAAACAGTGAAGGAGGGCCAAGTACTTTGCTATATCGAACAGCTTGGTGGCGAGATCCCTGTCGAGTCTGATGTCTCAGGAGAGGTCATAAAGTTGCTACGGGAGGATGGTGAACCTGTCGGATATGGTGATGCTCTTGTTGCAATTCTCCCATCTTTTCCTGGGATAAAGAAGCTTTAGTGGATGCTTTTGGTTTCCATTTCATTTGTTTTTTTTTTTAAACATGTCAATTTTCCAAGTTTATTTGAGACCCAGATGATTTTTTTTATTTGGATTGAGGCCCCTAATTTATGTATCAGAGTGCATGATTGCTGAGAACTATTTGAGCATGGTGATTTTCTTATCTCAGTTTTGTATGAATGTGAAGATGAGTGGCAGAATTGCAGAAATGAATGATAGTGTTCTA |
| 04 | CL4684.Contig2_All | AAAATAACACAATAGAAAACAATTGTGGTACTCGTCTTCTAGCTTATTCTATTCATTTCCAAGTGCATCTCTTTTGGTGCTAAACTGCAATGATGTTAAAAATAGCAACTTGCAAATTCAGAAGCAGAGTTTCAGATTCGATTGGTTTTAATCGCTCTGAAGTTTGAACCCTACTGGGATTCTCTGATTTGCCAAGACTGAAAGGTTTCAATGGGTTCTTGTAGCTTAAGAACTTCAAATTTAAAAATTTTGAGCTTGGACTTCGGTAGAATAAAAGTTGGTGGTTTGAAACCATGGTATGACATGAAAACTTGGAAAGCACGGACACCATTACATTGTAAAGGTTTGGTGATATCGCGGAGTGCAGAGAAAGCATCAACTGTATGTTGTGATCCATCTTCAGAAACTGACTCTGCTACAAATATAGAGCATGGTTCTGTAGAGACAAAAGTTTCTGGGTTGACAAGCAAGCGCATTCCGAATTCATATGAGGTGGAATATCTGCTGAGAGAAATATGTGATACAACTTCAATTGCAGAGTTCGAAATGAAGCTAAATGGGTTTCGGTTATACATGACAAGGGACTTGACTGGAAAAGTTACAAGTCCACCTCCCCCTAGTTTTTCTCATGTTGGTGTAAATACAAGTATTGAGGCACCAGATGTAGACGGATCAGTATCTGAGCCAAATTTAGCCGTGTCCCAATCTCTACCTTCTTCAGGAAGTATTCGGAGATTGCTTGATAATGCTGCAGATGAAGGCTTAATGATAATCCAGTCTCCTAGAGTTGGGTATTTTAGGCGGTCACGAACCATAAAGGGGAAGCGTGCTCCTCCATCATGTAAAGAGAAGCAAACAGTGAAGGAGGGCCAAGTACTTTGCTATATCGAACAGCTTGGTGGCGAGATCCCTGTCGAGTCTGATGTCTCAGGAGAGGTCATAAAGTTGCTACGGGAGGATGGTGAACCTGTCGGATATGGTGATGCTCTTGTTGCAATTCTCCCATCTTTTCCTGGGATAAAGAAGCTTTAGTGGATGCTTTTGGTTTCCATTTCATTTGTTTTTTTTTTTAAACATGTCAATTTTCCAAGTTTATTTGAGACCCAGATGATTTTTTTTATTTGGATTGAGGCCCCTAATTTATGTATCAGAGTGCATGATTGCTGAGAACTATTTGAGCATGGTGATTTTCTTATCTCAGTTTTGTATGAATGTGAAGATGAGTGGCAGAATTGCAGAAATGAATGATAGTGTTCTA |
| 05 | CL4684.Contig3_All | CACTATCATTCATTTCTGCAATTCTGCCACTCATCTTCACATTCATACAAAACTGAGATAAGAAAATCACCATGCTCAAATAGTTCTCAGCAATCATGCACTCTGATACATAAATTAGGGGCCTCAATCCAAATAAAAAAATCATCTGGGTCTCAAATAAACTTGGAAAATTGACATGTTTAAAAAAAAAAACAAATGAAATGGAAACCAAAAGCATCCACTAAAGCTTCTTTATCCCAGGAAAAGATGGGAGAATTGCAACAAGAGCATCACCATATCCGACAGGTTCACCATCCTCCCGTAGCAACTTTATGACCTCTCCTGAGACATCAGACTCGACAGGGATCTCGCCACCAAGCTGTTCGATATAGCAAAGTACTTGGCCCTCCTTCACTGTTTGCTTCTCTTTACATGATGGAGGAGCACGCTTCCCCTTTATGGTTCGTGACCGCCTAAAATACCCAACTCTAGGAGACTGGATTATCATTAAGCCTTCATCTGCAGCATTATCAAGCAATCTCCGAATACTTCCTGAAGAAGGTAGAGATTGGGACACGGCTAAATTTGGCTCAGATACTGATCCGTCTACATCTGGTGCCTCAATACTTGTATTTACACCAACATGAGAAAAACTAGGGGGAGGTGGACTTGTAACTTTTCCAGTCAAGTCCCTTGTCATGTATAACCGAAACCCATTTAGCTTCATTTCGAACTCTGCAATTGAAGTTGTATCACATATTTCTCTCAGCAGATATTCCACCTCATATGAATTCGGAATGCGCTTGCTTGTCAACCCAGAAACTTTTGTCTCTACAGAACCATGCTCTATATTTGTAGCAGAGTCAGTTTCTGAAGATGGATCACAACATACAGTTGATGCTTTCTCTGCACTCCGCGATATCACCAAACCTTTACAATGTAATGGTGTCCGTGCTTTCCAAGTTTTCATGTCATACCATGGTTTCAAACCACCAACTTTTATTCTACCGAAGTCCAAGCTCAAAATTTTTAAATTTGAAGTTCTTAAGCTACAAGAACCCATTGAAACCTTTCAGTCTTGGCAAATCAGAGAATCCCAGTAGGGTTCAAACTTCAGAGCGATTAAAACCAATCGAATCTGAAACTCAGAAAGGTAGTATGCAACTGTGTTCAGATGCGACTACCTGCTTCTGAATTTGCAAGTTGCTATTTTTAACATCATTGCAGTTTAGCACCAAAAGAGATGCACTTGGAAATGAATAGAATAAGCTAGAAGACGAGTACCACAATTGTTTTCTATTGTGTTATTTT |
| 06 | CL6718.Contig1_All | ATGACAGCTAGTCAAAGCAAGTAAGGGCTTTTATCCATTCCATCCTTTCGGTTTCATCGATCAGGAACTGGAAGGCTGTTTTCTCTGTCCTCTTCTCCCTGCACTCACTTCCCCTGATGGATTCTTCCAGACAGTCTCCATAACACTCATAAAATTCATACATCACTTTTTCCGGTTCTCTCTATATGATATTTTTCTGCTGCTGCGTTTCTTTCTCTCTAGAACACAATGACCTCTTCTCTTTCGACGACGGCCGTTACCAAAGCTACCTCTTTACCTCTTTGCCAACCATGTGTTCGTTCTCTCTCTAAGGTCTCATTTCTTGTCACTTCCGAACCAAAGCTACGATTTCGTGCCAAGGATGGCATTGCCTGCATACTATGCGAAGTGGAATGGCTCAACCGCTAGACACTTTCTGGTCCAACACTTTTCATTAGACGGTTTGCAGTCTAGACGGAATCGTTTGACAGTGGTGAAAGCCCGATCAAATGAGAAGTACCGATGCCCAAAGCAAAGGATGCTAAGCCATCAAATGGGCCGCCTTCTGCAGCAGCTTCTGCTTCTGAGGAATACGTCTCTCAGTTTATTACTCAAGTTGCAAGTCTGGTCAAGCTTGTTGATTCCAGAGATATTGTAGAATTGGAATTGAAGCAGTTTGATTGTGAACTATTAATCCGTAAAAAGGAGGCTCTGCCCCAACCACCATCTCCTGCTCCACATATAATGATGCCTTCAGCTTCTCCACCAGCAGTAGCGTCACCATCTTTAGAACCATCTCCACCAGCTGCTTCTCCTGCACCTCCATCCCCAGCTCAAGGACCTGCTGCTGCTACCGCCGCCGCCGCCAAACCAGCCAAGTCATCATATCCACCTCTTAAATGCCCCATGGCAGGGATATTCTACCGATGTCCATCACCTGGTGAACCACCATTTGTTAAGGTTGGAGACAAAGTACAGAATGGACAGGTTTTATGCATCATTGAAGCCATGAAATTGATGAATGAAATAGAAGCTGATCAGTCGGGGACGATAGTTGAAGTCCTTGTAGATGATGGCAAGCCTGTCAGTGTAGATATGCCGCTGTTTGTGATTGAACCCTAGAGCTTGACGGTTGGATAGGCACATGTAATTTATTGGATGGTGAGTAGAAGGCAGGTGGTTTTTTCTTGTAATGAGATGTAGGTAGAAATTGTAACGAGACAGG |
| 07 | CL6718.Contig2_All | GGATGCGAATAGCAACGCGTCTTCACTACCCCCTAGACTGACAAATGACAGCTAGTCAAAGCAAGTAAGGGCTTTTATCCATTCCATCCTTTCGGTTTCATCGATCAGGAACTGGAAGGCTGTTTTCTCTGTCCTCTTCTCCCTGCACTCACTTCCCCTGATGGATTCTTCCAGACAGTCTCCATAACACTCATAAAATTCATACATCACTTTTTCCGGTTCTCTCTATATGATATTTTTCTGCTGCTGCGTTTCTTTCTCTCTAGAACACAATGACCTCTTCTCTTTCGACGACGGCCGTTACCAAAGCTACCTCTTTACCTCTTTGCCAACCATGTGTTCGTTCTCTCTCTAAGGTCTCATTTCTTGTCACTTCCGAACCAAAGCTACGATTTCGTGCCAAGGGTTTGCAGTCTAGACGGAATCGTTTGACAGTGGTGAAAGCCCGATCAAATGAGGTAAGGTTCTCTCAATGGTTTTCATTAGAGGATCTGGTTTCAAATTTTCGTTCCTTCATGGACATGCTTTTGGAGCCATTAACGGTTTCTCTGGATGGATCTACAAATGTTACTCCCCCAACAAAATCAGAAGTACCGATGCCCAAAGCAAAGGATGCTAAGCCATCAAATGGGCCGCCTTCTGCAGCAGCTTCTGCTTCTGAGGAATACGTCTCTCAGTTTATTACTCAAGTTGCAAGTCTGGTCAAGCTTGTTGATTCCAGAGATATTGTAGAATTGGAATTGAAGCAGTTTGATTGTGAACTATTAATCCGTAAAAAGGAGGCTCTGCCCCAACCACCATCTCCTGCTCCACATATAATGATGCCTTCAGCTTCTCCACCAGCAGTAGCGTCACCATCTTTAGAACCATCTCCACCAGCTGCTTCTCCTGCACCTCCATCCCCAGCTCAAGGACCTGCTGCTGCTACCGCCGCCGCCGCCAAACCAGCCAAGTCATCATATCCACCTCTTAAATGCCCCATGGCAGGGATATTCTACCGATGTCCATCACCTGGTGAACCACCATTTGTTAAGGTTGGAGACAAAGTACAGAATGGACAGGTTTTATGCATCATTGAAGCCATGAAATTGATGAATGAAATAGAAGCTGATCAGTCGGGGACGATAGTTGAAGTCCTTGTAGATGATGGCAAGCCTGTCAGTGTAGATATGGTGGGTTTCCTTCATCACCTTAGTTATCAAAAAATTGTTATCTACTTTCGTTTGTGTATGCTTACTTTAGGTTATTGCATTATGGTCTTGTTGCACAGCCGCTGTTTGTGATTGAACCCTAGAGCTTGACGGTTGGATAGGCACATGTAATTTATTGGATGGTGAGTAGAAGGCAGGTGGTTTTTTCTTGTAATGAGATGTAGGTAGAAATTGTAACGAGACAGGTG |
| 08 | CL6718.Contig3_All | ACTACCCCCTAGACTGACAAATGACAGCTAGTCAAAGCAAGTAAGGGCTTTTATCCATTCCATCCTTTCGGTTTCATCGATCAGGAACTGGAAGGCTGTTTTCTCTGTCCTCTTCTCCCTGCACTCACTTCCCCTGATGGATTCTTCCAGACAGTCTCCATAACACTCATAAAATTCATACATCACTTTTTCCGGTTCTCTCTATATGATATTTTTCTGCTGCTGCGTTTCTTTCTCTCTAGAACACAATGACCTCTTCTCTTTCGACGACGGCCGTTACCAAAGCTACCTCTTTACCTCTTTGCCAACCATGTGTTCGTTCTCTCTCTAAGGTCTCATTTCTTGTCACTTCCGAACCAAAGCTACGATTTCGTGCCAAGGGTTTGCAGTCTAGACGGAATCGTTTGACAGTGGTGAAAGCCCGATCAAATGAGAAGTACCGATGCCCAAAGCAAAGGATGCTAAGCCATCAAATGGGCCGCCTTCTGCAGCAGCTTCTGCTTCTGAGGAATACGTCTCTCAGTTTATTACTCAAGTTGCAAGTCTGGTCAAGCTTGTTGATTCCAGAGATATTGTAGAATTGGAATTGAAGCAGTTTGATTGTGAACTATTAATCCGTAAAAAGGAGGCTCTGCCCCAACCACCATCTCCTGCTCCACATATAATGATGCCTTCAGCTTCTCCACCAGCAGTAGCGTCACCATCTTTAGAACCATCTCCACCAGCTGCTTCTCCTGCACCTCCATCCCCAGCTCAAGGACCTGCTGCTGCTACCGCCGCCGCCGCCAAACCAGCCAAGTCATCATATCCACCTCTTAAATGCCCCATGGCAGGGATATTCTACCGATGTCCATCACCTGGTGAACCACCATTTGTTAAGGTTGGAGACAAAGTACAGAATGGACAGGTTTTATGCATCATTGAAGCCATGAAATTGATGAATGAAATAGAAGCTGATCAGTCGGGGACGATAGTTGAAGTCCTTGTAGATGATGGCAAGCCTGTCAGTGTAGATATGCCGCTGTTTGTGATTGAACCCTAGAGCTTGACGGTTGGATAGGCACATGTAATTTATTGGATGGTGAGTAGAAGGCAGGTGGTTTTTTCTTGTAATGAGATGTAGGTAGAAATTGTAACGAGACAGGTG |
| 09 | CL6718.Contig4_All | GGATGCGAATAGCAACGCGTCTTCACTACCCCCTACACTGACAAATGACAGCTAGTCAAAGCAAGTAAGGGCTTTTATCCATTCCATCCTTTCGGTTTCATCGATCAGGAACTGGAAGGCTGTTTTCTCTGTCCTCTTCTCCCTGCACTCACTTCCCCTGATGGATTCTTCCAGACAGTCTCCATAACACTCATAAAATTCATACATCACTTTTTCCGGTTCTCTCTATATGATATTTTTCTGCTGCTGCGTTTCTTTCTCTCTAGAACACAATGACCTCTTCTCTTTCGACGACGGCCGTTACCAAAGCTACCTCTTTACCTCTTTGCCAACCATGTGTTCGTTCTCTCTCTAAGGTCTCATTTCTTGTCACTTCCGAACCAAAGCTACGATTTCGTGCCAAGGATGGCATTGCCTGCATACTATGCGAAGTGGAATGGCTCAACCGCTAGACACTTTCTGGTCCAACACTTTTCATTAGACGGTTTGCAGTCTAGACGGAATCGTTTGACAGTGGTGAAAGCCCGATCAAATGAGGTAAGGTTCTCTCAATGGTTTTCATTAGAGGATCTGGTTTCAAATTTTCGTTCCTTCATGGACATGCTTTTGGAGCCATTAACGGTTTCTCTGGATGGATCTACAAATGTTACTCCCCCAACAAAATCAGAAGTACCGATGCCCAAAGCAAAGGATGCTAAGCCATCAAATGGGCCGCCTTCTGCAGCAGCTTCTGCTTCTGAGGAATACGTCTCTCAGTTTATTACTCAAGTTGCAAGTCTGGTCAAGCTTGTTGATTCCAGAGATATTGTAGAATTGGAATTGAAGCAGTTTGATTGTGAACTATTAATCCGTAAAAAGGAGGCTCTGCCCCAACCACCATCTCCTGCTCCACATATAATGATGCCTTCAGCTTCTCCACCAGCAGTAGCGTCACCATCTTTAGAACCATCTCCACCAGCTGCTTCTCCTGCACCTCCATCCCCAGCTCAAGGACCTGCTGCTGCTACCGCCGCCGCCGCCAAACCAGCCAAGTCATCATATCCACCTCTTAAATGCCCCATGGCAGGGATATTCTACCGATGTCCATCACCTGGTGAACCACCATTTGTTAAGGTTGGAGACAAAGTACAGAATGGACAGGTTTTATGCATCATTGAAGCCATGAAATTGATGAATGAAATAGAAGCTGATCAGTCGGGGACGATAGTTGAAGTCCTTGTAGATGATGGCAAGCCTGTCAGTGTAGATATGCCGCTGTTTGTGATTGAACCCTAGAGCTTGACGGTTGGATAGGCACATGTAATTTATTGGATGGTGAGTAGAAGGCAGGTGGTTTTTTCTTGTAATGAGATGTAGGTAGAAATTGTAACGAGACAGG |
| 10 | CL6718.Contig5_All | CGCGTCTTCACTACCCCCTAGACTGACAAATGACAGCTAGTCAAAGCAAGTAAGGGCTTTTATCCATTCCATCCTTTCGGTTTCATCGATCAGGAACTGGAAGGCTGTTTTCTCTGTCCTCTTCTCCCTGCACTCACTTCCCCTGATGGATTCTTCCAGACAGTCTCCATAACACTCATAAAATTCATACATCACTTTTTCCGGTTCTCTCTATATGATATTTTTCTGCTGCTGCGTTTCTTTCTCTCTAGAACACAATGACATCTTCTCTTTCGACGACGGCCGTTACCAAAGCTACCTCTTTACCTCTTTGCCAACCATGTGTTCGTTCTCTCTCTAAGGTCTCATTTCTTGTCACTTCCGAACCAAAGCTACGATTTCGTGCCAAGGATGGCATTGCCTGCATACTATGCGAAGTGGAATGGCTCAACCGCTAGACACTTTCTGGTCCAACACTTTTCATTAGACGTAAACTTCTAATCCAATTTTTTTTACTAGATAAGTATATTTACTGTTTCGTTCTTTCTAATTCGGTTGTCATTTAGAAGTTAATTTATTAGACATTTTAGTTGTCCAAGAAGGTTATGTTTTTCAATGGAAGAATTTTTGGATAAGTTAGTCTTGTGGTAAGTACCCCGAATTTTCACCCTTCCAACTCAGGTTTGAATTCTGTTTTCCACCAAATAATTCTCCCTCGGGTAGCAACCGAGCTACAACCTAGTGGAAGATACATACCCATAGACACAAATTTAGAAACTAATACCATTAACCATCTCTTCTTTCCAATTTAACAGAAGACACTAGAATTCTTGTACTGATATGTAGTAGTTTAATGTCCACATTGTAGGGTTTGCAGTCTAGACGGAATCGTTTGACAGTGGTGAAAGCCCGATCAAATGAGGTAAGGTTCTCTCAATGGTTTTCATTAGAGGATCTGGTTTCAAATTTTCGTTCCTTCATGGACATGCTTTTGGAGCCATTAACGGTTTCTCTGGATGGATCTACAAATGTTACTCCCCCAACAAAATCAGAAGTACCGATGCCCAAAGCAAAGGATGCTAAGCCATCAAATGGGCCGCCTTCTGCAGCAGCTTCTGCTTCTGAGGAATACGTCTCTCAGTTTATTACTCAAGTTGCAAGTCTGGTCAAGCTTGTTGATTCCAGAGATATTGTAGAATTGGAATTGAAGCAGTTTGATTGTGAACTATTAATCCGTAAAAAGGAGGCTCTGCCCCAACCACCATCTCCTGCTCCACATATAATGATGCCTTCAGCTTCTCCACCAGCAGTAGCGTCACCATCTTTAGAACCATCTCCACCAGCTGCTTCTCCTGCACCTCCATCCCCAGCTCAAGGACCTGCTGCTGCTACCGCCGCCGCCGCCAAACCAGCCAAGTCATCATATCCACCTCTTAAATGCCCCATGGCAGGGATATTCTACCGATGTCCATCACCTGGTGAACCACCATTTGTTAAGGTTGGAGACAAAGTACAGAATGGACAGGTTTTATGCATCATTGAAGCCATGAAATTGATGAATGAAATAGAAGTAAGACATTCTTGCTTTAGTATTTTTTTTTTCAATTAATCACATGTATATATATACATGTTTTTTTCTTTGTCTATAGGCTGATCAGTCGGGGACGATAGTTGAAGTCCTTGTAGATGATGGCAAGCCTGTCAGTGTAGATATGGTGGGTTTCCTTCATCACCTTAGTTATCAAAAAATTGTTATCTACTTTCGTTTGTGTATGCTTACTTTAGGTTATTGCATTATGGTCTTGTTGCACAGCCGCTGTTTGTGATTGAACCCTAGAGCTTGACGGTTGGATAGGCACATGTAATTTATTGGATGGTGAGTAGAAGGCAGGTGGTTTTTTCTTGTAATGAGATGTAGGTAGAAATTGTAACGAGACAGGTG |
| 11 | CL6718.Contig6_All | GGATGCGAATAGCAACGCGTCTTCACTACCCCCTACACTGACAAATGACAGCTAGTCAAAGCAAGTAAGGGCTTTTATCCATTCCATCCTTTCGGTTTCATCGATCAGGAACTGGAAGGCTGTTTTCTCTGTCCTCTTCTCCCTGCACTCACTTCCCCTGATGGATTCTTCCAGACAGTCTCCATAACACTCATAAAATTCATACATCACTTTTTCCGGTTCTCTCTATATGATATTTTTCTGCTGCTGCGTTTCTTTCTCTCTAGAACACAATGACCTCTTCTCTTTCGACGACGGCCGTTACCAAAGCTACCTCTTTACCTCTTTGCCAACCATGTGTTCGTTCTCTCTCTAAGGTCTCATTTCTTGTCACTTCCGAACCAAAGCTACGATTTCGTGCCAAGGGTTTGCAGTCTAGACGGAATCGTTTGACAGTGGTGAAAGCCCGATCAAATGAGGTAAGGTTCTCTCAATGGTTTTCATTAGAGGATCTGGTTTCAAATTTTCGTTCCTTCATGGACATGCTTTTGGAGCCATTAACGGTTTCTCTGGATGGATCTACAAATGTTACTCCCCCAACAAAATCAGAAGTACCGATGCCCAAAGCAAAGGATGCTAAGCCATCAAATGGGCCGCCTTCTGCAGCAGCTTCTGCTTCTGAGGAATACGTCTCTCAGTTTATTACTCAAGTTGCAAGTCTGGTCAAGCTTGTTGATTCCAGAGATATTGTAGAATTGGAATTGAAGCAGTTTGATTGTGAACTATTAATCCGTAAAAAGGAGGCTCTGCCCCAACCACCATCTCCTGCTCCACATATAATGATGCCTTCAGCTTCTCCACCAGCAGTAGCGTCACCATCTTTAGAACCATCTCCACCAGCTGCTTCTCCTGCACCTCCATCCCCAGCTCAAGGACCTGCTGCTGCTACCGCCGCCGCCGCCAAACCAGCCAAGTCATCATATCCACCTCTTAAATGCCCCATGGCAGGGATATTCTACCGATGTCCATCACCTGGTGAACCACCATTTGTTAAGGTTGGAGACAAAGTACAGAATGGACAGGTTTTATGCATCATTGAAGCCATGAAATTGATGAATGAAATAGAAGCTGATCAGTCGGGGACGATAGTTGAAGTCCTTGTAGATGATGGCAAGCCTGTCAGTGTAGATATGCCGCTGTTTGTGATTGAACCCTAGAGCTTGACGGTTGGATAGGCACATGTAATTTATTGGATGGTGAGTAGAAGGCAGGTGGTTTTTTCTTGTAATGAGATGTAGGTAGAAATTGTAACGAGACAGGTG |
| 12 | CL750.Contig1_All | CAAATGTAATGGAATCTCTCCCCTCATTTCTCTCTCTACAATTTATTTGCACCACCTCGTAAATATCGATAGTATCTGTCGACCTACATATGACCTCAGTTGTATTCGTCTTTTCATTTCGTTGGATTCCTCACGAGTCTCTCCGATTCCTTCGCCCAATTTTTCTTTCAAATCTCTCATTCTCTCTCTTCTTTCCCAGCTCGAGTTAGGGTTTTTAGCTGCTTTTGCTGATTCAATTTCAATTCATGGCGTCTTTCACCGTTTCTTGCCCTAAATCCTCTTTGGTCGGAGCGTCGTGTCCGAACCCGAATCAGTATCAACAACCGCACCATCATTCGATATCGTTTCCGTTAATTTCAAATGCCAAGTCTGGATTCATGGTTGGGTCGTTGCAAGATCGTCTAAGTTCTTTTGGATTACAGGGGTCTGGCCGGATCCAATCCGATGCCCTTAAGGTACATGCACAACTTAATAAGTTTGACTATATCCCCAGCCAGCCCCCTTTACCTTTTTCATATTTTCCTATTGTAATTCTAAATTTTACTTCTTCCATTTTGCTTCACAAGTATAAGGCTGCTACCGATAAATCATCAAATTATGCACCAGTTCCTGAGGAGAACAAATCATCAAATTCTGCACCAGTTCCCGAGGAGAACAAATCATCAAATTCTGCACCAGTTCCCGAGGAGAACAAATCATCAAATTCTGCACCAGTTCCTGAGAAAGAAATTGCCAAAAAACCTGCCCACAGAAACTCTGTTCCAGATGCAGCATCCATTTCAGCATTTATGACTCAAGTGGCAGACCTTGTTAAGTTAGTAGATTCGAGGGACATTATGGAGCTAGAGCTAAAGCAACTGGACTGTGAGCTACTTATAAGGAAAAAGGAAGCTCTGCCACAGCCACCTGCTGCTCCTGTTATGATGCAATCCCCTCCTCCTCAATTTCAGATGCCTTCACCTCATCAGGACATTCCCTCTCCTCCCTCCCCACCTCCAGTTTCAGCACCCGTGCCACCTACCCCTGCAAAGCCAACCAAGTCCTCTTCTTCTCATCCACCCTTGAAGTGCCCTATGGCAGGAACATTTTATCGTTGTCCTGCACCTGGCACACCGCCTTTTGTAAAGGTAGGAGACAAGATTCAGAAAGGCCAGGTAGTTTGCATCATTGAAGCTATGAAGCTGATGAATGAGATTGAATCTGATCAGTCGGGAACCGTAGTTGAGATACTTGTAGATGACGGGAAACCAGTTAGTGTGGATACGCCTCTGTTTGCCATTGAGCCCTGAAAAGCCATTGTTTATTCACAAGTATCTTCTATTTGTTGATGAGCCATGAAGATTAAGCATCTGCTGATTTTGACTAGCTTGGCTGGTCTATGGAAGTCTCGAAATAATTCAATTATACATATGAGACGTGGGTTTTTCTCCACCATCTCCCAATGTTCTATGGATTTTTTTTTTTCAGTTTTTCAAATGGCATTATACAAAATGAATTTTCTTTTCGGATTTTCTGAAATTCCAAAAGAAATTTATGTTTAATTTATTAATTTAGATAAATATGAGTCGG |
| 13 | CL750.Contig2_All | TGCCATTTGAAAAACTGAAAAAAAAAATCCATAGAACATTGGGAGATGGTGGAGAAAAACCCACGTCTCATATGTATAATTGAATTATTTCGAGACTTCCATAGACCAGCCAAGCTAGTCAAAATCAGCAGATGCTTAATCTTCATGGCTCATCAACAAATAGAAGATACTTGTGAATAAACAATGGCTTTTCAGGGCTCAATGGCAAACAGAGGCGTATCCACACTAACTGGTTTCCCGTCATCTACAAGTATCTCAACTACGGTTCCCGACTGATCAGATTCAATCTCATTCATCAGCTTCATAGCTTCAATGATGCAAACTACCTGGCCTTTCTGAATCTTGTCTCCTACCTTTACAAAAGGCGGTGTGCCAGGTGCAGGACAACGATAAAATGTTCCTGCCATAGGGCACTTCAAGGGTGGATGAGAGGAAGAGGACTTGGTTGGCTTTGCAGGGGTAGGTGGCACGGGTGCTGAAACTGGAGGTGGGGAGGGAGGAGAGGGAATGTCCTGATGAGGTGAAGGCATCTGAAATTGAGGAGGAGGGGATTGCATCATAACAGGAGCAGCAGGTGGCTGTGGCAGAGCTTCCTTTTTCCTTATAAGTAGCTCACAGTCCAGTTGCTTTAGCTCTAGCTCCATAATGTCCCTCGAATCTACTAACTTAACAAGGTCTGCCACTTGAGTCATAAATGCTGAAATGGATGCTGCATCTGGAACAGAGTTTCTGTGGGCAGGTTTTTCGGCAATTTCTTTCTCAGGAACTGGTGCAGAATTTGATGATTTGTTCTCCTCGGGAACTGGTGCAGAATTTGATGATTTGTTCTCCTCGGGAACTGGTGCAGAATTTGATGATTTGTTCTCCTCAGGAACTGGTGCATAATTTGATGATTTATCGGTAGCAGCCTTATACTTAAGGGCATCGGATTGGATCCGGCCAGACCCCTGTAATCCAAAAGAACTTAGACGATCTTGCAACGACCCAACCATGAATCCAGACTTGGCATTTGAAATTAACGGAAACGATATCGAATGATGGTGCGGTTGTTGATACTGATTCGGGTTCGGACACGACGCTCCGACCAAAGAGGATTTAGGGCAAGAAACGGTGAAAGACGCCATGAATTGAAATTGAATCAGCAAAAGCAGCTAAAAACCCTAACTCGAGCTGGGAAAGAAGAGAGAGAATGAGAGATTTGAAAGAAAAATTGGGCGAAGGAATCGGAGAGACTCGTGAGGAATCCAACGAAATGAAAAGACGAATACAACTGAGGTCATATGTAGGTCGACAGATACTATCGATATTTACGAGGTGGTGCAAATAAATTGTAGAGAGAGAAATGAGGGGAGAGATTCCATTACATTTGTT |
| 14 | CL750.Contig4_All | TAATAAATTAAACATAAATTTCTTTTGGAATTTCAGAAAATCCGAAAAGAAAATTCATTTTGTATAATGCCATTTGAAAAACTGAAAAAAAAAAATCCATAGAACATTGGGAGATGGTGGAGAAAAACCCACGTCTCATATGTATAATTGAATTATTTCGAGACTTCCATAGACCAGCCAAGCTAGTCAAAATCAGCAGATGCTTAATCTTCATGGCTCATCAACAAATAGAAGATACTTGTGAATAAACAATGGCTTTTCAGGGCTCAATGGCAAACAGAGGCGTATCCACACTAACTGGTTTCCCGTCATCTACAAGTATCTCAACTACGGTTCCCGACTGATCAGATTCAATCTCATTCATCAGCTTCATAGCTTCAATGATGCAAACTACCTGGCCTTTCTGAATCTTGTCTCCTACCTTTACAAAAGGCGGTGTGCCAGGTGCAGGACAACGATAAAATGTTCCTGCCATAGGGCACTTCAAGGGTGGATGAGAGGAAGAGGACTTGGTTGGCTTTGCAGGGGTAGGTGGCACGGGTGCTGAAACTGGAGGTGGGGAGGGAGGAGAGGGAATGTCCTGATGAGGTGAAGGCATCTGAAATTGAGGAGGAGGGGATTGCATCATAACAGGAGCAGCAGGTGGCTGTGGCAGAGCTTCCTTTTTCCTTATAAGTAGCTCACAGCCCAGTTGCTTTAGCTCTAGCTCCATAATGTCCCTCGAATCTACTAACTTAACAAGGTCTGCCACTTGAGTCATAAATGCTGAAATGGATGCTGCATCTGGAACAGAGTTTCTGTGGGCAGGTTTTTCGGCAATTTCTTTCTCAGGAACTGGTGCAGAATTTGATGATTTGTTCTCCTCGGGAACTGGTGCAGAATTTGATGATTTGTTCTCCTCGGGAACTGGTGCAGAATTTGATGATTTGTTCTCCTCAGGAACTGGTGCATAATTTGATGATTTATCGGTAGCAGCCTTATACTGTAATCCAAAAGAACTTAGACGATCTTGCAACGACCCAACCATGAATCCAGACTTGGCATTTGAAATTAACGGAAACGATATCGAATGATGGTGCGGTTGTTGATACTGATTCGGGTTCGGACACGACGCTCCGACCAAAGAGGATTTAGGGCAAGAAACGGTGAAAGACGCCATGAATTGAAATTGAATCAGCAAAAGCAGCTAAAAACCCTAACTCGAGCTGGGAAAGAAGAGAGAGAATGAGAGATTTGAAAGAAAAATTGGGCGAAGGAATCGGAGAGACTCGTGAGGAATCCAACGAAATGAAAGTTTGTTGTCCTTTCTTTCTTCTCATCCCAATTTGTACACTATTCCTTGTCTGTTCCTGACTTCTTTCTCTTTTGATTCTTGGTTTCTTTCCTGTTTTCTTGAGTTTGGGATGTTGTTGGTTTGTTTTGATTATGGCTATGAAAGGAAAATTTATATTCTGTAGATTCAAGCCTTTTGAGTTCATCGACTGGAATTCAGATCTTAATAAGTTTATCGAAAGGGGCAAAGGCTACACTAAATTTGTTCTGTTAAAAGCACACGAAAATCGTTGGGTGTTTTACTTTCTTACGGGGTTCCTGGCAAGCTATTCAGGATTGTGGTTGCCTATATGCAGATTCCGGCAACTCTAACTCCGGTGGAGGAGAAAAGGGTAATGGACTTAGTGGCGATACTTCCAAGGTTGGGGAAGGTGAAACAAAGCGGATGGACCGGACTAATCAATCTCCAGTTTGCTATGGTTGTAGTAGGAAACATGTTTTGACCAGGGGCATAGGAAGATGGAGATTAGCATGAGTGACAGCTTCGCTGAGGGCATTGACCTCAAACATGTTTCCTACAACCACCATAGCAAACTGGAGTCATTCTCACGTCGTCATTCAGCAGAGTCATTAGGTCTGGTCTGGGAATGAGTGTTCTTGTAAAGATTAGGTCTGGTCTGGGAATGACGCAGATTTGTCGGCTTCATCTCCATCTGGAAGTCTCTGTCAGTGATCTTCATCGCCGTCCAACAGGAACATTGTTGCAACAGTCAACTTAGATTGCAAGTTGGATCTTAAGGAAATTGCTTTGCATGCTCGTAATGCAGAATACAATCCCAAGCGTTTCGCTGCTGTTATTATGAGGACACGGGATCCAAAAACTACGGCTTTGATTTTTGCCTCTGGAAAGATGTTGCTTCATGTGATGTTAAATTCCCTACCAGACTCGAAGGTCTCGCGTATGGACATGGCCAGTTTTGTAGTTATGTACCAGAAATTTTTCCGGGTTTAATATATCGCATGAAACAACCGTAGATTGTGCTTCTTATCTTTGTATCAGGAAAAATTGTTCTTACTGGAGCCAAGGTAAAAGAAGACACTTTATAGGGCTTTTGAGAACATATATCGTGCCCTTACTGGGTTTAGAAAGATTCAACAATGAATAATGGGCAATTTTGTTTAAAATTATTGTAACAAGTTGTTTTGAGAACGGATGATATGCGTCACCATATAATTTCTATATCAATGTTTCGTTATTGAGGGTGTGGTGATAATTTTCATCCCTCCTCGTTACTTTGAGGTAGGAAGAGGTTGTCATTTCACAAACAAAGTCTATTTTCCTTGCTATTAGATAAT |
| 15 | CL750.Contig5_All | AAACAAATGTAATGGAATCTCTCCCCTCATTTCTCTCTCTACAATTTATTTGCACCACCTCGTAAATATCGATAGTATCTGTCGACCTACATATGACCTCAGTTGTATTCGTCTTTTCATTTCGTTGGATTCCTCACGAGTCTCTCCGATTCCTTCGCCCAATTTTTCTTTCAAATCTCTCATTCTCTCTCTTCTTTCCCAGCTCGAGTTAGGGTTTTTAGCTGCTTTTGCTGATTCAATTTCAATTCATGGCGTCTTTCACCGTTTCTTGCCCTAAATCCTCTTTGGTCGGAGCGTCGTGTCCGAACCCGAATCAGTATCAACAACCGCACCATCATTCGATATCGTTTCCGTTAATTTCAAATGCCAAGTCTGGATTCATGGTTGGGTCGTTGCAAGATCGTCTAAGTTCTTTTGGATTACAGTATAAGGCTGCTACCGATAAATCATCAAATTATGCACCAGTTCCTGAGGAGAACAAATCATCAAATTCTGCACCAGTTCCCGAGGAGAACAAATCATCAAATTCTGCACCAGTTCCTGAGAAAGAAATTGCCGAAAAACCTGCCCACAGAAACTCTGTTCCAGATGCAGCATCCATTTCAGCATTTATGACTCAAGTGGCAGACCTTGTTAAGTTAGTAGATTCGAGGGACATTATGGAGCTAGAGCTAAAGCAACTGGGCTGTGAGCTACTTATAAGGAAAAAGGAAGCTCTGCCACAGCCACCTGCTGCTCCTGTTATGATGCAATCCCCTCCTCCTCAATTTCAGATGCCTTCACCTCATCAGGACATTCCCTCTCCTCCCTCCCCACCTCCAGTTTCAGCACCCGTGCCACCTACCCCTGCAAAGCCAACCAAGTCCTCTTCCTCTCATCCACCCTTGAAGTGCCCTATGGCAGGAACATTTTATCGTTGTCCTGCACCTGGCACACCGCCTTTTGTAAAGGTAGGAGACAAGATTCAGAAAGGCCAGGTAGTTTGCATCATTGAAGCTATGAAGCTGATGAATGAGATTGAATCTGATCAGTCGGGAACCGTAGTTGAGATACTTGTAGATGACGGGAAACCAGTTAGTGTGGATACGCCTCTGTTTGCCATTGAGCCCTGAAAAGCCATTGTTTATTCACAAGTATCTTCTATTTGTTGATGAGCCATGAAGATTAAGCATCTGCTGATTTTGACTAGCTTGGCTGGTCTATGGAAGTCTCGAAATAATTCAATTATACATATGAGACGTGGGTTTTTCTCCACCATCTCCCAATGTTCTATGGATTTTTTTTTTTCAGTTTTTCAAATGGCATTATACAAAATGAATTTTCTTTTCGGATTTTCTGAAATTCCAAAAGAAATTTATGTTTAATTTATTAATTTAGATAAATATGAG |
| 16 | CL750.Contig6_All | AAACAAATGTAATGGAATCTCTCCCCTCATTTCTCTCTCTACAATTTATTTGCACCACCTCGTAAATATCGATAGTATCTGTCGACCTACATATGACCTCAGTTGTATTCGTCTTTTCATTTCGTTGGATTCCTCACGAGTCTCTCCGATTCCTTCGCCCAATTTTTCTTTCAAATCTCTCATTCTCTCTCTTCTTTCCCAGCTCGAGTTAGGGTTTTTAGCTGCTTTTGCTGATTCAATTTCAATTCATGGCGTCTTTCACCGTTTCTTGCCCTAAATCCTCTTTGGTCGGAGCGTCGTGTCCGAACCCGAATCAGTATCAACAACCGCACCATCATTCGATATCGTTTCCGTTAATTTCAAATGCCAAGTCTGGATTCATGGTTGGGTCGTTGCAAGATCGTCTAAGTTCTTTTGGATTACAGGGGTCTGGCCGGATCCAATCCGATGCCCTTAAGTATAAGGCTGCTACCGATAAATCATCAAATTATGCACCAGTTCCTGAGGAGAACAAATCATCAAATTCTGCACCAGTTCCCGAGGAGAACAAATCATCAAATTCTGCACCAGTTCCTGAGAAAGAAATTGCCGAAAAACCTGCCCACAGAAACTCTGTTCCAGATGCAGCATCCATTTCAGCATTTATGACTCAAGTGGCAGACCTTGTTAAGTTAGTAGATTCGAGGGACATTATGGAGCTAGAGCTAAAGCAACTGGGCTGTGAGCTACTTATAAGGAAAAAGGAAGCTCTGCCACAGCCACCTGCTGCTCCTGTTATGATGCAATCCCCTCCTCCTCAATTTCAGATGCCTTCACCTCATCAGGACATTCCCTCTCCTCCCTCCCCACCTCCAGTTTCAGCACCCGTGCCACCTACCCCTGCAAAGCCAACCAAGTCCTCTTCCTCTCATCCACCCTTGAAGTGCCCTATGGCAGGAACATTTTATCGTTGTCCTGCACCTGGCACACCGCCTTTTGTAAAGGTAGGAGACAAGATTCAGAAAGGCCAGGTAGTTTGCATCATTGAAGCTATGAAGCTGATGAATGAGATTGAATCTGATCAGTCGGGAACCGTAGTTGAGATACTTGTAGATGACGGGAAACCAGTTAGTGTGGATACGCCTCTGTTTGCCATTGAGCCCTGAAAAGCCATTGTTTATTCACAAGTATCTTCTATTTGTTGATGAGCCATGAAGATTAAGCATCTGCTGATTTTGACTAGCTTGGCTGGTCTATGGAAGTCTCGAAATAATTCAATTATACATATGAGACGTGGGTTTTTCTCCACCATCTCCCAATGTTCTATGGATTTTTTTTTTTCAGTTTTTCAAATGGCATTATACAAAATGAATTTTCTTTTCGGATTTTCTGAAATTCCAAAAGAAATTTATGTTTAATTTATTAATTTAGATAAATATGAG |
| 17 | Unigene23603_All | TAGGACAACTAGGAAACACCAAAATAACTTCATTACTTCCGCTTCAAACTGTCTTTCCTTCTATGAACTCAAGAAAGTCGTTGACCTTTTTCTAAAAGTAAAAACCCTAATTATAAAAGGTAGAAAAGAGAGGCTTGGCCAGGCAAAAACAACATTATTACAGGCAACATAAATTAAATTCTCAGTAAAATGGTGACTGCCTCTAAAAGATTGAGATAGGTCCTTGCTAAGTTCTTCACGGTGCAATGACAAGAAGAGGTGTATCTACGCTAACTGGTTTTCCGTCCTCTGCCAATATCTCAGTTATTGTTCCAGTCTGATCAGCTTCAATTTCATTCATCAATTTCATGGCCTCAATGATGCAAATGACTTGTCCTTTCTGCACTTTATCTCCCACCTTGACAAATGGTGGTTCACCAGGTGCAGGACAGCGATAGAAAGTTCCAGCCATAGGACATTTGAATGGTGGATGGGATGATTTACTTGTCTTTGCAGGGGCAGGTAAGGCAGGTGGTACAGCTGAAGGTGCAGGATTTGGAGCAGCAGTCGGAGCTGGAGGTGGTTGAGATTGATACATGGATTGGGGCATTGGAGGTTGCATGGTGTACATCGGGGCAGCTTGTTGTGGTGGCTGCATCGCTTCCTTTTTTCTTATTAGAAGCTCACAGTCCAATTGTTTCAGCTGCAGCTCCATTATATCTCTTGAATCCACCAGTTTAACTAAGTCTGTCACTTGACTCATGAATGCTGAGATTGATGATTCATCTGTGATAGTGCTGGGAACTGAAAGCTTTTCTGCAGACTCATCTTTTACCAATTTGGTCTCAACTACGGGTGCAGAATTGGAAGTTTGTTTGGAAAAAACCTTCCAGACAGCGTTCTGCTTCCTCATGGGACACTTAATCCCAAAAGCCGCAAAATCGTCAAACGAAGATCCAGATTTCAGCATAGCCCTCGAATTTGAGCCATGAGGAAACGAGATCTTGGACTGGTGATTTGGAAATTGAGGTGTTGATGCAGAACGACAAATCAAAGTGGTCTTAGGGCATGGAACAGAGATTGAGGCCATTTTTTCTGAACCAACCCAATTAGACAATGTAGCAACTCAAATCAAGAGAATTGATGATAGCTGAGCGAACTCAGATCTGTAGAAAAAACGAAAACAGAAAGCAATTTGAATGAGCTGTTTATATACGTTTCTTTATTTGTATTTTGAAAGCTTTTGAAAAGGGGATGAAGGGTGGGGGCGATTATGACGAAGAGGCGATCTGAGAAATTTGAGGGTAATATTTTTTTAATTTGAGAAAACAACAAACATGTTTTTTGGCTTCGATGCTTCCAAGGAGACCGTCAACATCGCCTCACGCTACAAATGCATGCACTCGGAGAT |
