# Supplementary material for: Identification of genes associated with the biosynthesis of unsaturated fatty acid and oil accumulation in herbaceous peony ‘Hangshao’ (Paeonia lactiflora ‘Hangshao’) seeds based on transcriptome analysis
Source: BMC Genomics. 2021 Feb 1;22:94. doi: 10.1186/s12864-020-07339-7 (PMC7849092; doi:10.1186/s12864-020-07339-7)
Supplement: Supplementary file 15 — Additional file 15: Table S12. Gene-specific primer sequence for qRT-PCR detection [file 12864_2020_7339_MOESM15_ESM.docx]

Table S12 Gene-speciﬁc primer sequence for qRT-PCR detection

| Gene_ID | Gene Name | Forward Primer（5'-3'） | Reverse Primer（5'-3'） |
| --- | --- | --- | --- |
| JN105229 | *Actin* | ACTGCTGAACGGGAAATT | ATGGCTGGAACAGGACTT |
| Unigene23603 | *BCCP* | CTGTACCACCTGCCTTACCT | CACTTTATCTCCCACCTTGA |
| CL6932.Contig4_All | *BC* | GCTGTAGTAGAGATGAAGGAA | TGCGGTGTTGTATGTGTAA |
| Unigene20202 | *MCAT* | AACTCCCGCAAACTACAC | GCTATGAATTGGGACCTG |
| Unigene29386 | *KASII* | TGGCTGAGGTGAATGCTA | CAACTGAAGGCTCTGGATTA |
| Unigene37345_All | *KASIII* | CTTTCGCTTTGCTTGTCG | TATCACTCGTTCTGGTGGC |
| CL14443.Contig1_All | *FATA* | ACCTACATTGGCTGGGTTCT | AAACTGCTTCCGCCTCTT |
| Unigene26774 | *FATB* | GCGCAAAGGTTTAACTCC | TCTCCAAGATTGGCAAGG |
| CL15103.Contig1_All | *KCR* | TGAAGTGGATGAGAAGTTGT | GGTCGGAAGGAATAATAATGG |
| Unigene13977_All | *SAD* | CACTTGAACATGCACCTCCCG | AAATCTTTCCCTGTGTGTCTTCCGG |
| CL2636.Contig4 | *FAD2* | GCCAACAGTGTCATCAAC | TCCACCTCCTTCCTCATC |
| CL2686.Contig5 | *FAD3* | CTCTTCCCTCAAATCCCA | GGTCTTGATTGATGCTCC |
| CL13349.Contig2 | *FAD7* | GCCACAGGACTCATCATC | GACTTCTCTTCCACAGGTAA |
| Unigene30381_All | *GPAT* | CCATCCCAGCCGTTGCCTTGA | CCGATCACTCAACTCTGCGAATAAAGC |
| CL6160.Contig2_All | *DGAT* | AGAAGTTGGGATCTGAGTCAAGGTG | GGCAGACTTGTCTTAAACCGCACT |
| CL8973.Contig3_All | *OLE* | TTGACCACTGGGTTTCTTGCTTCC | CAAACTGCTCCGCCTTGTCCTT |
| CL6205.Contig10_All | *CLO* | AAGCATGGGAGTGACTCTGGAGC | CCCTAGCAAGAACATACAGAAGTCCC |
